# Effects of Circularity Interventions in the European Plastic Packaging Sector

**DOI:** 10.1021/acs.est.2c08202

**Published:** 2023-06-29

**Authors:** Ciprian Cimpan, Eivind Lekve Bjelle, Maik Budzinski, Richard Wood, Anders Hammer Strømman

**Affiliations:** † Industrial Ecology Programme, Department of energy and process engineering, Norwegian University of Science and Technology (NTNU), Trondheim 7491, Norway; ‡ Mobility and Economics, SINTEF Community, Trondheim 7491, Norway

**Keywords:** socioeconomic effects, scenario analysis, policy targets, plastic recycling, industrial ecology

## Abstract

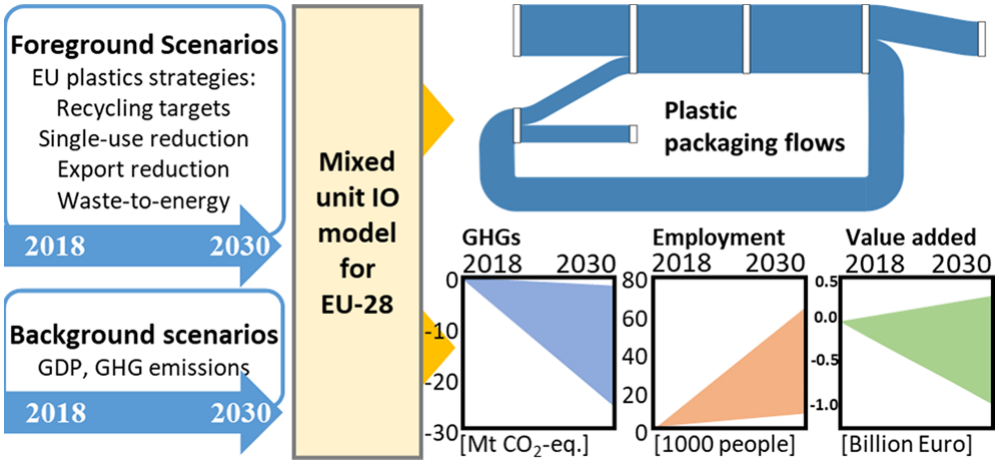

Low levels of plastics circularity today reflect major challenges for the sector to reduce environmental impacts and a need for wider systemic change. In this work, we investigated the potential for climate and socioeconomic benefits of circular economy (CE) interventions in the plastic packaging system. By means of a mixed-unit input–output (IO) model, we performed a comparative scenario analysis for the development of demand and waste management up to 2030 within the EU-28 (EU27 + United Kingdom). We modeled the development of material flows and assessed the effects of both demand-side and end-of-life interventions. Different levels of ambition toward 2030 based on EU circular economy strategies were tested. Results showed that on reaching high levels of circularity, between 14 and 22 Mt CO_2_-eq/year could be reduced by 2030 (20–30% of the total sector impact in 2018) compared to business-as-usual. Demand change (e.g., by decreasing product packaging intensities) showed similar emission-saving potential as achieving the current recycling target of 55%, which emphasizes the role of demand-side actions. Most scenarios displayed moderate employment gains and potential economic losses, pertaining to both direct and indirect activity shifts in the economy. While considering model limitations, the approach is useful in indicating potential first-order effects of system changes.

## Abstract 2

This work illustrates potential emission savings and socioeconomic effects induced by increasing the implementation of circular approaches in plastic packaging.

## 1 Introduction

The transition to a circular economy (CE) encompasses economy-wide changes affecting broad societal areas. The premise of such a transformation is economic development, which benefits business, society, and the environment, primarily by extending/perpetuating the productive stage of material resources, thus avoiding pressures connected to new resource exploitation.^1,2^ However, there is a lack of consensus on the magnitude of such ″win–win–win″ benefits.^3^ Global studies suggest that environmental impacts can be reduced, while employment opportunities can increase, driven by more labor-intense secondary sector activities as well as the increasing importance of services.^4^ However, the effects may be different per sector or by different circularity interventions.^5,6^ Thus, the use of material recirculation as a proxy for societal benefits may lead to unwanted consequences.^7^

Plastics overall and packaging as its main application are priority materials within CE strategies, especially in EU policy.^8^ However, plastics have currently very low circularity, with less than 20% overall recycling in the EU and around 5% input of secondary streams to closed-loop applications.^9,10^ Moreover, while the understanding of potential environmental effects of large-scale transition to circularity for plastics has improved substantially, socioeconomic implications, and particularly the potential for environment-economy tradeoffs, are understudied.

Increased scientific attention has expanded the understanding of societal flows by use of region-wide material flow analysis (MFA) (e.g., refs (11−13)). The possibilities to close physical plastic loops were assessed for the EU^9,14^ and even globally with a perspective on reducing plastic pollution.^15^ Recently, several studies evaluated region-wide circular economy strategies in the plastic sector from an environmental and/or cost perspective. Zheng and Suh,^16^ followed up by Meys et al.,^17^ used bottom-up process life cycle assessment (LCA) models to estimate global GHG emissions and evaluated mitigation strategies toward net-zero emission systems. They highlighted key challenges, such as a large need for renewable energy, biomass, efficient recycling, and demand-side measures. Using combined MFA and LCA frameworks, Chu et al.^18^ assessed when GHG emissions would peak in China for three polymers, and similarly Chaudhari et al.^19^ assessed energy and climate impacts in the United States (U.S.) but did not address system interventions. GHGs and costs/investments with end-of-life (EoL) options under different scenarios for the U. S. were assessed by Basuhi et al.^20^ In Europe, several studies addressed impacts of meeting policy targets, such as the recycling of packaging in 2030^21^ and 2025 targets for recycling and recycled content.^22^ The latter found that the total emissions of plastic use in 2018 were 208 Mt CO_2_-eq, which may be reduced by around 26 Mt CO_2_-eq by 2025. Direct employment and economic costs of interventions were assessed by Hestin et al.,^23^ and recently, Bassi et al.^24^ used LCA and societal life cycle costing (CLCC) to study the effects of PET packaging consumption in the EU to 2030, testing several waste management and consumption scenarios.

These studies take a high-resolution approach in terms of process system descriptions (and activities within study scope) and provide comprehensive assessments. Nevertheless, they have limitations in capturing indirect or supply chain effects that connect assessed activities to the rest of the economy.^25^ As will be shown in this work, environmental effects may be driven primarily by changes in (so-called) foreground activities, but a large portion of socioeconomic effects may occur in connected supply chains. Additionally, process-based LCA suffers from well-recognized systemic incompleteness due to truncation in inventory data (e.g., missing consumption of services).^26,27^

Environmentally extended input–output analysis (EE-IOA) has gained momentum in CE evaluation due to its ability for integrated assessment, linking economic development with environmental and socioeconomic aspects, within economy-wide or even global settings.^28^ Recently, studies showcased scenario tools based on EE-IO and their application to forward-looking assessments with a focus on energy/climate transitions and CE strategies.^29^ Examples include CE intervention scenarios in countries^6,30^ and globally.^4,5^ Cabernard et al.^31^ provided a first global analysis of environmental and socioeconomic footprints of plastic production and use, including their evolution to 2030, but without CE implementation. Nevertheless, some major limitations of applying EE-IO to CE assessment persist: (1) the standard monetary framework does not fully represent actual physical transitions in the economy, (2) weak or missing EoL stages, and (3) low material/product and sector resolution. Most CE policies are formulated in physical units (reduction, recycling targets). In response, hybrid and mixed-unit models were developed, starting with the well-known waste IO (WIO) approach to extending IO to include EoL,^32^ to economy-wide physical dimensions,^33,34^ and further disaggregating material/product systems.^35,36^ These IO approaches were used extensively to assess EoL systems, as well as broader CE strategies.^37−40^ However, hybrid approaches can lose the ability to maintain monetary balance, thus precluding measuring socioeconomic effects. With increased complexity, hybrid and monetary EE-IO models could supplement each other to assess CE indicators, as shown for Belgium by Geerken et al.^40^

This contribution showcases an IO model with the capacity to simulate physical circular system flows with economic accounting balance. The model was constructed to investigate environmental and socioeconomic effects of circularity interventions in EU plastic packaging consumption. While addressing a specific material, this work contributes also to the wider discussion on benefits/costs of the CE, specifically on challenges to the prevailing “win–win” perspectives. We intend to answer: to what extent could current strategies contribute to climate and socioeconomic benefits? And what are the potential tradeoffs of plastic packaging CE? A comparative scenario-based approach to 2030 was taken to answer these questions. We estimated the development of region-wide plastic packaging flows, potential GHG emissions, employment, and value creation, with different consumption and waste management interventions that embody current policy ambitions as well as system efficiency limits. The scenario analysis integrated the effects of (short-term) economic development, as well as changing “background” conditions, i.e., decarbonization of the economy.

## 2 Methods

### 2.1 Mixed-Unit Input–Output Model for the EU-28

We built a simple EE-IO model in mixed units,^35^ which distinguishes plastic packaging production, consumption, waste generation, and treatment, from the rest of the economy. Borrowing from LCA, we denote these activities as the foreground and the rest of the economy as the background system. While flows of the background system are measured in monetary units (million euro or MEUR), flows of the foreground, consisting of products and waste management services, are measured in both mass and monetary units. The foreground sections represent, essentially, disaggregated parts of the economy, with the model maintaining the original monetary accounting balances. The table structure is similar to the hybrid model by Nakamura et al.,^36^ with the distinction that we used a supply-use table (SUT) formulation^41^ instead of an input–output table (Figure S8 in the Supporting Information (SI) A). This was deliberate, as it supports the MFA perspective by the direct and explicit representation of both sectors (or activities) and products/services. Circular mass flows are endogenized in the model, reflecting substitution processes, as secondary plastics contribute input to packaging conversion in the foreground (denoted as closed-loop recycling), as well as to the production of other plastic goods in the background (denoted as open-loop recycling).

The underlying SUT was built starting from the EU-28 Eurostat tables for the year 2018 (NACE*64 industry level), as a single region with distinct representation of the use of imports. Although more sector aggregated than some multiregional IO (MRIO) databases such as EXIOBASE^42^ by comparison, Eurostat SUTs constitute an up-to-date base, which is consistent with production, trade, and EU-28 structural business statistics. EU intercountry monetary SUTs are now available with the FIGARO project.^43^ However, the single-region approach was prompted by the lack of data to describe foreground activities at the country level. Further, the foreground sections were disaggregated from parent sector/products by combining physical flow information, sector-specific economic data, and life cycle inventory data (see ref (44)). Finally, the table framework was completed by adding GHG emissions and employment extensions based on Eurostat.^45,46^ The approach and details on disaggregation are documented in the SI A Section 2.

To analyze the direct and indirect impacts of circularity interventions, we used the standard Leontief demand-driven modeling, in which the total output **x** required for a certain final demand **y** in a region or country is determined as **x** = (**I** – **A**)^−1^**y**, where **L** = (**I** – **A**)^−1^ is the Leontief inverse, **A** is the matrix of technical coefficients, and **I** is an identity matrix of size **A**.^47^ This allows us to calculate the overall upstream supply chain impacts (or footprints) induced by the consumption of goods and services, as **c** = **b L y**, where **c** are footprints for different metrics (here GHGs, employment, and value added) induced by final demand and **b** represents vectors of sectoral intensities for the metrics. We point out that here **A** was a compound square coefficient matrix in supply-use formulation, determined with the industry-technology assumption.^41,48^

With IO frameworks, both static and temporally dynamic scenarios can be modeled by the implementation of exogenous changes in (1) the structure and size of final demand, (2) changes in the matrix of technical coefficients, and (3) changes to value added components and to environmental and social extensions.^5,49,50^ The effects of interventions implemented in scenarios can be measured simply as Δ**c** = c* – **c**, where c* = b* L* y* is the footprint outcome based on potential changes (*) in the sectoral intensities (**b**), technical coefficients (**A**), and/or final demand (**y**). The results represent a comparison between a reference and scenarios in which the interventions, *ceteris paribus*, have been achieved.^49^ Importantly, in the mixed-unit system, the plastic mass flows after interventions can be determined by recalculating interindustry flows (the transaction matrix) Z* = A* diag(L* y*), where “diag” refers to the diagonalized matrix. Finally, while Δ**c** represents the net difference between scenarios, the total impact of the plastic packaging system in isolation from the rest of the economy can be estimated by placing a demand-pull **y** equivalent to the total consumption of converted packaging in a year and for associated waste management services (with all remaining demand for products set to zero). The result is total impact under the domestic technology assumption, i.e., import flows are accounted as produced/waste handled with domestic technology.

### 2.2 Triple Bottom Line and System Efficiency Indicators

Circularity interventions induce environmental and socioeconomic effects. The present work used well-established indicators to measure these effects. Value added is a conventional indicator for representing economic impacts, as it represents the production-side calculation of GDP.^30^ Employment indicates socioeconomic effects; however, we do not detail changes in skill level and other social aspects. For environmental aspects, GHG emissions are a reliable indicator, which also links CE to the wider climate transition but nevertheless does not capture potential burden shifting between different impacts.

The plastic mass flows in the model were used to measure the overall efficiency of the plastic packaging system by two widely used indicators. The recycling rate (RR) in a year expresses the percentage of plastic waste supplied to the market as secondary plastics, thus, after full reprocessing or secondary plastic production. The RR point of measurement here differs slightly from the new EU requirement, which precludes some final reprocessing steps.^51^ The second indicator is a closed-loop circularity rate (CR) that expresses the percentage of packaging conversion demand covered by recycled plastics. While closed loop is generally defined as plastic flows pertaining to a specific product group being recycled into the same, here we more broadly use it to encompass the use of secondary plastics in the production of new packaging vs their use in all other sectors (open loop).

### 2.3 Mass Flows Underlying the Foreground System

The model required establishing the base system flows for 2018, which is the starting year for scenario simulations. Mass flows were calculated with a resolution of seven polymer types (thermoplastics) and a group denoting the remaining types. Production stages, including primary plastic production and conversion/manufacturing of packaging, were based primarily on Plastics Europe.^52,53^ The consumption and waste generation stages within the EU-28 economy were described using the IO-MFA approach,^33,54^ i.e., monetary intersectoral and sector-final demand flows served as a vehicle for the estimation of packaging flows and waste generation, considering that a certain amount of packaging is associated with goods/services produced and used throughout the economy or internationally traded. In the present model, this amount was calculated with product packaging intensities (measured in kt/kt or kt/MEUR). An elaborate attempt to determine intensities for the EU was performed earlier by ref (10). Here, intensities were derived in a simpler manner by using the detailed U.S. SUT,^55^ a reasonable proxy for the EU.

Waste streams generated at multiple points in the process chain were accounted: (1) production waste, (2) preconsumer waste (packing/manufacturing, transport/wholesale-retail, and service waste), (3) postconsumer waste (in sectors and final demand), and (4) secondary waste (sorting rejects, incineration residues). Pre- and postconsumer wastes were denoted in the following as plastic packaging waste (PPW). Polymer-specific flows within collection, sorting, and recycling stages for 2018 were calculated with data from several recent reports commissioned by Plastics Recyclers Europe,^56−58^ as well as other sources (e.g., refs (53, 59)).

Packaging production and waste statistics at the EU level^52,60^ reflect a large unsupported gap of around 15%, which is generally treated as addition to stock in MFAs.^12,53^ Accordingly, in 2018, packaging production for EU-28(+2) stood at 21,000 kt, while PPW stood at 17,800 kt. Although several causes contribute, there is increasing confidence that packaging consumption and waste are underreported in many EU member states.^61^ In the present model, we used a different approach whereby PPW generation is determined as the sum of domestic use + net trade – stock additions. The latter accounts for the potential stock additions due to delays between consumption and disposal. This resulted in a PPW generation closer to 19,500 kt. The complete 2018 mass balance is illustrated in Figure S2. Data sources and approach to mapping physical flows are detailed in the SI A.

### 2.4 Scenarios to 2030

We developed two reference scenarios and three intervention scenario narratives for the development of packaging consumption and waste management to 2030. In the following, we distinguish between the background frame, describing the overall evolution of the EU economy, and the foreground scenarios, which contain the specific circularity interventions studied.

#### 2.4.1 Background Frame

Considering the short time horizon, we developed a single background frame scenario, driven by exogenous macroeconomic data.^62^ The scenario was implemented by scaling final demand components without structural changes.^63^ For the EU-28 GDP development between 2019 and 2023, we used the 2021 short-term economic forecasts by the European Commission, which includes the effects of the Covid-19 pandemic.^64^ For the subsequent period up to 2030, we used the approach of Scott et al.,^65^ i.e., we applied econometric trends in the projection of the different components of final demand. World GDP projections up to 2025 were available from the IMF World Economic Outlook Database, and up to 2030, we applied growth rates in line with the shared socioeconomic pathway SSP2—middle of the road.^66^

The second major component of the background frame was the potential development of GHG emissions. To reflect EU decarbonization efforts, we (1) projected sectoral GHG emission intensities following historical trends (2008–2019) given by linear least-squares regression^67^ and (2) the intensity for power and heat production was reduced, consistent with levels indicated by the European Environment Agency, which would allow the EU to achieve a net 55% reduction (from 1990 levels) in GHGs by 2030. With this approach, total GHGs in the EU changed over the period roughly in line with the EU Reference Scenario 2016, displaying a decrease of approx. 20%.^68,69^

#### 2.4.2 Foreground Narratives

We first defined two reference scenarios that constitute the baseline for comparison to intervention scenarios. The references differ in the way product packaging intensities are treated. In the first, intensities are maintained constant; thus, packaging flows evolve proportionally with demand of goods/services, while in the second, a decrease of 1% per year was implemented to reflect dematerialization (based on evidence over 25 years^70^), as well as potential effects of societal pressure to transition from plastics. Savings due to packaging decrease were reallocated as an increase in sector research/innovation, and waste management savings were added to value added.

The foreground intervention scenarios were built around the main quantitative targets within EU policy, as well as general directions given in the EU Strategy for Plastics in a Circular Economy.^8^ Interventions modeled (i) increased recycling (a product of collection, sorting, and reprocessing), (ii) reduced the consumption of single-use packaging (SUP), (iii) reduced exports of recovered plastics, and (iv) increased closed-loop recycling. The intervention scenarios were modeled within both references.

The overall scenarios then were:

(0) Reference or business-as-usual (BAU)—scenarios with constant or decreasing product packaging intensity while the foreground is unchanged.
(1) Baseline development—this scenario narrative projected recycling over the period based on historical precedent. The time series for packaging waste recycling from Eurostat was used as a predictor. SUP consumption reduction measures were in line with the EU directives.^71,72^ Exports followed a 10% reduction per year, in line with developments between 2018 and 2020. The ratio of secondary plastics returned to the packaging sector remained constant. The landfilling ratio of plastic waste decreased from 40% to 20%, in line with historical development.^60^
(2) EU targets—this normative scenario narrative implemented the current targets for 50% recycling by 2025 and 55% by 2030.^73^ SUP reduction measures were in line with the adopted legislation. Exports followed a 10% reduction per year. Closed-loop recycling (to packaging) increased to satisfy the mandated and pledged recycled content rate of 30%.^71,74^ The landfilling ratio decreased further from 40% to 10%, in line with targets for the reduction of municipal waste landfilling.^75^
(3) Max potential—this scenario narrative increased the recycling rate to 70% by 2030, deemed the maximum possible by several studies.^76,77^ SUP reduction measures were in line with the adopted legislation. Exports followed a linear reduction per year to achieve complete elimination by 2030. Closed-loop recycling increased to 90%. The landfilling ratio decreased from 40% to 10%. This scenario was guided by plausible efficiencies, assuming best-available techniques (separate collection,^21^ sorting, and reprocessing efficiencies in European plants^9^), as well as inherent packaging design changes (not directly modeled).

The effects of SUP consumption reduction measures based on the targets of the SUP Directive and the Carrier Bag Directive were estimated at a potential of 1000 kt or the equivalent of 5% of plastic packaging used in 2018. SUP reduction was modeled by reducing direct consumption of packaging by final demand and compensated with increased expenditure for paper products.

Each of the (1–3) scenarios was modeled as a group of five subscenario variants, whereby increased recycling constituted the core intervention, and the remaining interventions were added individually and eventually combined. The scenario variants were (a) efficiency changes toward recycling, (b) recycling + SUP reduction, (c) recycling + export reduction, (d) recycling + increased closed-loop, and (e) combined interventions. The system was projected every year until 2030, and the adoption of the different interventions was implemented in a linear manner. Additional description of scenario development is available in the SI A Section 3.

## 3 Results and Discussion

### 3.1 Plastic Packaging Flows and Circularity Potential toward 2030

With domestic demand for packaging in 2018 at 21,000 kt, and estimated PPW amounting to 19,500 kt (flows in Figure S2), separate collection systems in Europe captured for recycling around 38% of the waste generated. Around 30% of this was sorted and sent to recycling within (22%) or outside Europe (8%). After final processing stages, recovered secondary plastics constituted around 18% of the original generated waste (domestic recycling rate RR). Less than one-third (5%) of this contributed to new production of packaging (closed-loop circularity CR).

**Figure 1:**
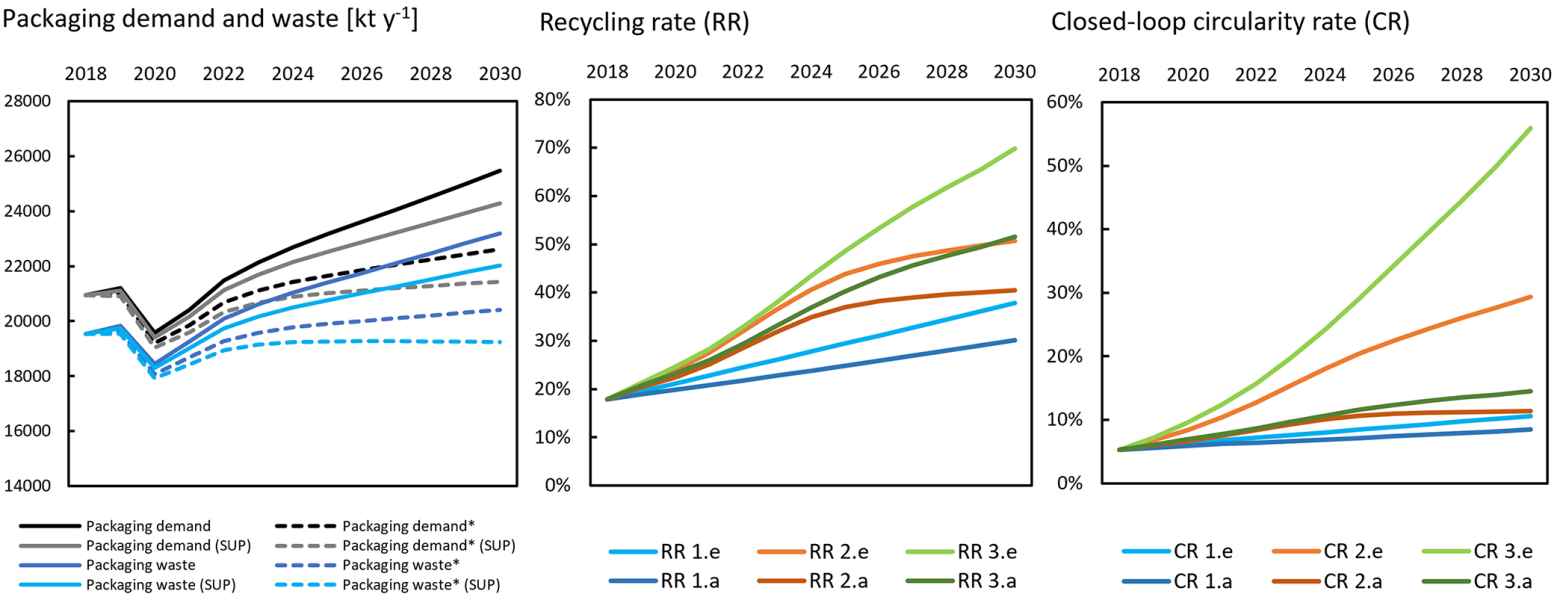
(Left) Evolution of the domestic packaging demand and associated packaging waste between 2018 and 2030; dotted lines and full lines denote reference flows with and without packaging intensity decrease and SUP denotes scenarios with mandated reduction. (Center and right) Evolution of domestic EU recycling (RR) and recycled content (CR) in packaging conversion (.a - recycling interventions; .e - all interventions) from the level in the reference year 2018.

In the two reference scenarios, domestic packaging demand was projected to first decrease in 2020 due to the Covid-19 pandemic and then to recover during 2021–2022. Figure 1 (left) illustrates the development up to 2030. In the absence of interventions, packaging demand over the period increased by 8–22% and PPW by 4–19%, with the lower ranges in the reference accounting for product packaging intensity decrease. When the consumption reduction of SUP was included, the demand increase was limited to 2–16% and PPW decreased by −2% or increased up to 16%. For comparison, the recent study by Antonopoulos et al.^9^ projected PPW by 2030 at 22,000 kt, which is in the higher range here. A widening gap between demand and PPW was observed, caused by a faster increase in exports of packaged products compared to imports. This follows past trends and reflects faster economic growth in extra-EU regions in the current decade.

Figure 1 (center/right) follows the development of system efficiency indicators. We distinguish the scenario variants implementing only recycling and the ones implementing the full narratives (all interventions). Results highlight the importance of export reduction (of recovered plastics) on the domestic RR. In the EU targets scenarios, the overall RR reached 55% by 2030 but domestically only 40–50% (depending on the export rate). RR reached its domestic target of 70% only in the maximum potential scenario with exports fully eliminated. The same scenario showed that with an RR of 70%, the contribution of secondary material (CR) could reach 55% of converter demand by 2030.

**Figure 2:**
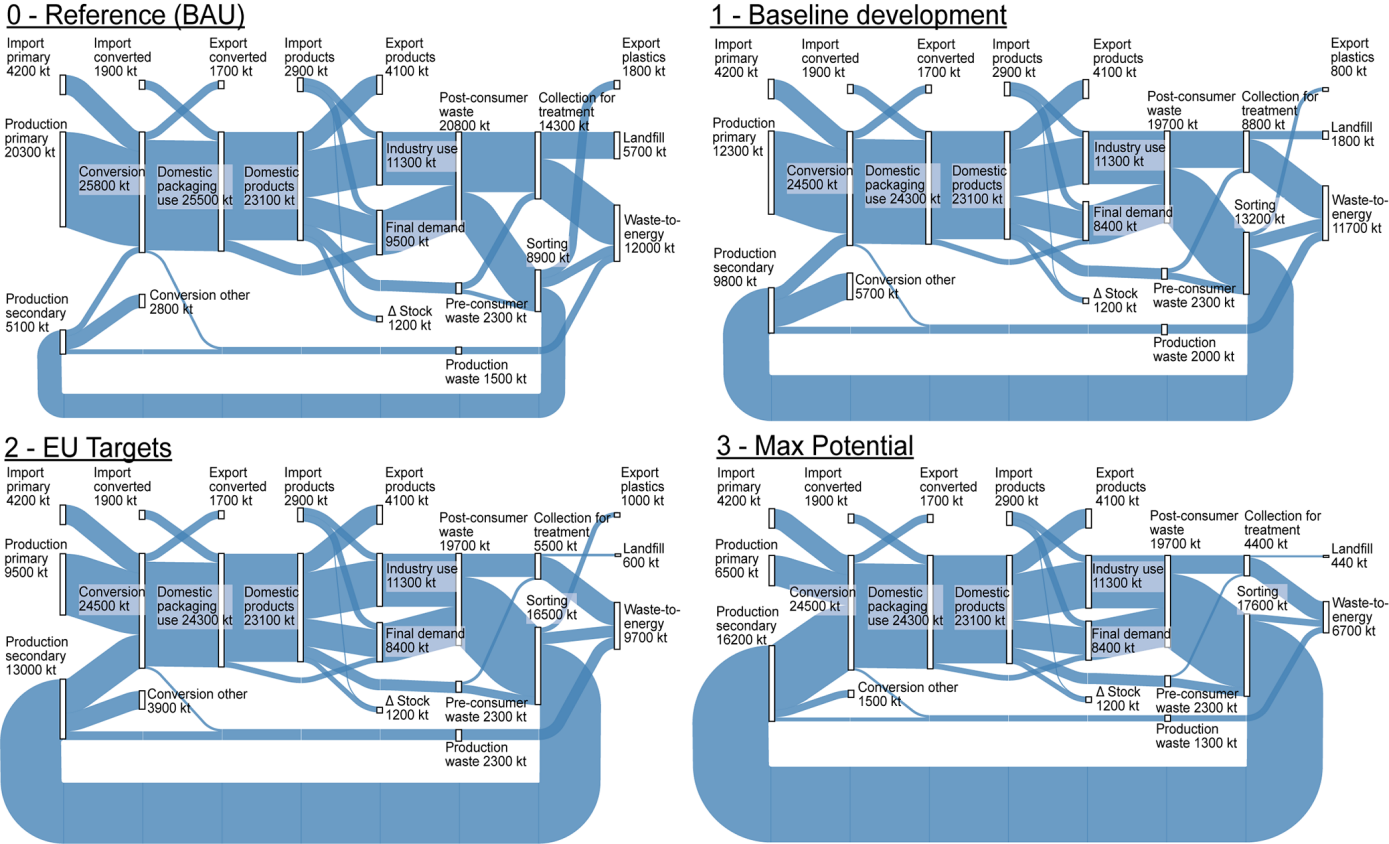
EU-28 flows for the plastic packaging system in 2030 [kt y^–1^]; scenarios without a packaging intensity decrease (equivalent Sankey diagrams for the systems with a decreasing packaging intensity are available in the Supporting Information A). The scenario variants (1–3) include the application of all interventions studied (variant e). Values denote process totals and are rounded to two/three significant digits. The flow data underlying the figure is available in the Supporting Information B.

The material flow balances in 2030 for the reference and the three intervention scenarios, with constant packaging intensity, are illustrated by Sankey diagrams in Figure 2. The equivalent figures with decreasing packaging intensity are found in the SI A. The figures highlight the growing role of waste-to-energy (WtE) at the expense of landfilling, a decreasing direct use of SUP by the final demand of over 1 Mt, as well as the extreme system transformations needed to achieve the current targets of EU policy and beyond. Roughly, there is a need for a threefold increase in recycling output until system limits would be reached.

### 3.2 Environmental and Socioeconomic Effects

**Figure 3:**
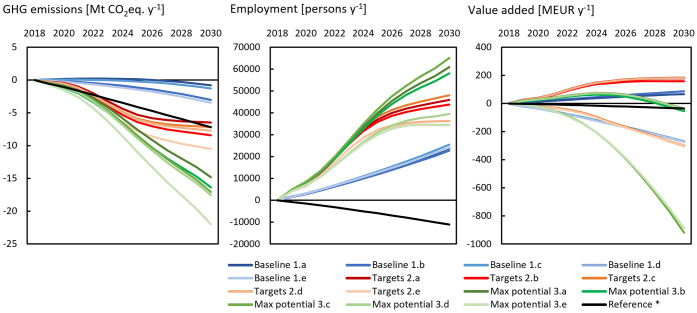
Net difference between intervention scenarios and the reference (BAU) scenario (represented by the zero line) over the 12 year period: GHG emissions, employment, and value added. Scenario variants are noted a–e. Results are for scenarios with constant packaging intensity; the reference with an intensity decrease is shown with a black line.

Circularity interventions led in the different scenarios to significant GHG emissions reduction and more moderate employment gains concurrent with minor losses of value added. Net differences between intervention scenarios and the reference (Δ**c**), under constant packaging intensity, are illustrated in Figure 3. In the same figure, the difference between the two references was also highlighted (black line), revealing that a decreasing packaging intensity alone could contribute to around 7 Mt CO_2_-eq. savings by 2030, as well as lead to a small loss of employment and minor negative value added.

Extending past progress, emission savings in the baseline development narrative followed closely the reference, achieving only a minor 1–3 Mt CO_2_-eq. savings by 2030. The reason for this was that recycling improvements were (initially) unable to compensate for emissions from the increasing use of WtE (to the detriment of landfilling). However, eventually benefits appeared, in part due to the diminishing capacity of WtE to save emissions by energy production (as background production decarbonizes). Meeting EU targets and beyond (max potential scenario) led to savings of 6–10 Mt CO_2_-eq. and 14–22 Mt CO_2_-eq. respectively. The lower range accounted for changes in recycling alone (.a), while the upper range pertains to full narrative implementation (.e). For perspective, the maximum emission savings shown correspond to 0.74% of total industrial emissions in 2018 EU-28 (3,570 Mt CO_2_-eq.^45^) or the total country emissions of Lithuania in the same year (23 Mt CO_2_-eq). Although not completely comparable, GHG savings fall in the range of some recent studies generally employing process-based approaches. Tallentire and Steubing,^21^ for example, found that a 50% recycling of PPW (of 16.5 Mt) in the EU resulted in around 7 Mt CO_2_-eq. savings, while ref (78) and ref (22) showed that an additional collection for recycling of 10 Mt plastics in the EU would save 20–26 Mt CO_2_-eq. The difference in collection for recycling between our reference and max potential scenario is 9 Mt PPW, with the latter achieving 14 Mt CO2-eq. savings by recycling improvements alone.

We also confirm an aspect reported by Bassi et al.^24^ for the EU PET system, specifically that increasing consumption with efficient waste management does not necessarily result in lower environmental impacts compared to lower consumption and weak recycling. As can be seen in Figure 3, the targets scenario decreased emissions roughly equivalent to the reference with packaging intensity reduction.

All intervention scenarios added employment compared to the reference, specifically in 2030 reaching 22–24,000 person-positions with baseline development, 34–48,000 in the targets scenario, and 34–65,000 in the max potential scenarios. In the latter two scenarios, implementing all interventions (.e) led to lower-range employment gains, which is elaborated in the following contribution analysis. Results also indicated minor value added gains in scenario variants with increased recycling, which turned to losses in variants reducing exports (.c) and combining all interventions (.e).

#### 3.2.1 Contribution Analysis

**Figure 4:**
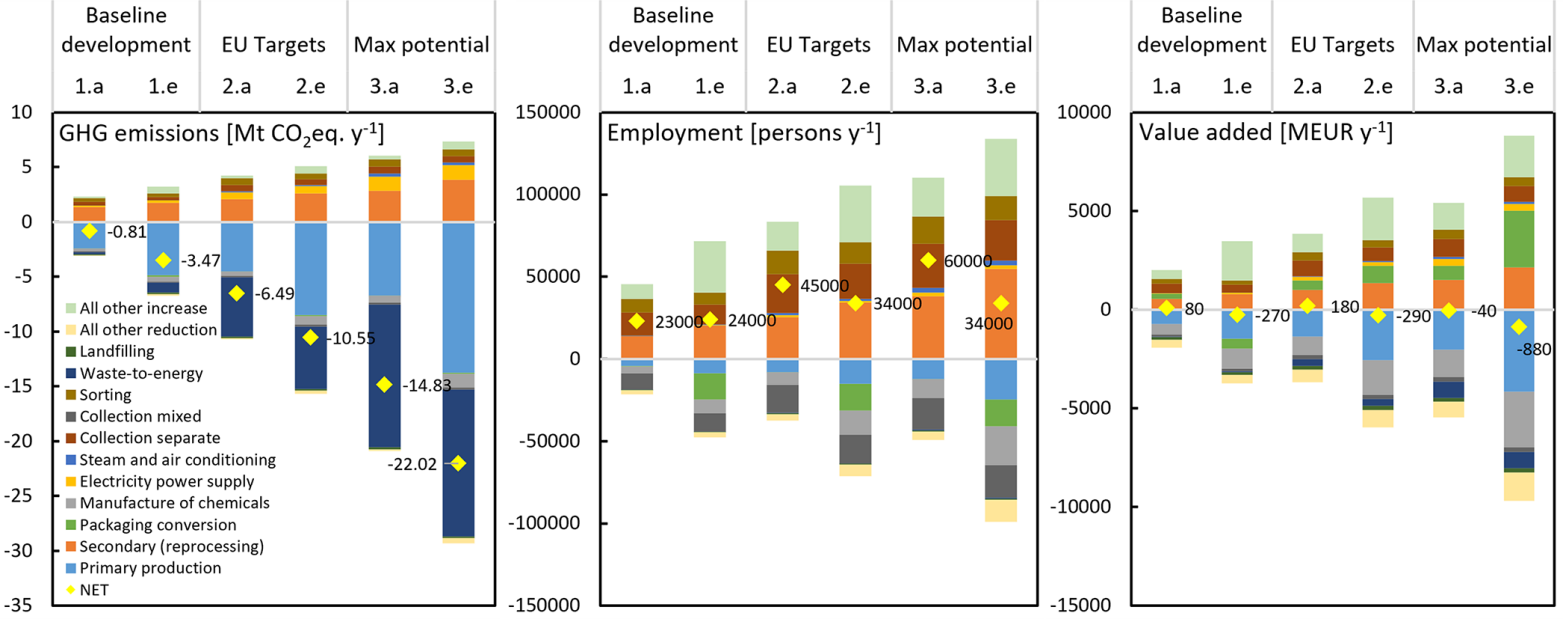
Sectoral contribution to GHG emissions, employment, and value added in scenarios with constant packaging intensity. The results are calculated as difference in 2030 from the reference (BAU) scenario (represented by the zero line) (.a - recycling interventions; .e - all interventions). Results for the scenario alternatives with/without packaging intensity decrease for all five subscenarios are available in the Supporting Information A and B.

Sector contributions to the net difference illustrated in Figure 3 are captured in Figure 4 for the end year. They reveal important reallocation effects between sectors, especially regarding employment and value added creation. The contributions to GHG emissions, both reductions and additions, were dominated by foreground sectors (88–95%) in all scenarios. Conversely, only 65–80% of contributions to employment and value added differences pertained to foreground sectors, indicating much stronger indirect effects through upstream value chains.

Employment gains reflected a shift between sectors with lower employment intensity, namely, primary production and upstream manufacture of chemicals, to activities with higher intensity, such as collection, sorting, and reprocessing.^79^ Contributions to value added revealed losses by primary production, upstream chemicals production, and WtE sectors, which generally add more value per unit production than operations toward recycling. Taking specific interventions, both employment and value added loss due to SUP reductions were mostly compensated by the shift to paper-based products. Reductions of exports led to an increase in employment due to additional recycling infrastructure, concurrent with a decrease in value added due to displacement of primary production. An interesting effect was observed regarding packaging conversion, which lost employment due to decreasing demand (for SUP) but saw substantial increases in value added in scenarios with large closed-loop recycling. This was due to a decreasing total cost of feedstocks, as secondary plastics have a lower price than primary plastics in the model. Under current recycled content policies, the average price of secondary plastics may increase. To test sensitivity to the fixed price assumption, we ran scenarios whereby prices were gradually increased to the primary level and the secondary sector turnover was upscaled accordingly. As somewhat expected, losses in value added in the conversion sector were mostly compensated by increased inputs in the secondary sector, and there were also associated small gains in employment as well as small increases in GHG emissions (result illustrated in Figure S29).

We note here that as linear models do not consider dynamic price responses, we may overstate these effects in the economy. Nevertheless, results do reflect potential for reallocation effects across supply chains compared to studies capturing only direct effects. An example is lower gains in employment shown here compared to Hestin et al.^23^ or the job creation potential indicated with PET circularity in ref (24).

#### 3.2.2 Total Sector Footprint

**Figure 5:**
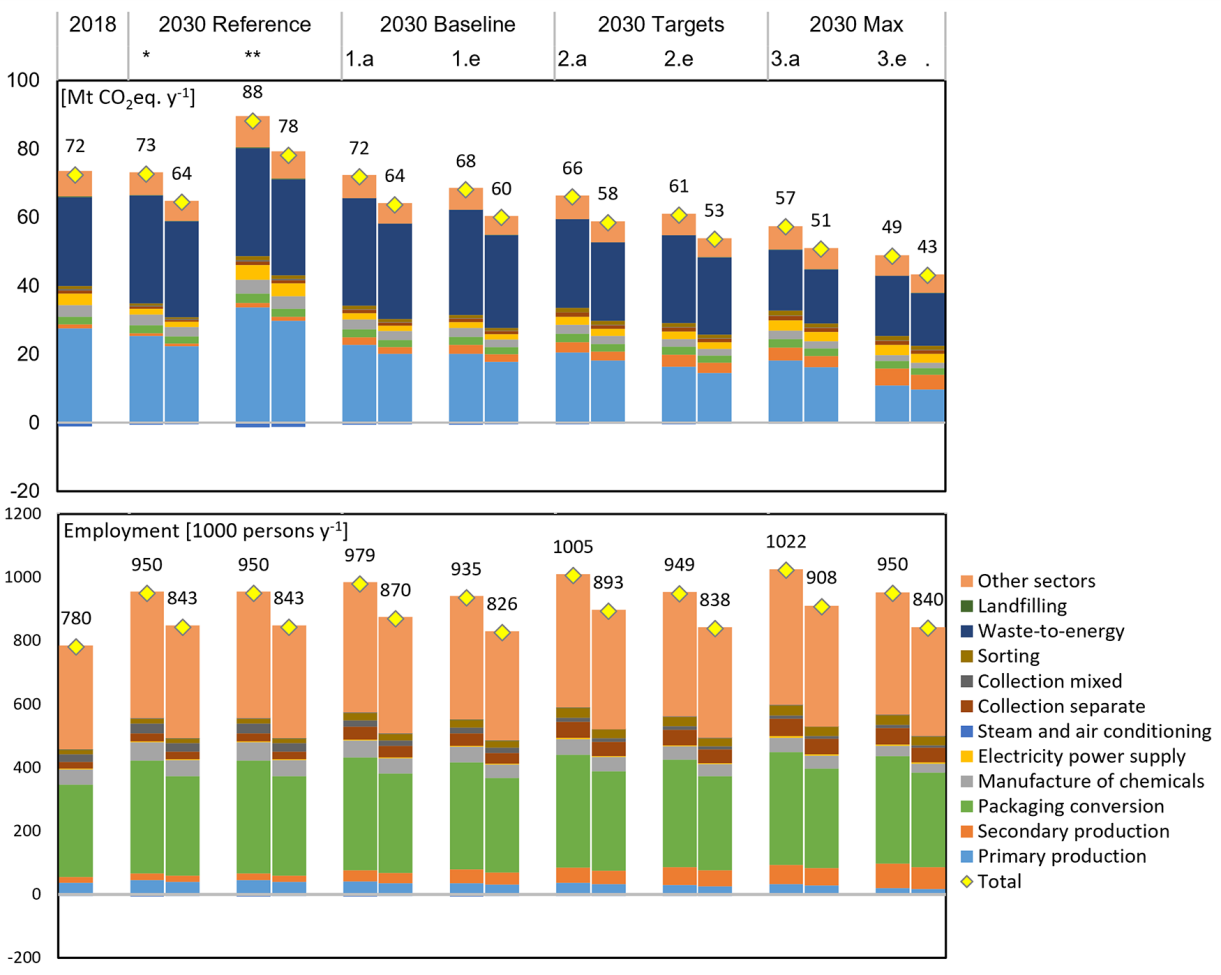
Total (direct and indirect) GHG emissions and employment of the plastic packaging system and main activity contribution. Each scenario is illustrated as two stacked bars; the right and left bars represent results for the system with and without packaging intensity decrease, respectively. * and ** denote the reference scenario in 2030 with and without the change in sectoral direct emission intensities, respectively; .a – recycling interventions, .e – all interventions. The equivalent figure for value added is available in the Supporting Information A. Sectors that contribute indirectly (not part of the packaging system) are the manufacture of chemicals, electricity power, steam and air conditioning, and other sectors (sum of remaining background sectors).

The total (direct and indirect) impact of the plastic packaging system in 2018, in isolation from the rest of the economy, was estimated to be around 72 Mt CO_2_-eq (Figure 5). The system also contributed to the employment of 780,000 persons, of which 48% was in upstream industries, and around 56,000 MEUR value added to the economy, with more than 50% occurring in upstream industries (Figure S16). For comparison, Plastics Europe estimated that the entire plastics industry in Europe employed directly over 1.6 million people and had a turnover of more than 360,000 MEUR.^52^

In the reference scenarios, overall system GHG emissions remained stable over time with constant product packaging intensity and decreased by 8 Mt CO_2_-eq with decreasing packaging intensity. The main sector contributions changed substantially to 44% for WtE (36% in 2018) and 37% primary production (41% in 2018). This reflects the changes in background emission intensities, without which the total emissions of the reference systems in 2030 would have increased to 88 Mt and 78 Mt CO_2_-eq. Intervention scenarios lowered total emissions to a minimum of 67% of the reference scenarios. We note here that negative emissions observed in the system footprint pertain to WtE heat production, of which the system consumed overall less than it produced.

Finally, we want to point out that emissions, employment, and value added differences in Figures 5 and S16 between the two scenario sets (constant vs decreasing packaging intensity) are different from the net differences indicated in Figure 3. Specifically, Figure 5 shows an overall decrease in employment by around 100,000 person-positions and a 7000 MEUR decrease in value added (Figure S16) between the two reference scenarios in 2030, compared to a net difference of 10,000 person-positions and a negligible loss of value added in Figure 3. This underscores two different perspectives, as Figure 5 illustrates system footprints, while Figure 3 captures net changes in the entire EU economy. The latter includes reallocation effects (outside the foreground system), such as an increase in other packaging materials due to SUP reduction, as well as potential growth in research/innovation to achieve packaging intensity decreases. This essentially compensated employment losses by 90% and value added almost entirely.

### 3.3 Limitations and Perspectives

The present work showcased several distinct advantages of mixed-unit IO models, as well as some important limitations. While (monetary) EE-IO models can reflect changes in the economic structures with the implementation of CE,^4−6^ they cannot quantify impacts on material flows, such as changes in waste flows and waste handling systems. This is achieved with hybrid IO models but so far largely at the expense of assessing socioeconomic impacts (e.g., refs (37, 39)), as it becomes difficult to maintain economic balances. For instance, changes in waste amounts or shares of different waste treatments affect industry and household costs, which then have to be integrated into use and final demand. In the present model framework, all physical flows have associated values (or prices), and the effects of changes in physical flows reflect back in the economy. This was possible due, in part, to the study focus on one sector or system, plastic packaging, for which industry links and EoL stages could be reasonably well described in Europe. As such, the present model can indicate potential tradeoffs between environmental and socioeconomic aspects but from a single-region perspective, i.e., without determining impacts in regions outside the EU affected by circularity interventions within the EU. We aim to address this in future efforts but note here that current MRIO databases still suffer from low monetary sectoral and country resolution in “rest-of-the-world regions” due to a lack of available data.^80^ Moreover, much of the fate of materials, waste, and cost structures of waste management are unknown.^81,82^

There are known limitations with EU IO data. SUT data is consolidated and aggregated at the EU level from country tables, and differences between accounting or missing data in certain country tables require applications of gap filling and balancing procedures.^83^ This is a source of uncertainty more broadly characterizing all IO databases. Multiregional models link countries and regions and typically require additional rebalancing, resulting in potential significant errors/deviations from reality.^84^ Assessment of potential issues with the monetary tables was outside the scope of the present work. In addition, the mixed-unit IO model remains coarse in sector representation and is subject to uncertainties pertaining to aggregation.^27,85^

Furthermore, EE-IO models (as well as LCA models), as linear structural models compared to dynamic economic models, cannot directly reflect repercussions of price responses, substitution elasticities, or changes in international trade structures. In addition, they do not capture behavior aspects or potential for rebound effects. Recent studies have found significant rebound potential around CE strategies.^86,87^ These occur as CE may generate monetary savings and induce substantial investments, leading to changes in consumption. Contrastingly, dynamic economic models generally use more aggregated representations of the economy, precluding detailed CE intervention analyses,^50^ as well as having rather rigid assumptions of agent behavior. Nevertheless, there are opportunities for further developments around integrating dynamic aspects in IO, as shown by Wiebe et al.^30^ with endogenous consumption and investments or by Vivanco et al.^88^ with the integration of rebound effects. To conclude, as Wood et al.^49^ point, the results of IO models provide first-order impacts, across supply chains, which are useful for policymakers/evaluators, precisely because they are devoid of assumptions on dynamic effects. The scenario results presented here should not be taken as absolute values but used as indicators of potential effects, considering the model limitations and data uncertainty.

### 3.4 Circular Economy and Policy Implications

Bearing in mind the scope of this study, i.e., plastics used in packaging, which accounts for roughly 40% of plastic applications, we found that current approaches to increase circularity could lead to significant yearly emission reductions, with up to 120 Mt CO_2_-eq. when cumulated to 2030. Overall, we found more modest but positive effects on employment and small negative value added effects. We point, as other researchers,^24,30^ to the role of consumption, or demand-side interventions, in driving potentially substantial emission savings. Nevertheless, we warn that savings by dematerialization and SUP substitution in packaging are dependent on reallocation effects and the impact of induced/avoided activities. Moreover, emission reduction may be joined by significant employment and economic losses, although both were found to be small here. The implementation of demand-side interventions is particularly challenging and may be countered by rebound effects. Past developments certainly indicate that packaging demand did not deviate significantly from real GDP development in the EU (*R*^2^ of 0.834; Figure S11), despite increasing pressure on the sector. We also emphasize that the results and conclusions of this work should be weighted, considering limitations and uncertainty discussed in Section 3.3.

Interventions to increase recycling, including closing loops in the packaging sector, reflected substantial reallocation of employment and value added in the economy. This may, in general, be beneficial for employment due to higher rates in secondary sector activities compared to those in primary, but absolute benefits may be lower than expected when accounting only for direct sector employment.^23^ In addition, we point again that this work did not consider impacts outside the EU. The evaluation of effects on economy is more difficult. As the manufacture of chemicals and primary plastics in Europe is characterized by high value added, results showed that economic losses were possible but were to a large extent compensated by gains in secondary sectors. Several IO-based studies have indicated that CE is not in every case an environment-economy “win–win”.^5,6^ From a policy perspective, these studies indicate possible weak points, which need to be supported. Dynamic macroeconomic models may be used to test further financial or economic strategies.^3^

WtE for plastic waste, especially for residual streams, is likely to remain a significant option in the future. It does, however, increase critical challenges. Despite the displacement of WtE by recycling, remaining combustion emissions could still account for 22–25 Mt CO_2_-eq. or 39–43% of the total impact of the system in 2030 (based on the targets narrative). Therefore, additional efforts will be needed, eventually by amending of WtE with carbon capture and storage/use (CCS/U)^89^ technologies.

While so far IO-based studies of CE approaches have treated secondary–primary materials as equivalent,^4,5^ the topic of quality and its impact on substitution are central to much research.^90−92^ In the mixed-unit framework, the so-called value-corrected substitution can/was to an extent endogenized here, as secondary plastics have a lower average monetary value. The increase of closed-loop recycling benefited the climate perspective by displacing more primary production, at the expense of some employment, but value added was less impacted due to gains in the conversion sector.

Lastly, given that several studies explored the technical possibilities and potential implications of high circularity in the plastic sector, a pertinent question arises—how realistic is it that Europe will meet its ambitious targets? No doubt, to realize a circular system, we must overcome many technical, policy, and behavioral barriers.^93^ High circularity scenarios rely on dramatic system efficiency improvements. Wide cultural and socioeconomic differences between EU regions, as well as most areas with high population density, pose great challenges to citizen-driven plastic recovery.^94^ Moreover, the Covid-19 pandemic and the more recent conflict in Ukraine once more brought forward the vulnerabilities of recycling systems to macroeconomic shocks (particularly to fossil fuel and energy prices).^95^

We conclude by naming several priority areas, in our view, crucial to achieving sustainable CE in the packaging sector. First, we praise efforts in the EU to improve consumption and waste statistics, as these are crucial to relevant assessments, policy development, and monitoring of progress. We point, however, to remaining serious issues with data on consumption and EoL of plastics. To counter potential employment and economic displacement, regulatory interventions such as a carbon tax and virgin material fees should be prioritized. These would also increase the overall resilience of the secondary sector. Effects on extra-EU regions should be considered if the aim is an overall socially and environmentally just CE transition. Further, actual implications of material quality and the limitations of citizen/business behavior should be addressed more thoroughly in policy making. Last, the largely ignored aspect of CE rebound effects, for example, induced by material price changes and demand dynamics, needs to be addressed both by policy and further research.

## Supporting Information Available

Additional data and methods description: structure of the plastic material flows in the model, distinguishing sections of upstream production, consumption, and waste management; closeup of packaging flows in consumption; the data sources and processing steps involved in building the base hybrid IO model; and additional result figures (PDF)
Data presented in figures in the manuscript; scenario time series; product packaging intensities; preconsumer waste rates; and 2018 SUT in mixed units (XLSX)

## Abbreviations

BAU: business-as-usual
EE-IOA: environmentally extended input–output analysis
EoL: end-of-life
GDP: gross domestic product
GHG: greenhouse gas
IO-MFA: input–output material flow analysis
LCA: life cycle assessment
MRIO: multiregional input–output
SUP: single-use packaging (distinguished from all single-use plastics)
SUT: supply-use table
WtE: waste-to-energy (mainly incineration with energy recovery)

## References

[ref1] World Economic Forum; Ellen MacArthur Foundation; McKinsey & Company. The New Plastics Economy — Rethinking the Future of Plastics, 2016.

[ref2] Ellen MacArthur Foundation; McKinsey Center for Business and Environment. Growth within: A Circular Economy Vision for a Competitive Europe. Cowes, 2015.

[ref3] Aguilar-Hernandez G. A.; Rodrigues J. F. D.; Tukker A. Macroeconomic, Social and Environmental Impacts of a Circular Economy up to 2050: A Meta-Analysis of Prospective Studies. J. Cleaner Prod. 2021, 278, 123421 10.1016/j.jclepro.2020.123421.

[ref4] Wiebe K. S.; Harsdorff M.; Montt G.; Simas M. S.; Wood R. Global Circular Economy Scenario in a Multiregional Input–Output Framework. Environ. Sci. Technol. 2019, 53, 6362–6373. 10.1021/acs.est.9b01208. 31051078

[ref5] Donati F.; Aguilar-Hernandez G. A.; Sigüenza-Sánchez C. P.; de Koning A.; Rodrigues J. F. D.; Tukker A. Modeling the Circular Economy in Environmentally Extended Input-Output Tables: Methods, Software and Case Study. Resour., Conserv. Recycl. 2020, 152, 104508 10.1016/j.resconrec.2019.104508.

[ref6] de Boer B. F.; Rietveld E.; Rodrigues J. F. D.; Tukker A. Global Environmental and Socio-Economic Impacts of a Transition to a Circular Economy in Metal and Electrical Products: A Dutch Case Study. J. Ind. Ecol. 2021, 25, 1264–1271. 10.1111/jiec.13133.

[ref7] Harris S.; Martin M.; Diener D. Circularity for Circularity’s Sake? Scoping Review of Assessment Methods for Environmental Performance in the Circular Economy. Sustainable Prod. Consumption 2021, 26, 172–186. 10.1016/J.SPC.2020.09.018.

[ref8] European Commission. A European Strategy for Plastics in a Circular Economy - COM(2018) 28; Brussels, 2018.

[ref9] Antonopoulos I.; Faraca G.; Tonini D. Recycling of Post-Consumer Plastic Packaging Waste in EU: Process Efficiencies, Material Flows, and Barriers. Waste Manage. 2021, 126, 694–705. 10.1016/j.wasman.2021.04.002. PMC8162419 33887695

[ref10] Cimpan C.; Bjelle E. L.; Strømman A. H. Plastic Packaging Flows in Europe: A Hybrid Input-output Approach. J. Ind. Ecol. 2021, 25, 1572–1587. 10.1111/jiec.13175.

[ref11] Di J.; Reck B. K.; Miatto A.; Graedel T. E. United States Plastics: Large Flows, Short Lifetimes, and Negligible Recycling. Resour., Conserv. Recycl. 2021, 167, 105440 10.1016/j.resconrec.2021.105440.

[ref12] Hsu W.-T.; Domenech T.; McDowall W. How Circular Are Plastics in the EU?: MFA of Plastics in the EU and Pathways to Circularity. Cleaner Environ. Syst. 2021, 2, 100004 10.1016/j.cesys.2020.100004.

[ref13] Kawecki D.; Scheeder P. R. W.; Nowack B. Probabilistic Material Flow Analysis of Seven Commodity Plastics in Europe. Environ. Sci. Technol. 2018, 52, 9874–9888. 10.1021/acs.est.8b01513. 30004221

[ref14] Eriksen M. K.; Pivnenko K.; Faraca G.; Boldrin A.; Astrup T. F. Dynamic Material Flow Analysis of PET, PE, and PP Flows in Europe: Evaluation of the Potential for Circular Economy. Environ. Sci. Technol. 2020, 54, 16166–16175. 10.1021/acs.est.0c03435. 33225689

[ref15] Lau W. W. Y.; Shiran Y.; Bailey R. M.; Cook E.; Stuchtey M. R.; Koskella J.; Velis C. A.; Godfrey L.; Boucher J.; Murphy M. B.; Thompson R. C.; Jankowska E.; Castillo Castillo A.; Pilditch T. D.; Dixon B.; Koerselman L.; Kosior E.; Favoino E.; Gutberlet J.; Baulch S.; Atreya M. E.; Fischer D.; He K. K.; Petit M. M.; Sumaila U. R.; Neil E.; Bernhofen M. V.; Lawrence K.; Palardy J. E. Evaluating Scenarios toward Zero Plastic Pollution. Science 2020, 369, 1455–1461. 10.1126/science.aba9475. 32703909

[ref16] Zheng J.; Suh S. Strategies to Reduce the Global Carbon Footprint of Plastics. Nat. Clim. Change 2019, 9, 374–378. 10.1038/s41558-019-0459-z.

[ref17] Meys R.; Kätelhön A.; Bachmann M.; Winter B.; Zibunas C.; Suh S.; Bardow A. Achieving Net-Zero Greenhouse Gas Emission Plastics by a Circular Carbon Economy. Science 2021, 374, 71–76. 10.1126/SCIENCE.ABG9853. 34591623

[ref18] Chu J.; Zhou Y.; Cai Y.; Wang X.; Li C.; Liu Q. Life-Cycle Greenhouse Gas Emissions and the Associated Carbon-Peak Strategies for PS, PVC, and ABS Plastics in China. Resour., Conserv. Recycl. 2022, 182, 106295 10.1016/j.resconrec.2022.106295.

[ref19] Chaudhari U. S.; Johnson A. T.; Reck B. K.; Handler R. M.; Thompson V. S.; Hartley D. S.; Young W.; Watkins D.; Shonnard D. Material Flow Analysis and Life Cycle Assessment of Polyethylene Terephthalate and Polyolefin Plastics Supply Chains in the United States. ACS Sustainable Chem. Eng. 2022, 10, 13145–13155. 10.1021/ACSSUSCHEMENG.2C04004.

[ref20] Basuhi R.; Moore E.; Gregory J.; Kirchain R.; Gesing A.; Olivetti E. A. Environmental and Economic Implications of U.S. Postconsumer Plastic Waste Management. Resour., Conserv. Recycl. 2021, 167, 105391 10.1016/J.RESCONREC.2020.105391.

[ref21] Tallentire C. W.; Steubing B. The Environmental Benefits of Improving Packaging Waste Collection in Europe. Waste Manage. 2020, 103, 426–436. 10.1016/j.wasman.2019.12.045. 31952024

[ref22] Tenhunen-Lunkka A.; Rommens T.; Vanderreydt I.; Mortensen L. Greenhouse Gas Emission Reduction Potential of European Union’s Circularity Related Targets for Plastics. Circ. Econ. Sustainability 2022, 3, 475–510. 10.1007/s43615-022-00192-8. PMC9282144 35855295

[ref23] Hestin M.; Faninger T.; Milios L. Increased EU Plastics Recycling Targets: Environmental, Economic and Social Impact Assessment - Final Report; Brussels, 2015.

[ref24] Bassi S. A.; Tonini D.; Saveyn H.; Astrup T. F. Environmental and Socioeconomic Impacts of Poly(Ethylene Terephthalate) (PET) Packaging Management Strategies in the EU. Environ. Sci. Technol. 2022, 56, 501–511. 10.1021/acs.est.1c00761. 34875164

[ref25] Ferrão P.; Ribeiro P.; Rodrigues J.; Marques A.; Preto M.; Amaral M.; Domingos T.; Lopes A.; Costa I. Environmental, Economic and Social Costs and Benefits of a Packaging Waste Management System: A Portuguese Case Study. Resour., Conserv. Recycl. 2014, 85, 67–78. 10.1016/j.resconrec.2013.10.020.

[ref26] Suh S.; Huppes G. Methods for Life Cycle Inventory of a Product. J. Cleaner Prod. 2005, 13, 687–697. 10.1016/j.jclepro.2003.04.001.

[ref27] Majeau-Bettez G.; Strømman A. H.; Hertwich E. G. Evaluation of Process- and Input–Output-Based Life Cycle Inventory Data with Regard to Truncation and Aggregation Issues. Environ. Sci. Technol. 2011, 45, 10170–10177. 10.1021/es201308x. 22060273

[ref28] Vercalsteren A.; Christis M.; Geerken T.; Van der Linden A. Policy Needs (to Be) Covered by Static Environmentally Extended Input–Output Analyses. Econ. Syst. Res. 2020, 32, 121–144. 10.1080/09535314.2019.1644994.

[ref29] Aguilar-Hernandez G. A.; Sigüenza-Sanchez C. P.; Donati F.; Rodrigues J. F. D.; Tukker A. Assessing Circularity Interventions: A Review of EEIOA-Based Studies. J. Econ. Struct. 2018, 7, 14 10.1186/s40008-018-0113-3.

[ref30] Wiebe K. S.; Norstebø V. S.; Aponte F. R.; Simas M. S.; Andersen T.; Perez-Valdes G. A. Circular Economy and the Triple Bottom Line in Norway. Circ. Econ. Sustainability 2022, 3, 1–33. 10.1007/s43615-021-00138-6.

[ref31] Cabernard L.; Pfister S.; Oberschelp C.; Hellweg S. Growing Environmental Footprint of Plastics Driven by Coal Combustion. Nat. Sustainability 2022, 5, 139–148. 10.1038/s41893-021-00807-2.

[ref32] Nakamura S.; Kondo Y. Input-Output Analysis of Waste Management. J. Ind. Ecol. 2002, 6, 39–63. 10.1162/108819802320971632.

[ref33] Nakamura S.; Nakajima K.; Kondo Y.; Nagasaka T. The Waste Input-Output Approach to Materials Flow Analysis: Concepts and Application to Base Metals. J. Ind. Ecol. 2007, 11, 50–63. 10.1162/jiec.2007.1290.

[ref34] Merciai S.; Schmidt J. Methodology for the Construction of Global Multi-Regional Hybrid Supply and Use Tables for the EXIOBASE v3 Database. J. Ind. Ecol. 2018, 22, 516–531. 10.1111/jiec.12713.

[ref35] Hawkins T.; Hendrickson C.; Higgins C.; Matthews H. S.; Suh S. A Mixed-Unit Input-Output Model for Environmental Life-Cycle Assessment and Material Flow Analysis. Environ. Sci. Technol. 2007, 41, 1024–1031. 10.1021/es060871u. 17328219

[ref36] Nakamura S.; Murakami S.; Nakajima K.; Nagasaka T. Hybrid Input–Output Approach to Metal Production and Its Application to the Introduction of Lead-Free Solders. Environ. Sci. Technol. 2008, 42, 3843–3848. 10.1021/es702647b. 18546732

[ref37] Beylot A.; Vaxelaire S.; Villeneuve J. Reducing Gaseous Emissions and Resource Consumption Embodied in French Final Demand: How Much Can Waste Policies Contribute?. J. Ind. Ecol. 2016, 20, 905–916. 10.1111/jiec.12318.

[ref38] Budzinski M.; Bezama A.; Thrän D. Estimating the Potentials for Reducing the Impacts on Climate Change by Increasing the Cascade Use and Extending the Lifetime of Wood Products in Germany. Resour., Conserv. Recycl.: X 2020, 6, 100034 10.1016/j.rcrx.2020.100034.

[ref39] Towa E.; Zeller V.; Achten W. M. J. Circular Economy Scenario Modelling Using a Multiregional Hybrid Input-Output Model: The Case of Belgium and Its Regions. Sustainable Prod. Consumption 2021, 27, 889–904. 10.1016/J.SPC.2021.02.012.

[ref40] Geerken T.; Schmidt J.; Boonen K.; Christis M.; Merciai S. Assessment of the Potential of a Circular Economy in Open Economies – Case of Belgium. J. Cleaner Prod. 2019, 227, 683–699. 10.1016/j.jclepro.2019.04.120.

[ref41] Lenzen M.; Reynolds C. J. A Supply-Use Approach to Waste Input-Output Analysis. J. Ind. Ecol. 2014, 18, 212–226. 10.1111/jiec.12105.

[ref42] Stadler K.; Wood R.; Bulavskaya T.; Södersten C. J.; Simas M.; Schmidt S.; Usubiaga A.; Acosta-Fernández J.; Kuenen J.; Bruckner M.; Giljum S.; Lutter S.; Merciai S.; Schmidt J. H.; Theurl M. C.; Plutzar C.; Kastner T.; Eisenmenger N.; Erb K. H.; de Koning A.; Tukker A. EXIOBASE 3: Developing a Time Series of Detailed Environmentally Extended Multi-Regional Input-Output Tables. J. Ind. Ecol. 2018, 22, 502–515. 10.1111/jiec.12715.

[ref43] Eurostat. EU Inter-Country Supply, Use and Input-Output Tables — Full International and Global Accounts for Research in Input-Output Analysis (FIGARO); Luxembourg, 2019.

[ref44] Algarin J. V.; Hawkins T. R.; Marriott J.; Matthews H. S.; Khanna V. Disaggregating the Power Generation Sector for Input-Output Life Cycle Assessment. J. Ind. Ecol. 2015, 19, 666–675. 10.1111/jiec.12207.

[ref45] Eurostat. Air Emissions Accounts by NACE Rev. 2 Activity (Env_ac_ainah_r2), Eurostat, the statistical office of the European Union, 2018.

[ref46] Eurostat. Employment by Sex, Age and Detailed Economic Activity (from 2008 Onwards, NACE Rev. 2 Two-Digit Level), Eurostat, the statistical office of the European Union, 2018.

[ref47] Leontief W. Environmental Repercussions and the Economic Structure: An Input-Output Approach. Rev. Econ. Stat. 1970, 52, 262–271. 10.2307/1926294.

[ref48] Lenzen M.; Rueda-Cantuche J. M. A Note on the Use of Supply-Use Tables in Impact Analyses. Stat. Oper. Res. Trans. 2012, 36, 139–152.

[ref49] Wood R.; Moran D.; Stadler K.; Ivanova D.; Steen-Olsen K.; Tisserant A.; Hertwich E. G. Prioritizing Consumption-Based Carbon Policy Based on the Evaluation of Mitigation Potential Using Input-Output Methods. J. Ind. Ecol. 2018, 22, 540–552. 10.1111/jiec.12702.

[ref50] Wiebe K. S.; Bjelle E. L.; Többen J.; Wood R. Implementing Exogenous Scenarios in a Global MRIO Model for the Estimation of Future Environmental Footprints. J. Econ. Struct 2018, 7, 20 10.1186/s40008-018-0118-y.

[ref51] European Commission. Guidance for the Compilation and Reporting of Data on Packaging and Packaging Waste According to Decision 2005/270/EC, Brussels, Belgium, 2020.

[ref52] PlasticsEurope. Plastics - the Facts 2019. An Analysis of European Plastics Production, Demand and Waste Data; Brussels, 2019.

[ref53] Conversio. Final Report - Circular Economy of Plastics 2018 EU28 + 2, Brussels, Belgium, 2019.

[ref54] Nakatani J.; Maruyama T.; Moriguchi Y. Revealing the Intersectoral Material Flow of Plastic Containers and Packaging in Japan. Proc. Natl. Acad. Sci. U.S.A. 2020, 117, 19844–19853. 10.1073/pnas.2001379117. 32747531 PMC7443912

[ref55] US Input-Output Accounts Data, Bureau of Economic Analysis.

[ref56] Eunomia. PET Market in Europe - State of Play: Production, Collection and Recycling Data. Brussels, 2020.

[ref57] Eunomia. Flexible Films Market in Europe - State of Play: Production, Collection and Recycling Data. Brussels, 2020.

[ref58] Eunomia. HDPE & PP Market in Europe - State of Play: Production, Collection and Recycling Data. Brussels, 2020.

[ref59] Blueprint for Plastics Packaging Waste: Quality Sorting & Recycling - Final Report, 2017.

[ref60] Packaging Waste by Waste Operations and Waste Flow [Env_waspac]; Statistical Office of the European Union: Luxembourg.

[ref61] Hogg D.; Elliott T.; Corbin M.; Hilton M.; Tsiarta C.; Hudson J.; Vives R.; Sastre S.; Campos L.; Puig I.; Šleinotaitė-Budrienė L.; Lippa M.; Kazlauskaitė L. Study on Waste Statistics - A Comprehensive Review of Gaps and Weaknesses and Key Priority Areas for Improvement in the EU Waste Statistics. Brussels, 2017.

[ref62] The 2021 Ageing Report. Economic & Budgetary Projections for the EU Member States (2019-2070); Publications Office of the European Union: Luxembourg, 2021.

[ref63] Bjelle E. L.; Wiebe K. S.; Többen J.; Tisserant A.; Ivanova D.; Vita G.; Wood R. Future Changes in Consumption: The Income Effect on Greenhouse Gas Emissions. Energy Econ. 2021, 95, 105114 10.1016/j.eneco.2021.105114.

[ref64] European Commission. European Economic Forecast Autumn 2021, 2021.

[ref65] Scott K.; Giesekam J.; Barrett J.; Owen A. Bridging the Climate Mitigation Gap with Economy-Wide Material Productivity. J. Ind. Ecol. 2019, 23, 918–931. 10.1111/jiec.12831. PMC13065607 41970938

[ref66] O’Neill B. C.; Kriegler E.; Ebi K. L.; Kemp-Benedict E.; Riahi K.; Rothman D. S.; van Ruijven B. J.; van Vuuren D. P.; Birkmann J.; Kok K.; Levy M.; Solecki W. The Roads Ahead: Narratives for Shared Socioeconomic Pathways Describing World Futures in the 21st Century. Global Environ. Change 2017, 42, 169–180. 10.1016/j.gloenvcha.2015.01.004.

[ref67] Gibon T.; Wood R.; Arvesen A.; Bergesen J. D.; Suh S.; Hertwich E. G. A Methodology for Integrated, Multiregional Life Cycle Assessment Scenarios under Large-Scale Technological Change. Environ. Sci. Technol. 2015, 49, 11218–11226. 10.1021/acs.est.5b01558. 26308384

[ref68] EU Reference Scenario 2016 - Energy, Transport and GHG Emissions Trends to 2050; Publications Office of the European Union: Luxembourg, 2016.

[ref69] Vrontisi Z.; Fragkiadakis K.; Kannavou M.; Capros P. Energy System Transition and Macroeconomic Impacts of a European Decarbonization Action towards a below 2 °C Climate Stabilization. Clim. Change 2020, 162, 1857–1875. 10.1007/s10584-019-02440-7.

[ref70] European Commission. Effectiveness of the Essential Requirements for Packaging and Packaging Waste and Proposals for Reinforcement, 2020.

[ref71] Directive (EU) 2019/904 of the European Parliament and of the Council of 5 June 2019 on the Reduction of the Impact of Certain Plastic Products on the Environment; Official Journal of the European Union, 2019.

[ref72] Directive (EU) 2015/720 of the European Parliament and of the Council of 29 April 2015 Amending Directive 94/62/EC as Regards Reducing the Consumption of Lightweight Plastic Carrier Bags; Official Journal of the European Union, 2015.

[ref73] Directive 94/62/EC European Parliament and Council Directive 94/62/EC of 20 December 1994 on Packaging and Packaging Waste, 1994.

[ref74] PlasticsEurope. PlasticsEurope’s Position on Recycled Content for Plastics Packaging under the Review of the Directive 94/62/EC on Packaging and Packaging Waste (PPWD). Brussels, 2021.

[ref75] Directive (EU) 2018/850 of the European Parliament and of the Council of 30 May 2018 Amending Directive 1999/31/EC on the Landfill of Waste; Official Journal of the European Union, 2018.

[ref76] Brouwer M. T.; van Velzen E. U. T.; Ragaert K.; Klooster R. t. Technical Limits in Circularity for Plastic Packages. Sustainability 2020, 12, 10021 10.3390/su122310021.

[ref77] Eriksen M. K.; Christiansen J. D.; Daugaard A. E.; Astrup T. F. Closing the Loop for PET, PE and PP Waste from Households: Influence of Material Properties and Product Design for Plastic Recycling. Waste Manage. 2019, 96, 75–85. 10.1016/j.wasman.2019.07.005. 31376972

[ref78] Tonini D.; Garcia-gutierrez P.; Nessi S. Environmental Effects of Plastic Waste Recycling. Luxembourg, 2021.

[ref79] Lee P.; Sims E.; Bertham O.; Symington H.; Bell N.; Pfaltzgraff L.; Sjögren P.; Wilts H.; O’Brien M. Towards a Circular Economy – Waste Management in the EU. Brussels, 2017.

[ref80] Bjelle E. L.; Többen J.; Stadler K.; Kastner T.; Theurl M. C.; Erb K.-H.; Olsen K.-S.; Wiebe K. S.; Wood R. Adding Country Resolution to EXIOBASE: Impacts on Land Use Embodied in Trade. J. Econ. Struct. 2020, 9, 14 10.1186/S40008-020-0182-Y. 32117682 PMC7021151

[ref81] Tisserant A.; Pauliuk S.; Merciai S.; Schmidt J.; Fry J.; Wood R.; Tukker A. Solid Waste and the Circular Economy: A Global Analysis of Waste Treatment and Waste Footprints. J. Ind. Ecol. 2017, 21, 628–640. 10.1111/jiec.12562.

[ref82] Wang C.; Liu Y.; Chen W.; Zhu B.; Qu S.; Xu M. Critical Review of Global Plastics Stock and Flow Data. J. Ind. Ecol. 2021, 25, 1300–1317. 10.1111/jiec.13125.

[ref83] Eurostat. Creating Consolidated and Aggregated EU27 Supply, Use and Input-Output Tables, Adding Environmental Extensions (Air Emissions), and Conducting Leontief-Type Modelling to Approximate Carbon and Other “footprints” of EU27 Consumption for 2000 to 2006. Luxembourg, 2011.

[ref84] Steubing B.; de Koning A.; Merciai S.; Tukker A. How Do Carbon Footprints from LCA and EEIOA Databases Compare?: A Comparison of Ecoinvent and EXIOBASE. J. Ind. Ecol. 2022, 26, 1406–1422. 10.1111/JIEC.13271.

[ref85] de Koning A.; Bruckner M.; Lutter S.; Wood R.; Stadler K.; Tukker A. Effect of Aggregation and Disaggregation on Embodied Material Use of Products in Input-Output Analysis. Ecol. Econ. 2015, 116, 289–299. 10.1016/j.ecolecon.2015.05.008.

[ref86] Winning M.; Calzadilla A.; Bleischwitz R.; Nechifor V. Towards a Circular Economy: Insights Based on the Development of the Global ENGAGE-Materials Model and Evidence for the Iron and Steel Industry. Int. Econ. Econ. Policy 2017, 14, 383–407. 10.1007/s10368-017-0385-3.

[ref87] Zink T.; Geyer R. Circular Economy Rebound. J. Ind. Ecol. 2017, 21, 593–602. 10.1111/jiec.12545.

[ref88] Vivanco D. F.; Freire-González J.; Kemp R.; van der Voet E. The Remarkable Environmental Rebound Effect of Electric Cars: A Microeconomic Approach. Environ. Sci. Technol. 2014, 48, 12063–12072. 10.1021/es5038063. 25260014

[ref89] Bisinella V.; Hulgaard T.; Riber C.; Damgaard A.; Christensen T. H. Environmental Assessment of Carbon Capture and Storage (CCS) as a Post-Treatment Technology in Waste Incineration. Waste Manage. 2021, 128, 99–113. 10.1016/j.wasman.2021.04.046. 33975140

[ref90] Hahladakis J. N.; Iacovidou E. Closing the Loop on Plastic Packaging Materials: What Is Quality and How Does It Affect Their Circularity?. Sci. Total Environ. 2018, 630, 1394–1400. 10.1016/j.scitotenv.2018.02.330. 29554759

[ref91] Demets R.; Van Kets K.; Huysveld S.; Dewulf J.; De Meester S.; Ragaert K. Addressing the Complex Challenge of Understanding and Quantifying Substitutability for Recycled Plastics. Resour., Conserv. Recycl. 2021, 174, 105826 10.1016/j.resconrec.2021.105826.

[ref92] Rigamonti L.; Taelman S. E.; Huysveld S.; Sfez S.; Ragaert K.; Dewulf J. A Step Forward in Quantifying the Substitutability of Secondary Materials in Waste Management Life Cycle Assessment Studies. Waste Manage. 2020, 114, 331–340. 10.1016/j.wasman.2020.07.015. 32688065

[ref93] Crippa M.; De Wilde B.; Koopmans R.; Leyssens J.; Muncke J.; A-C R.; Van Doorsselaer K.; Velis C.; Wagner M. A Circular Economy for Plastics – Insights from Research and Innovation to Inform Policy and Funding Decisions; De Smet M.; Linder M., Eds.; Publications Office of the European Union: Brussels, Belgium, 2019.

[ref94] Cimpan C.; Maul A.; Jansen M.; Pretz T.; Wenzel H. Central Sorting and Recovery of MSW Recyclable Materials: A Review of Technological State-of-the-Art, Cases, Practice and Implications for Materials Recycling. J. Environ. Manage. 2015, 156, 181–199. 10.1016/j.jenvman.2015.03.025. 25845999

[ref95] Ebner N.; Iacovidou E. The Challenges of Covid-19 Pandemic on Improving Plastic Waste Recycling Rates. Sustainable Prod. Consumption 2021, 28, 726–735. 10.1016/j.spc.2021.07.001. PMC8536949 34722849

