# Supplementary material for: Effects of Circularity Interventions in the European Plastic Packaging Sector
Source: Environ Sci Technol. 2023 Jun 29;57(27):9984–95. doi: 10.1021/acs.est.2c08202 (PMC10339715; doi:10.1021/acs.est.2c08202)
Supplement: Supplementary file 1 — es2c08202_si_001.pdf [file es2c08202_si_001.pdf]

Supporting Information (A) for:

Effects of Circularity Interventions in the European Plastic Packaging Sector

Ciprian Cimpan[1], Eivind Lekve Bjelle[2], Maik Budzinski[1], Richard Wood[1], Anders Hammer Strømman[1]*

[1]Industrial Ecology Programme, Department of energy and process engineering, Norwegian University of Science and Technology (NTNU), Trondheim, Norway

[2]Mobility and Economics, SINTEF Community, Trondheim, Norway

*Corresponding author: Ciprian Cimpan, e-mail: cimpan.ciprian@gmail.com, cic@igt.sdu.dk, tel.: +45 2440 9882

Overview:

- Number of pages: 47
- Number of tables: 15
- Number of figures: 29

Table of content

1 Mass flow data for plastic packaging in EU-28, base year 2018 3
1.1 Production stages 4
1.2 Consumption of packaging 5
1.3 Waste generation and management 7
1.3.1 Waste streams 7
1.3.2 Management of pre- and post-consumer waste captured for recycling 8
2 Table disaggregation and model construction 10
2.1 Overall approach 10
2.2 Disaggregation procedures 12
2.2.1 Disaggregation in the background 12
2.2.2 Disaggregation of foreground upstream 14
2.2.3 Disaggregation of foreground downstream 16
2.2.4 Disaggregation of extensions 20
2.3 Base model 21
3 Projecting the model to 2030 and scenarios implementation 23
3.1 Projection of the background frame 23
3.2 Foreground scenario narratives and circularity interventions 29
3.3. Scenario model runs 34
4 Additional result figures 35
References 44

List of abbreviations

CE	Circular Economy
kt	kilotons, where ton should always be understood as metric ton = 1000 kg
EU-28+2	EU-28 is EU-27(from 2020) + United Kingdom; when EU is used it denotes EU-28; +2 denotes Norway and Switzerland
RoW	Rest of the world region
PE-LD	Polyethylene low-density
PE-HD	Polyethylene high-density
PET	Polyethylene terephthalate
PP	Polypropylene
PS	Polystyrene
PS-E	Polystyrene expanded
PVC	Polyvinyl chloride
IO-MFA	Input-Output based Material Flow Analysis
IOT	Input-Output table
SUT	Supply-Use tables
WIO	Waste Input-Output, see Nakamura and Kondo[1]
WSUT	Waste Supply-Use tables, see Lenzen and Reynolds[2]
LCI data	Life cycle inventory data
GDP	Gross Domestic Product
SUP	Single use plastics (referred in general in connection to the EU Directive)
WtE	Waste-to-energy, denoting thermal (incineration) waste treatment

1 Mass flow data for plastic packaging in EU-28, base year 2018

This section details the data used to construct the foreground upstream (production stages), the consumption stage (interindustry and industry to final demand flows), and foreground downstream (waste management stages). The overall network of material flows captured in the model is illustrated in Figure S1 below.

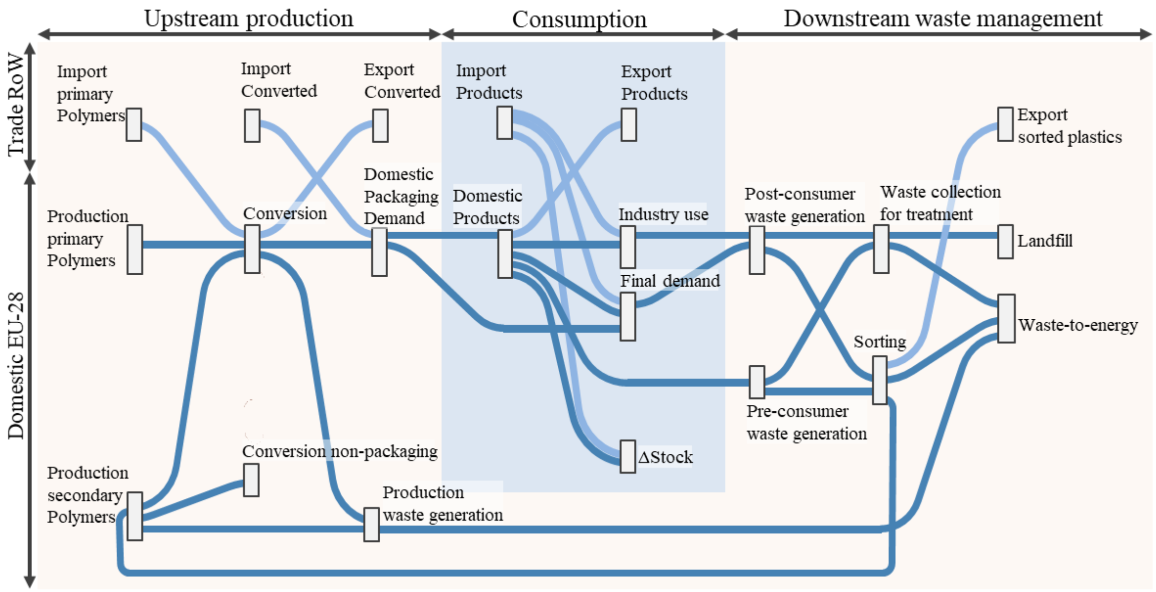

Figure S1: Structure of the material flows of plastics in the model, distinguishing sections of upstream production, consumption, and waste management. The light blue rectangle highlights the material flows which are determined with use of monetary flow data (WIO-MFA).

Plastic packaging flows for the reference year 2018 are illustrated in Figure S2.

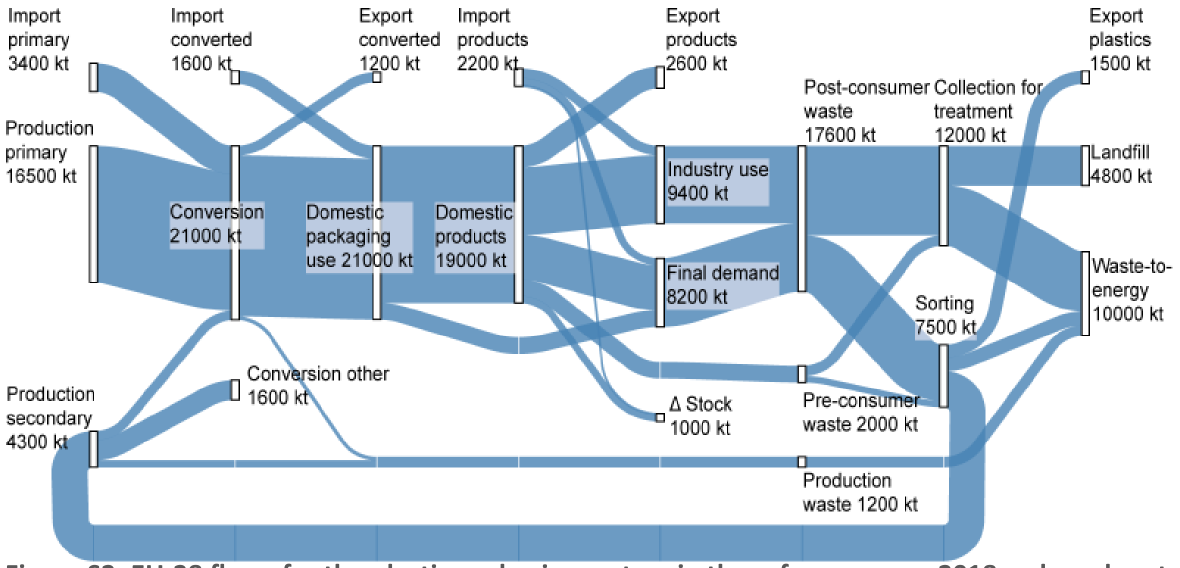

Figure S2: EU-28 flows for the plastic packaging system in the reference year 2018, values denote process totals and are rounded to two/three significant digits.

1.1 Production stages

Production stages include primary plastic production, secondary plastic production (i.e., recycling, or production of recyclate from recovered plastics), conversion of plastics to packaging and conversion to plastic products for other applications.

The starting point for the material flows mapping was the 2018 EU28+2 converter demand for polymers in primary forms documented by Plastics Europe, the Association for Plastics Manufacturers in Europe. For production of packaging, converter demand was 20,420 kt, with the polymer distribution presented in Table S1[3]. The total amount was reduced by 550 kt, accounting for the removal of Switzerland and Norway[4,5] and maintaining the overall polymer distribution.

Table S1: Converter demand by polymer in Europe in 2018. All values in kt.

	PE-LD	PE-HD	PP	PS	PS-E	PVC	PET	Other	Total
**EU-28+2** Total demand	8980	6240	9860	1800	1510	5110	3930	13750	51180
**EU-28+2** Demand packaging	6150	3630	4580	850	300	450	3890	570	20420
**EU-28** Demand packaging	5984	3532	4457	827	292	438	3785	555	19870

It was then assumed that the EU-28 converter demand for packaging production was met by primary polymers produced domestically and a share that was imported. The shares of total plastics imports assigned to packaging were assumed to be proportional to the share of packaging in the total plastic converter demand in EU-28 (Table S2). The real use share of plastic imports to packaging conversion is not known. Trade statistics for 2018 were retrieved from the UN Comtrade database[6]. The domestic contribution was calculated as the difference between total converter demand and imports.

Table S2: Share of polymer used in packaging and calculated import values of primary plastics for packaging conversion (kt)

	PE-LD	PE-HD	PP	PS	PS-E	PVC	PET	Other	Total
**% packaging**	68%	58%	46%	47%	20%	9%	98%	n.i.	
**EU-28 Import**	988	900	509	53	17	56	882	n.i.	3405

n.i. - not included

Packaging converter demand reported by Plastics Europe does not include the input of secondary/recycled polymers. The total contribution of secondary plastics to packaging conversion was estimated to 1,100 kt total and polymer specific values are shown in Table S3. The underlying calculation approach to secondary polymers production and use in the packaging sector is presented in section 1.3, which covers waste generation and treatment.

The conversion process generates plastic scrap accounting to 7% of input polymers. However, a majority of this production waste is recovered and recycled internally in the industry[7,8]. Therefore, we accounted a total conversion yield at around 98%, after internal recycling. This rate was assumed uniform for all plastic polymers. The inputs and outputs of the conversion process are given in Table S3.

Table S3: Conversion process mass balance including internal recycling. All values in kt.

	PE-LD	PE-HD	PP	PS	PS-E	PVC	PET	Other	Total
**Inputs**									
Primary plastics	5984	3532	4457	827	292	438	3785	555	19870
Secondary plastics	180	120	50	0	0	0	750	0	1100

Outputs									
Final conversion waste	123	73	90	17	6	9	91	11	419
Converted packaging	6041	3579	4417	810	286	429	4444	544	20551

Trade with converted packaging was retrieved from Comtrade and polymer composition was associated to commodities based on the work of Kawecki et al.[9].

Table S4: Trade with converted packaging in 2018. All values in kt.

	PE-LD	PE-HD	PP	PS	PS-E	PVC	PET	Other	Total
**EU-28 Import**	578	188	268	21	3	31	412	67	1568
**EU-28 Export**	214	204	355	32	3	29	240	104	1181

Table S5: Polymer composition for traded converted packaging (from Kawecki et al., 2018)

Trade Code	PE-LD	PE-HD	PP	PS	PS-E	PVC	PET	Other
392310	3%	12%	48%	7%	0	3%	0	27%
392321	88%	12%	0	0	0	0	0	0
392329	0	0	79%	0	0	16%	0	5%
392330	0	34%	0	0	0	3%	63%	0
392340	3%	12%	48%	7%	0	3%	0	27%
392350	7%	3%	61%	0	0	0	0	2%
392390	32%	18%	21%	5%	2%	2%	17%	3%

The total converted packaging entering use in the EU, including to industry which will use it to pack or transport products, and direct use by final demand, is then the sum of domestic conversion output (Table S3) and imported converted packaging, with exported converted packaging being subtracted (Table S4). Accordingly, the estimate for total converted packaging entering use in the EU in 2018 was 20,938 kt.

1.2 Consumption of packaging

The consumption stage of packaging and waste generation within the EU-28 economy were described using the IO-MFA approach[10,11], i.e. intersectoral and sector-final demand monetary flows serve as vehicle for estimation of packaging flows, under the assumption that a certain amount (content) of packaging is associated with goods and services produced and used throughout the economy. For a representation of the EU-28 economy in 2018, we used a framework of supply and use tables (SUTs) as elaborated in Section 2 of this SI. The sectoral representation of packaging consumption and generation of packaging waste is described in detail as part of the table construction procedures in Section 2 of this SI.

In a first stage, converted packaging is purchased by different sectors in the economy and used to either package goods or in the process of delivering services (e.g., sack and bags in industrial laundries). We do not denote converted packaging as empty packaging and packaging attached to goods and services as filled packaging, as these terms do not always apply (e.g., packaging expended internally to deliver a service). Pre-consumer packaging waste occurs at this stage within the sectors consuming converted packaging. Forward, packaged goods (and services) are purchased and consumed by various industry, service sectors and by final demand categories (largest being households). Certain amounts of packaging are also purchased directly by final demand (e.g., shopping carrier bags), without being associated to a specific product. In this final stage of consumption, packaging is discarded and becomes post-consumer packaging waste. Inflows of packaged products in

EU-28 imports, as well as the outflows with exported products are accounted. For consistency, the eventual pre-consumer waste pertaining to imported products was not accounted as inflow to EU (as it occurs in the export country), while the equivalent pre-consumer waste pertaining to products that are exported extra-EU was accounted as waste generated domestically.

We accounted for the possible delay between consumption of packaging and generation of packaging waste in a relatively simple way, by considering a net addition to stock. This is computed between the two stages of consumption, i.e., after accounting pre-consumer waste, and before final consumption. This approach is opposite to most MFA studies available which in the case of packaging either: (1) calculate the net addition to stock as the difference between packaging put on market (POM) and generated packaging waste, using the packaging and waste statistics[12], and (2) do not include stocks for packaging, especially country level studies, due to assumed service life of less than one year[13,14].

Data reported to Eurostat and also used by Plastics Europe, reflect a large gap of around 15% between packaging consumption and waste generation, which is assumed to be addition to stock. However, this is not supported by the relative stable consumption in the EU. Other aspects, such as poorly accounted packaging with import/exports and growth in reusable (transport) packaging, may explain part of it. The likely course for a large part of this gap is the data collection process. Issues with reporting of packaging consumption and waste statistics have been found to be extensive, and were reported at EU level[15,16], and in various member states, e.g., former member UK[17], Spain[18], and the Netherlands[19].

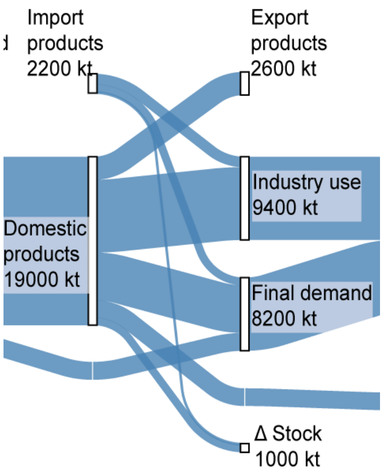

The approach used in this work was to first determine total consumption (domestic products + direct final demand + import products – export products), calculate potential for additions to stocks, and finally determine waste output as total consumption – addition to stocks. The calculation of pre-consumer and post-consumer waste is described in Section 1.3.

Figure S3: Closeup of packaging flows in consumption

Stock rates (additions to stock)

Packaging production and use in Europe in not in a steady state, but defined by a relative growth at a yearly rate of around 2%[20,21]. This growth leads: (1) to an increase in the in-use stock of longer-lived packaging (e.g., certain transport packaging), (2) can lead to differences between production and waste generation of sales packaging if not all packaged products are sold and consumed in the same year. Thus, waste generation is a sum of packaging produced in 2018 and fractions of packaging from previous years, which are discarded in 2018.

In this work, net stock additions were assumed to represent around 5% of the total packaging that entered consumption in 2018. Accordingly, around 1000 kt of contributed to net addition to stocks. A more elaborate description of packaging stocks is presented in Cimpan et al.[22].

1.3 Waste generation and management

It is important to state that the mass values for waste streams did not include non-plastic contamination or moisture, and therefore refer to pure plastic content. Waste streams were determined considering the use of converted packaging within the EU as well as the inflow of packaging with imported products to the EU from RoW.

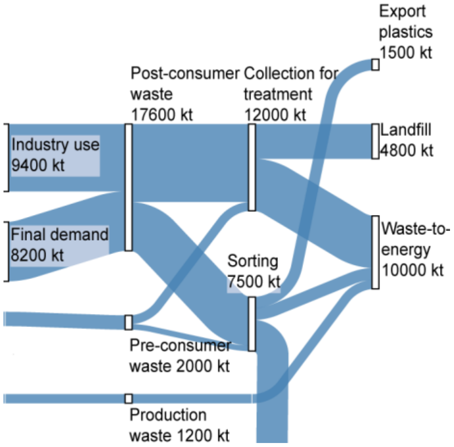

In this work we accounted waste arising at different points in the packaging lifecycle, namely: (1) production waste (recycling residues/rejects and conversion waste), (2) pre-consumer waste (packing/ manufacturing waste, transport/ wholesale and retail waste, and service waste), and (3) post-consumer waste (from industrial and final demand sectors).

Figure S4: Closeup of packaging waste flows

1.3.1 Waste streams

(1) Production waste consists of final waste from conversion after internal recycling is accounted (Table S3, 419 kt) and residues from production of secondary plastics. The latter is the difference between EU input to reprocessing (mechanical recycling) and its output, values (given in Table S6, 827 kt). Regarding treatment, it was assumed that all production waste was treated by waste-to-energy (WtE) processes, due to the relative high energy content of plastic residues and the industrial setting where this waste occurs.

(2) Pre-consumer waste is a share of converted packaging that is purchased by sectors and will be discarded within the sector, thus not reaching the final consumer. It consists of waste arising during product packing and later, waste arising due to damage or expiration of products during transport, storage, and retail. Pre-consumer waste can also be accounted as packaging discarded in the process of delivering services. Pre-consumer waste was computed with product specific loss rates (% of used converted packaging), covering packing, commerce, and service delivery, and are given in the associated SI B excel files.

Losses of packaging during packing processes can be estimated based on the measure of quality or efficiency in such processes, which is calculated typically as the rate of product output meeting specifications to total product output[23]. In most cases products out of spec (packing failure) would be discarded. Food loss due to packing operations was previously estimated around 0.5%[24]. This figure was used as a proxy for losses of packaging during food products manufacturing. For products other than food and beverages we adopted a rate of 0.1%. This is reasonable considering the robustness of other products and the general very high efficiency of industrial packing operations.

Packaging waste arises also during transport and storage associated with wholesale and retail operations. Product specific rates were associated from the JRC report by Nessi et al.[25] for specific products, while a generic rate of 1% was assumed for other manufactured products not covered by this report. Lastly, to account for service-related packaging waste, we adopted a 50% discard rate in the sectors providing services. We therefore assumed

that the remaining 50% of packaging was discarded in the sector or final demand category where the service was used.

Following the calculation procedure, 1969 kt of the total 20,938 kt of converted packaging used in the EU, became pre-consumer waste generated in 2018.

(3) Post-consumer packaging waste constitutes the sum of packaging discarded after direct use by final demand (i.e., mainly households) and packaging discarded with the domestic consumption of domestic produced products and imported products. The management of packaging waste with exported products was not included.

Post-consumer waste was determined with the IO-MFA approach[10] resulting in estimations of waste generated per industry sector and categories of final demand. Subsequently, due to a lack of source-specific (sector/final demand) data on waste management, the same ratios allocating waste towards recycling, WtE and landfilling were applied to both industry and household packaging waste.

The overall approach was to first map the management of quantities of post-consumer packaging waste which was captured by separate collection systems for recycling, for which information sources are available. The remaining waste quantities, that were not captured by collection for recycling, were attributed to generic mixed or residual waste collection and subsequent treatment. A simple distribution to either landfilling (40%) or waste-to-energy by incineration (60%) was then applied based on the distribution indicated by Eurostat[26]. The distribution was applied homogeneously to all polymers and packaging types.

1.3.2 Management of pre- and post-consumer waste captured for recycling

Polymer-specific flows within collection, sorting and reprocessing stages (last step in the recycling chain) were modelled based on data in several recent reports commissioned by Plastics Recyclers Europe[27–29], a study commissioned by Plastics Europe which mapped plastic flows for 2018[8], and the work by Deloitte which addressed plastic packaging for EU-28 in 2014[30].

We started with data on output from the final recycling process (the reprocessing step), which most clearly refer to mass of pure plastic content compared to collection and sorting. We then applied reprocessing, sorting, and collection efficiencies consistent with Deloitte (2017)[30] and Hestin et al. (2015)[21] to calculate back to quantities of plastics captured for recycling by collection systems. Reprocessing, or production of secondary plastics, was assumed to occur almost entirely through mechanical recycling processes.

According to Conversio[8], around 4,000 kt of secondary plastics (not accounting direct recycling of production scrap) were in total produced and used in EU-28 in 2018. This figure accounted for secondary plastics originating from waste from all sectors, not only packaging. The figure fits reasonably well with estimates from the three reports commissioned by Plastics Recyclers Europe, which reported around 1,350 kt of secondary PET[27], 1,200 kt of flexible PE[28], 700 kt of PE-HD, and 500 kt of PP[29]. The reports indicated that flexible PP, PET and other films were collected but largely not sorted for recycling. The amount of flexible PE-HD and PS plastics recycled were not investigated in this report series and some separate sourced were used.

There is no data that indicates how much of the 4,000 kt of total secondary plastics originated from packaging waste. An estimate could be made by using the shares of the

different polymers that were used in packaging applications and information in the above-mentioned reports. In addition, looking at total quantities diverted towards recycling (including production scrap), Conversio estimated that 7,500 kt pertained to packaging and 2,000 kt to all other applications. Considering the ratio between these two quantities and Plastics Recyclers Europe reports, we estimated that 80% to 85% of total secondary plastics pertained to packaging. We took forward the figure of 3,500 kt of the total secondary plastics originated in packaging. This is reasonable, as the collection and subsequent recovery of the plastics from applications outside of packaging generally display lower efficiencies than for packaging[9].

Information on PS-E packaging waste generation and management was added from EUMEPS (2018)[31], specifically a collection for recycling rate of 30-35%. Further, it was assumed that 10% of PS and PVC packaging was captured by separate collection for recycling, and that 50% of the collected PS was sorted for recycling. Reprocessing efficiency was assumed 80% for PS and PS-E. Lastly, it was assumed that PVC and Other polymers were not sent to recycling, despite small amounts being captured in separate collection. This is in line with knowledge about sorting operations for packaging waste which in a vast majority exclude polymers other that PE, PP and PET [32,33].

A share of plastics collected and sorted in the EU were then exported for recycling outside the EU, respectively around 1850 kt in 2018 (originating in packaging and other sources). Exports of packaging represent approx. 80% of plastic waste export[34]. Trade statistics identify PE, PP, PVC, and PS, and place all other polymers in a common code, which represented a total of 673 kt. We assumed that the largest share was accounted by PET.

Table S6: Amounts of packaging waste collected sorted and recycled. All values in kt.

	PE-LD	PE-HD	PP	PS	PS-E	PVC	PET	Other	Total
**Collected for recycling**	2412	1392	951	103	94	50	2457	50	7509
**Sorted total output**	1809	1183	571	51	84	0	2163	0	5861
**Sorted to export**	459	350	77	20	28	0	600	0	1534
**EU recycling input**	1350	833	494	31	56	0	1563	0	4327
**EU recycling output**	1080	700	400	25	45	0	1250	0	3500

Table S7: Sorting and recycling efficiencies, values in %.

	PE-LD	PE-HD	PP	PS	PS-E	PVC	PET	Other	Total
**Sorting efficiency**	75%	85%	60%	50%	90%	0%	88%	0%	78%
**Recycling efficiency**	80%	84%	80%	80%	80%	0%	80%	0%	81%

Conversio (2019) estimated total input of secondary plastics back into packaging conversion at around 1,000 kt. The Plastics Recyclers Europe reports would indicate this figure to be larger. We settled for 1,100 kt in order to remain consistent with the estimate used by Plastics Europe. We consider secondary input to packaging conversion to originate only in previous packaging as there is no data to indicate otherwise and considering the differences and quality, properties, and composition of plastics from other sectors.

Table S8: Uptake of recovered polymers (recyclate) into packaging conversion, values in kt.

Sector	PE-LD	PE-HD	PP	PS	PS-E	PVC	PET	Other	Total
**Packaging**	180	120	50	0	0	0	750	0	1100
**Other applications**	900	580	350	25	45	0	500	0	2400

2 Table disaggregation and model construction

The base tables before disaggregation:

- SUTs for 2018 EU-28 aggregated, in basic prices (unit of MEUR current prices), from Eurostat (Supply, use and input-output tables - ESA 2010 - current prices [naio_10_cp]). The resolution is 65 commodities by 65 sectors, given by the NACE*64 industry level.
- Extensions - Greenhouse gases (unit of tonne CO2 equivalent), from Eurostat table Air emissions accounts by industry and households (NACE Rev. 2) [env_ac_ainah_r2].
- Extensions – Employment (unit of 1000 persons), from Eurostat table Employment by sex, age and detailed economic activity (from 2008 onwards, NACE Rev. 2 two-digit level) [lfsa_egan22d].

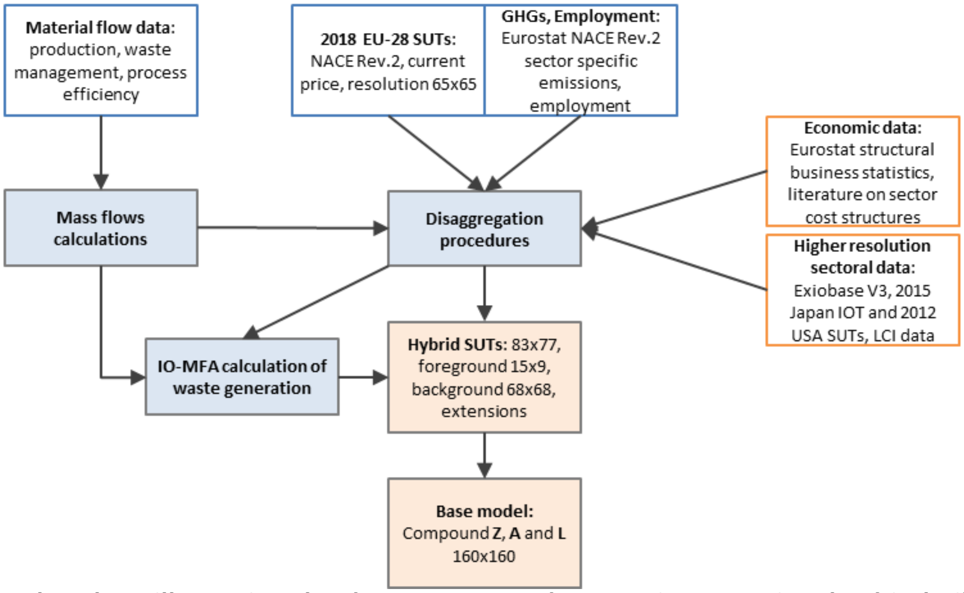

Figure S5: Flowchart illustrating the data sources and processing steps involved in building the base hybrid IO model. White boxed denote base data or tables (blue outline), data used in disaggregation procedures (orange outline), blue boxes denote calculation routines, and orange boxes denote output tables.

2.1 Overall approach

Starting from the base EU-28 tables, we built a single-region model, with specific representation of commodities and sectors pertaining to the lifecycle of plastic packaging. These additional commodities and sectors were essentially disaggregated from their parent base sectors. We denoted them as the foreground sections of the model tables, consisting of an upstream section containing production processes, and downstream section containing waste management activities. The foreground is fully integrated with the rest of the economy, also denoted as the background.

The base tables were extended by disaggregating sectors and commodities/services which represent the foreground, namely production and waste management of plastic packaging. In addition to foreground sections, it was necessary to disaggregate the energy sector and the chemicals production sector (within the background). This was done to accommodate the production of energy from plastics within waste management (WtE) and the absorption of recycled plastics within other production sectors than packaging production (e.g., conversion to products used in the construction sector). In total we added 15 commodities

and 9 sectors, bringing the final SUTs to the dimension of 83 by 77. The full tables are available in the excel file of SI B, and a graphical representation in illustrated in Figure S6.

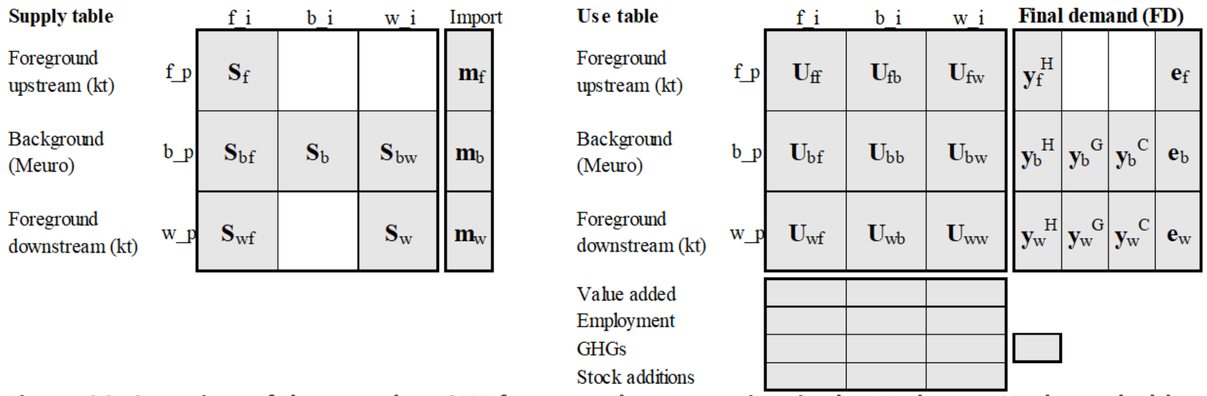

Figure S6: Overview of the complete SUT framework, superscripts in the FD denote H – households + non-profit organizations, G – government expenditure, and C -- gross fixed capital formation.

Table S9: Description of sets and tables used in the model

**Description of sets in the model framework**		
**Symbol**	**Description**	**Components**
f	Foreground upstream	Products/industries: Primary polymer, Secondary production (recycling), Conversion to packaging (3 commodities by 3 sectors)
b	Background (rest of economy)	Monetary products and sectors (68 commodities by 68 sectors)
w	Foreground downstream	Waste streams and waste management sectors (12 commodities by 6 sectors)
fd	Final demand	Components of final demand (households, non-profit organizations, government, gross fixed capital formation, and exports)
r	Value added, stocks and extensions	Taxes less subsidies, compensation to employees, mixed income (gross), employment, greenhouse gas emissions, stock additions

**Description of the tables in the model framework**	
**Name**	**Description**
**S** and **V**	Supply matrix and make matrix (transpose of **S)**
**U, U_{dom}, and U_{imp}**	Use matrices: total, domestic and import
**FD, FD_{dom},** and **FD_{imp}**	Final demand matrices: total, domestic and import
**B, D**	Denote use coefficient matrices for **U** and market share matrices for **S**
**Z_{su}, A_{su}**	Denote the square compound transaction matrix and its coefficient form (dimension of 160x160), **Z** and **A** represent the simple transaction matrix and its coefficient form
**R**	Matrix of extensions (value added, stocks)
**m**	Import vector
**y**, **e**	Final demand vector (representing components (with superscripts) or their sum) and export vector
**q**, **g**	Vector of total product output and total industry output (in relation to **S**)
**x**	Compound vector of total domestic output (**q** and **g** combined)
**p**	Packaging product intensity (kt per MEUR)

The disaggregation was performed at the level of SUTs. In the SUT framework, new sectors were built, representing disaggregated portions of the original sectors[35]. The constituent parts of new commodities and sectors were removed from the original sectors, to avoid double-counting[36].

The outputs and inputs of new sectors (columns vectors in the Supply and Use matrices) were allocated manually based on several sources of data, such as subsector turnover and added value shares from Eurostat, the structures of more detailed SUTs or IOTs (Exiobase V3, Japan and USA), the size of waste streams and sector operational costs (for waste sectors), and LCI data for certain sector inputs such as electricity. Allocation along production and the use of commodities in the economy (row vectors in the Supply and Use matrices) was specific for new commodities pertaining to the foreground (e.g., use of packaging). A default allocation was used only for energy commodities, essentially using the row commodity share of the original sector, adjusted by subtracting the manually allocated values, to preserve commodity output total[35].

Within the table framework, we maintained Use and Final demand that distinguish domestic products from imported products. Disaggregation was first performed at the level of total use of products. Second, total Use was split into domestic and imported shares following the ratios between the original aggregated commodities.

2.2 Disaggregation procedures

2.2.1 Disaggregation in the background

The base tables (65x65) were extended by disaggregation of the Energy production sector (D35) to three subsectors and the split of the Chemical and chemical products sector (C20) in two sectors, one covering production of plastics and the second all remaining chemical production. The result are tables with the dimension of 68 commodities by 68 sectors.

In the disaggregation procedure, data on subsector turnover shares within a main sector was used as indicator for the total output of a disaggregated sector in the Supply matrix. Similarly, subsector value added shares was used to disaggregate components of the Value Added table extension. Considering that $\mathbf{iS}=\mathbf{iU}+\mathbf{iVA}$ (where $\mathbf{VA}$ is the matrix of value added and $\mathbf{i}$ is a row summation vector of ones), the total of sector inputs in the Use matrix could be determined as the difference between sector supply total and value added total. The allocation of column and row entries in further described below for the sectors.

Data for a number of economic indicators for structural business statistics are available from Eurostat, which include for example sector turnover, value added, and employment. For production sectors (NACE Rev. 2, sections B-E), these indicators are available at a more sectoral detailed level, including several subsectors. These can be retrieved from the Eurostat database table: Industry by employment size class (NACE Rev. 2, B-E) [SBS_SC_IND_R2].

(1) Energy production

The Energy sector contains aggregated: electricity power supply, electricity distribution, steam and air conditioning supply, and gas production and distribution. The sector was disaggregated to three subsectors: (1) Electricity power supply, (2) Steam and air conditioning supply, and remaining (3) Electricity distribution, gas production and distribution.

The above procedure (in 2.2.1) was used to disaggregate directly Steam and air conditioning supply (D353). Column entries in the Supply and Use matrices for this new sector, were then allocated with the default procedure, i.e., using the distribution of column entries in the original aggregated sector.

Electricity production and distribution are aggregated under a subsector in Eurostat (D351), while the remaining sector represents Manufacture of gas and distribution of gaseous fuel (D352). To split a standalone electricity production sector, we used the sector structures of Exiobase v.3. Specifically, the multi-regional SUTs (MR-SUTs) of Exiobase v.3 (current price) for the year 2011 [37] were first aggregated for the combined EU-28 and RoW regions, and then commodities and sectors were further aggregated to the 68x68 dimension used in our tables. As Exiobase follows largely the CPA classification, the construction of a correspondence matrix is straight forward. Column entries in the Supply and Use matrices for the new Electricity power supply sector were calculated using the share of this sector in Exiobase. Row entries in the Supply matrix for the new commodity of electricity were also based on Exiobase, while in the Use matrix, entries followed the default distribution of the original aggregated commodity. This also applied to entries of energy products in the Final Demand matrix.

The values determined such for the new sectors constituted a first order result. To validate the use of this result, the sector output, specifically the value (price) of the electricity commodity was checked with data from Eurostat energy production statistics. Total volume of electricity production for 2018 is available from Production of electricity and derived heat by type of fuel [NRG_BAL_PEH], and electricity prices from Electricity price components [NRG_PC_205_C]. Taking the row sum value of the new electricity commodity in the Supply table and dividing this value by the total electricity production reported by Eurostat, yielded a price of 86 EUR/MWh (=281,816 MEUR/327,278 GWh). This price was deemed reasonable when compared to Electricity price components data and therefore the disaggregation result was taken forward in the model.

(1) The Chemical sector

Plastic packaging is produced almost entirely of thermoplastics, with the resins PE, PP and PET constituting around 90% of feedstock[3]. Therefore, the new Manufacture of plastics sector was focused on the production of thermoplastics.

As first step to separating a plastics production sector from C20, turnover and value added were used to disaggregate the broader sector C201 - Manufacture of basic chemicals, fertilizers and nitrogen compounds, plastics and synthetic rubber in primary forms. The new sector for Manufacture of plastics was then disaggregated from C201 taking the total value of main thermoplastics in Eurostat production statistics as indicator for sector output (Table S10). We assume that the new sector covers the entire production, i.e., of-diagonal row entries in the Supply of the new commodity are 0. The new sector value added was assumed to follow the parent sector. The Use sector inputs were computed using input coefficients for thermoplastics based on the IOT of Japan for 2015[a], which details 12 types of plastics. As it is expected that the use of thermoplastics in different sectors is substantially different that the use of the original aggregated commodity, we compared Use row entries for primary plastics in the 2011 version of Exiobase, the 2012 SUTs of the US[b], and the 2015 IOT of Japan. The last of the three was chosen for the elaboration of row entries, as it is most

[a] https://www.e-stat.go.jp/en/stat-search?page=1&toukei=00200603

[b] https://www.bea.gov/industry/input-output-accounts-data

detailed regarding specific plastic polymers. It was assumed that no primary plastics are consumed by Final Demand categories, except for amounts that were exported extra-EU.

Table S10: Primary plastics production statistics for 2018, used to dimension the disaggregated sector

Code	Label (Eurostat PRODCOM)	Value<br>MEUR	Mass<br>kt	Unit value<br>EUR/kg
20161035	Linear polyethylene having a specific gravity < 0,94, in primary forms	2358	2332	1.01
20161039	Polyethylene having a specific gravity < 0,94, in primary forms (excluding linear)	4579	4151	1.10
20161050	Polyethylene having a specific gravity of >= 0,94, in primary forms	4741	5711	0.83
20162035	Expansible polystyrene, in primary forms	2348	1504	1.56
20162039	Polystyrene, in primary forms (excluding expansible polystyrene)	1549	1786	0.87
20163010	Polyvinyl chloride, not mixed with any other substances, in primary forms	3988	5198	0.77
20163023	Non-plasticised polyvinyl chloride mixed with any other substance, in primary forms	479	364	1.32
20163025	Plasticised polyvinyl chloride mixed with any other substance, in primary forms	1051	872	1.21
20164062	Polyethylene terephthalate in primary forms having a viscosity number of >= 78 ml/g	3326	3049	1.09
20164064	Other polyethylene terephthalate in primary forms	493	331	1.49
20165130	Polypropylene, in primary forms	10418	10192	1.02
20165150	Polymers of propylene or of other olefins, in primary forms (excluding polypropylene)	3662	2578	1.42
20165390	Acrylic polymers, in primary forms (excluding polymethyl methacrylate)	5400	4200	1.29
		**44392**	**42268**	**1.050**

2.2.2 Disaggregation of foreground upstream

The foreground upstream included the production of primary plastics which will be used for production of packaging, the secondary production of recycled plastics, and the process of conversion of plastics to packaging.

(1) Primary plastics used for packaging production

The previously constructed sector of Manufacturing of plastics (in the background section), was split now again into two sectors, one covering the foreground Primary plastics for packaging and a remaining sector that provides raw material for plastic products other than packaging. After this split, the later sector replaced the previous sector in the background.

The new foreground sector was dimensioned using the known physical amount of domestic primary plastics used for packaging and the unit price determined previously (Table S10).

(2) Secondary production of recycled plastics

Coarse information on the size of the plastic recycling industry in Europe was reported by Plastics Recyclers Europe (2020). They indicate that the industry has a total turnover of around 3,000 MEUR. The share of this industry that is dedicated to plastics from packaging is likely more than 80%[8]. Recycling sector cost structure data (capital and operational costs) can be found in a number of sources, including studies on packaging Extended Producer Responsibility[39,40], and studies addressing plastic packaging in particular[21,41].

Considering the above, the sector output was dimensioned using the know physical amounts of secondary plastics and an average price for this material. The prices of secondary plastics are generally lower than for primary plastics and suffer significant fluctuations. The average price of 0.75 EUR/kg was used in this work.

In the EU-28 SUTs, plastic recycling is part of the overall sector E37-E39 Sewerage; waste collection, treatment and disposal activities; materials recovery; remediation activities and other waste management services. Eurostat subsector data is available for E383 Materials recovery. We used this data to define the Value added constituent part of sector inputs for the new sector. Major Use (intermediary) inputs to the sector were manually allocated, namely the foreground input (purchase) of sorted plastics as feedstock, the cost of treatment for recycling residues, and the input of electricity to the recycling process. The first two were calculated based on their physical mass and unit price (elaborated in following foreground downstream section), while the latter was based on LCI data for recycling processes (average value of around 500 kWh/t) and the previously determined price of electricity (86 EUR/MWh). The remaining intermediary inputs to the sector were dimensioned using the broader E383 Materials recovery sector. No of-diagonal entries were allocated for the new commodity, meaning that no other sectors recycle plastics in the model.

(3) Conversion of plastics to packaging
Sector C22 Manufacture of rubber and plastic products, consists of two subsectors, namely C221 Manufacture of rubber products and C222 Manufacture of plastic products. The overall size of the two subsectors can be estimated based on turnover and value added shares, as previously described. Further, a new sector for the Manufacture of packaging was disaggregated from C222 Manufacture of plastic products. The total sector output was determined based on the known physical amount of converted packaging and a determined unit price. The unit price of 2.52 EUR/t represents the weighted unit price of PRODCOM codes that can be identified as plastic packaging products.

Regarding sector inputs for the new Manufacture of packaging, it was assumed that value added components followed the parent sector C222. Intermediary inputs consisted of the manually allocated primary and secondary feedstocks, while remaining inputs were allocated based on the input coefficients of packaging production in the 2015 IOT of Japan. The remaining “residual” sector replaced the previous C22 sector in the background.

It was assumed that the new sector produced all the packaging product used in the economy, therefore no of-diagonal row entries in the Supply were included.

Row entries in the Use matrix, which represent the use of converted packaging by economic sectors, were determined with the following approach:

(Step 1) The use of plastic packaging across the economy in the US 2012 SUT was used as proxy to estimate the packaging use the EU. This was deemed a reasonable approach and was chosen over the Japanese IOT, considering the more similar size and diversity of the EU and US economies. First, the 2012 US SUT was converted to IOT (405 product by product dimension) using the industry technology construct. Product packaging intensity for the US (in MUSD/MUSD) was then calculated by dividing elementwise the row vector of packaging input to sectors by the vector of total product output ($\mathbf{q}^{US}$).

(Step 2) Next, the vector of US packaging intensity was aggregated from 405 to the 68 commodities in the background section of our model, and the currency was converted to MEUR. The resulting intensity vector was multiplied elementwise with the vector of

product output in the EU-28 ($\mathbf{q}^{EU}$), and the resulting vector was then rescaled to sum to the value in MEUR of total converted packaging used in the EU (this is 20551kt*2.52MEUR/kt). This vector represents the monetary use of packaging by sectors in absolute terms.

(Step 3) The vector of packaging intensity per product, denoted now as **p** with values in the unit kt/MEUR, was found by fist dividing the values of the vector calculated in the previous step by the price of converted packaging, which yields a vector of packaging use by sector in absolute physical terms (kt). Last, further dividing the resulting vector elementwise by the vector of product output ($\mathbf{q}^{EU}$) results in a vector of packaging intensity.

(Step 4) The row entries in the Use matrix, denoting consumption of packaging, were calculated by multiplying the packaging intensity per product, with the domestic product output and the market share matrix **D**, as given in the equation:

$$\mathbf{U}_{\mathrm{f}\cdot}^{P} = \mathbf{q}_{\mathrm{b}}^{\mathrm{T}}\,\mathrm{diag}(\mathbf{p})\mathbf{D}_{\mathrm{b}}{}^{\mathrm{T}} = \mathbf{q}_{\mathrm{b}}^{\mathrm{T}}\,\mathrm{diag}(\mathbf{p})\,(\mathbf{S}_{\mathrm{b}}{}^{\mathrm{T}}\mathrm{diag}(\mathbf{q}_{\mathrm{b}})^{-1})^{\mathrm{T}} \qquad \text{(S1)}$$

The superscript ^{T} denotes transposition, ^{p} denotes the row for the packaging product in **U**, and the notation "diag" before a vector refers to the diagonal matrix with the elements of the vector along the main diagonal and zeros elsewhere.

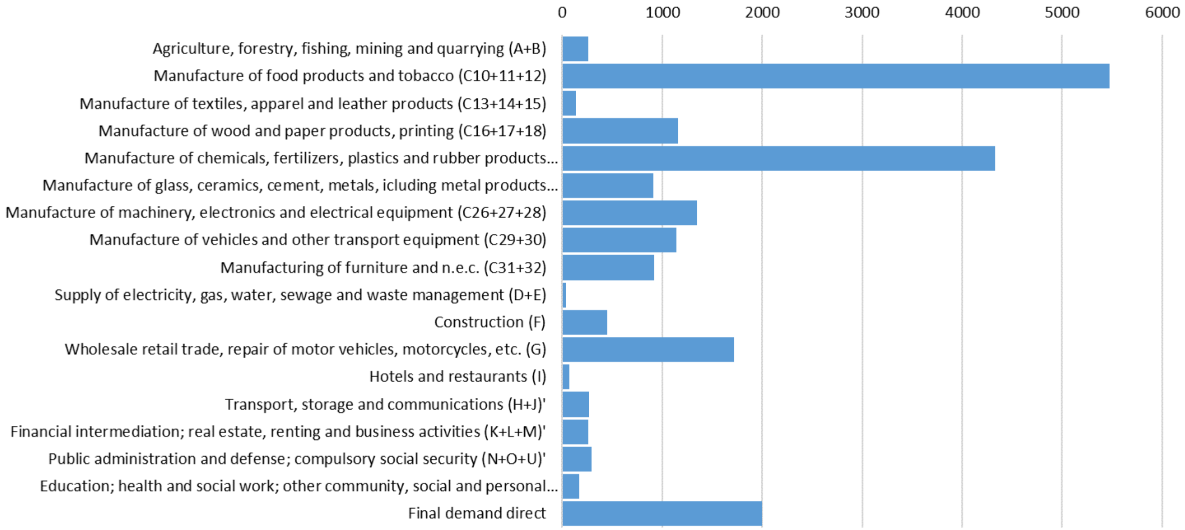

Figure S7: Plastic packaging use in the EU-28 2018 [kt] by 17 ISIC aggregated categories and direct use by final demand.

2.2.3 Disaggregation of foreground downstream

The foreground downstream included a total of 12 commodities (waste streams), processed by 6 sectors, including collection (separate and mixed), sorting, and final treatment by WtE and landfill (regular and inert landfill).

The structure of the foreground downstream in the model follows the principles of WIO[1] and more specifically WSUT[2]. Packaging waste generation is connected to sectors or final demand categories in the Use matrix and should be interpreted as demand for waste management service. The total waste management used/demanded in the economy is placed in the Supply matrix, as the output (product/service) of waste management sectors. In physical terms (kt) waste represents the quantity generated or treated, while in monetary terms waste is the equivalent value (price) of collection or treatment. Moreover, waste

management sectors generate so called secondary waste streams, and therefore themselves demand service from other treatment sectors. Certain waste sectors produce other commodities with a positive economic value besides the waste service, such as sorted plastics and energy, which can replace the equivalent commodity produced in background sectors. The upstream production and the downstream are connected, as plastics recovered downstream become input to production upstream (the circular flows), while waste generated upstream is handled by downstream sectors.

Before the downstream sectors could be dimensioned and disaggregated from background sectors, packaging waste generation needed to be simulated, including imports and exports with packaged goods. This was based in the IO-MFA approach and will be elaborated below.

Waste management sectors were then constructed, and their constituent parts were removed from original aggregated sector E37-39 Sewerage; waste collection, treatment, and disposal activities; materials recovery; remediation activities and other waste management services.

The modelling of waste generation
A general description of the waste streams covered by this study was given in Section 1.3. Here we describe the model equations used to calculate pre-consumer waste, additions to stocks, and post-consumer waste.

In the equations following, γ^{pack}, γ^{retail}, and $\gamma^{service}$ denote vectors of packing, retail, and service waste rates applied per product, with elements taking values [0:1]. $\boldsymbol{\varphi}$ denotes a scalar representing a net addition to stock rate applied to products. To calculate flows associated with imported products, $\mathbf{q}_b$ is replaced by $\mathbf{m}_b$. * expresses the Hadamard product

Pre-consumer waste: $\boldsymbol{\Gamma}_{w\cdot}^{pre} = (\mathbf{q}_b * (\boldsymbol{\gamma}_b^{pack} + \boldsymbol{\gamma}_b^{retail} + \boldsymbol{\gamma}_b^{service}))^T \ (\mathrm{diag}(\mathbf{p})\mathbf{D}_b{}^T)$ (S2)

Addition to stock: $\boldsymbol{\sigma} = (\mathbf{q}_b * \ (1 - \boldsymbol{\gamma}_b^{pack} - \boldsymbol{\gamma}_b^{retail} - \boldsymbol{\gamma}_b^{service}))^T \ \boldsymbol{\varphi} \ (\mathrm{diag}(\mathbf{p})\mathbf{D}_b{}^T)$ (S3)

Post-consumer waste generated by industries and final demand:

$$\boldsymbol{\Gamma}^{post.i} = (\mathbf{q}_b * (1 - \boldsymbol{\gamma}_b^{pack} - \boldsymbol{\gamma}_b^{retail} - \boldsymbol{\gamma}_b^{service})(1 - \boldsymbol{\varphi}))^T \ (\mathrm{diag}(\mathbf{p})\mathbf{B}_{b\cdot}) \quad \text{(S4)}$$

$$\boldsymbol{\Gamma}^{post.fd} = (\mathbf{p} * (1 - \boldsymbol{\gamma}_b^{pack} - \boldsymbol{\gamma}_b^{retail} - \boldsymbol{\gamma}_b^{service})(1 - \boldsymbol{\varphi}))^T \ \mathbf{FD} \quad \text{(S5)}$$

The addition to stock was not recorded in the Use matrix, but as an extension as shown in Figure S6. The Use matrix rows pertaining to the generated waste were not populated directly with the $\boldsymbol{\Gamma}$ vectors calculated with equations S2, S4, and S5. The pre-consumer and post-consumer streams were first split between shares that belong to separate collection and to mixed collection. Essentially, this endogenizes the treatment allocation which in WIO is done exogenously though an allocation matrix. As sector specific or final demand category specific shares of separate collection are not known, we implemented a fixed share for both waste streams and all waste generators. This was calculated as 0.38:0.62 in 2018 and reflects the ratio of waste collected separately to mixed waste collected fore treatment. Specifically, the model estimated the total packaging waste generated in the EU-28 to be 19600 kt, and we know that total packaging waste collected for recycling was 7500 kt (section 1.3 Table S6). The model can accommodate sector specific efficiencies for separate collection when these become available.

Disaggregation of waste management activities
The size of the waste sectors was determined by combining physical flows and connected economic data. The latter was based on sector cost structures, for which data was collected

from different sources, such as capital and operational costs, gate fees and prices for different outputs. Sources include studies on the economics of packaging Extended Producer Responsibility[39,40], sorting facilities[33], and studies addressing plastic packaging[21,41]. Intermediary sector inputs and value added were allocated to each sector as further elaborated in sections below. An important, starting, source of information was Eurostat subsector data available for sector E37-39, which is comprises of E37 Sewerage, E381 Waste collection, E382 Waste treatment and disposal activities, and E383 Materials recovery.

It is important to state here that the demand for waste service, and therefore connected costs, were allocated always to the waste generators, i.e., sectors or final demand. This includes the costs of collection, sorting, and treatment by WtE or Landfill. This approach is correct for mixed waste collection and its treatment but could be modelled differently for separate collection and sorting. We refer here to the explicit modelling of extended producer responsibility (EPR) as done by Rodrigues et al. (2016)[42]. Explicit modelling of EPR could reflect that specific fees are collected from producers, importers, and distributors/retailers of packaged goods, with the intent to cover certain portions of the management chain for packaging waste. However, how EPR is implemented in EU-28 differs widely between member states, in terms of the value of EPR fees and actual coverage[40]. Denmark and Hungary, as exceptions, do not have an EPR system for packaging waste. Therefore, capturing the complexity of EPR systems was not in the scope of this work. Moreover, the EPR fees incurred by producers, importers, and distributors/retailers of packaged goods, are ultimately passed on to the consumers of these goods through their prices. As such, our approach is likely valid.

The final sector economic balances are resumed in tables S11. The gate fees in table S11 can also be understood as unit prices for the different types of treatment.

Table S11: Economic balance for the waste management sectors, all units are EUR per ton process input

Sector	Collection separate	Collection mixed	Sorting	Waste-to-Energy	Landfill	Recycling
**Sector output**	**186**	**50**	**195**	**263**	**73**	**607**
Gate fee (service)	186	50	70	50	73	-
Secondary plastic	-	-	-	-	-	607
Sorted plastics	-	-	125	-	-	-
Electricity	-	-	-	83	-	-
Heat	-	-	-	130	-	-
**Sector input**	**186**	**50**	**195**	**263**	**73**	**607**
Sorted plastics	-	-	-	-	-	160
Waste fees*	-	-	56	28	-	16
All other intermediary	93	25	89	81	38	276
Value added**	93	25	50	154	35	155

* shown only for secondary waste (processing waste)

** includes here taxes less subsidies on products, compensation of employees, gross mixed income (including operating surplus)

(1) Waste collection

The waste collection sector output was defined by the physical amount collected and specific waste collection fees. The monetary value was the multiplication product of the two. Sector inputs and commodity use (row entries) were disaggregated by using the monetary value of collection service. Sector inputs, column entries in the Use matrix, were allocated using the coefficients for H50 Land transport, and subtracted from the aggregated waste management

sector in the background “E37-E39”. Row entries in the Use matrix and the Final Demand are the physical amounts of generated waste collected by the two sectors. These entries represent generated waste but also the demand for collection service by sectors and final demand categories. Their values are subtracted from the aggregated waste service commodity in the background “CPA_E37-E39”.

(2) Sorting of plastic packaging waste

The sorting sector output is composed of a sorting gate fee charged to waste generators and revenues from the sale of sorted (bales) plastics, which are purchased by the recycling sector or exported. Sector inputs, column entries in the Use matrix, included the waste treatment fee that the sector incurs for the sorting residuals, specific electricity costs (LCI based), and remaining entries were allocated using coefficients calculated for E383 Materials recovery. Row entries in the Use matrix and the Final Demand, constitute the sum of pre- and post-consumer waste which is collected separately for recycling. This applies for physical units, while to convert to monetary values, the sorting gate fee is multiplied with the physical values.

(3) WtE of packaging waste and secondary waste

WtE for plastic waste presents some particular challenges. The plastic waste fraction may contribute around 25% of the energy content on mixed MSW when incinerated, while it represents only around 10% of mass. This means that plastics contribute significantly to sector revenues from sale of energy and removal of plastic fractions would significantly affect the cost balance of MSW incineration[43]. We attempted to reflect this in the cost structure applied in the disaggregation of this sector.

Hestin et al. (2015) identified the average gate fee for incineration in Europe around 88 EUR/t. This represents net costs after sale of energy. In this work we estimated an average price of electricity of 86 EUR/MWh and for heat of 13.5 EUR/GJ in 2018. Considering average net efficiencies of 12% electricity and 33% heat, based on a lower heating value of average mixed MSW in Europe of 10 MJ/kg, the revenues from energy sales can be approximated to 29 EUR/t for electricity and 45 EUR/t for heat. Summing the gate fee and energy sales, should equal an estimate of total revenue which also equal the total gross costs of incinerating mixed MSW, namely 161 EUR/t.

We then assumed that this gross cost is relatively similar when spread over the different waste fractions in MSW including plastics. The revenues from energy sales by incinerating one ton of plastics (with the same average incineration plant efficiencies) can be approximated to 83 EUR/t for electricity and 130 EUR/t for heat. These were already higher than the total costs for incinerating MSW. Last, we also assumed that the gate fee charged for plastics is 50 EUR/t to consider that plastic rich waste streams from industry might be charged by the incineration sector less than general mixed waste. Summing energy sales and the gate fee, brings total revenues at 263 EUR/t for plastics. The difference between costs and total revenues 263-161=102 EUR/t for plastics can be considered part of the operating surplus, pertaining to plastics. In this way we reflect the plastics contribute more to sector value added, compared to mixed waste fractions with less energy content.

Sector inputs, column entries in the Use matrix, included the waste treatment fee that the sector incurs for the incineration residuals (e.g., bottom ash), and remaining entries were allocated using coefficients calculated for E382 Waste treatment and disposal activities. Row entries in the Use matrix and the Final Demand, constitute the sum of pre- and post-consumer waste, which is collected mixed for treatment, which was split between the two

main treatments of WtE and Landfill. An overall ratio between these forms of treatment was applied homogeneously to all entries, namely 60:40[3] in 2018.

(4) Landfilling of packaging waste and secondary waste

The Landfilling sector output was defined by the physical amount of waste undergoing this treatment and specific gate fees. Sector inputs, column entries in the Use matrix, were allocated using coefficients calculated for E382 Waste treatment and disposal activities. Row entries in the Use matrix and the Final Demand, were determined as described in the previous section.

2.2.4 Disaggregation of extensions

The two extensions included in this work constituted emissions of Greenhouse gases (GHGs - unit of tonne CO2 equivalent) and Employment (unit of 1000 persons). The extensions represent direct contributions per sector. The GHGs inventoried in the Eurostat table "Air emissions accounts by industry and households (NACE Rev. 2)", comprise carbon dioxide (CO_2), nitrous oxide (N_2O), methane (CH_4) and fluorinated gases (hydrofluorocarbons (HFC), perfluorocarbons (PFC), sulphur hexafluoride (SF_6) and natrium trifluoride (NF_3)). The emissions are expressed as Global Warming Potential over a 100-year time horizon (GWP100; IPCC, 2013).

In the case of Employment, sector specific intensities, could be determined from subsector information in Eurostat (Industry by employment size class (NACE Rev. 2, B-E) [SBS_SC_IND_R2]). This was combined with waste sector information from data sources such as Hestin et al. (2015). Sector employment in absolute figures was then subtracted from the parent sectors, to preserve overall employment in the EU economy.

The disaggregation of GHG emission was specific for the new sectors. For the upstream sector of Conversion to packaging, and the downstream sectors for Collection, Sorting and Landfilling, the allocation of emission was more generic, using sector turnover to split a share of emission from the parent sector. Emission associated with the WtE sector were calculated using an average intensity for the incineration of plastics, that is 2.5 kg CO_2eq. per kg. Individual polymer intensities span between 1.6 and 3.15 kg CO_2eq per kg plastic[44].

The determination of direct emissions for Primary and secondary plastics production was more complex. The production of primary plastics generates emissions in upstream production of feedstocks (naphtha, ethane, and LPGs), in the production of monomers via stream cracking and aromatic synthesis, and subsequently in polymerization processes to produce end polymers. The production of primary plastics occurs in large and complex petrochemical clusters which make the attribution of carbon emissions to plastics very difficult [45]. Sectoral emissions collected by Eurostat, do not distinguish plastics as a separate sector. Available LCI data is generally outdated, such as the Plastics Europe Eco-profiles which are now 20 years old, but still form the base for polymer production processes in the Ecoinvent database. An additional challenge is that most available LCI data is aggregated cradle-to-gate, therefore it is not possible to distinguish direct and indirect emissions.

Looking at the C20 Chemicals and chemical products sector, emissions intensities (absolute emissions normalized by sector output) have been continuously decreasing over time, with approx. 20% total decrease between 2008 and 2018. Over the past decades significant improvements have been made in energy efficiency, i.e., decrease in fuel and power consumption. According to the International Energy Agency[46], conversion losses in the major chemical production processes are now close to their theoretical minimums. It is very

likely that emissions per unit production have changed significantly also for plastics. A clear example of this is given by the study on PET by Franklin Associates (2020), which found that the footprint of PET decreased by 20% in the US between 2011 and 2018, from 2.73 to 2.23 kg CO_2eq/kg.

The allocation of emission to Primary and Secondary plastics production followed a two-step approach: (1) an average total (direct + indirect) emission intensity was chosen considering literature information and potential reduction over time, and (2) the direct emission intensity that defined the total direct emissions, was determined by running the model sequentially and decreasing the intensity until the emission multipliers ($\mathbf{mb} = \mathbf{b}\,(\mathbf{I}-\mathbf{A})^{-1}$, where **b** denotes the vector of emission intensities and **mb** denotes the vector of emission multipliers) reach the level selected in step (1).

(1) Taking existing data, thermoplastics production has a footprint (cradle to gate) of between 1.7 and 3.5 kg CO_2eq/kg[44,48]. With the specific combination of plastics used in packaging, the average footprint falls around 2.3 kg CO_2eq/kg. We then made the assumption that this footprint is overestimated due to efficiency improvements in the last 10-15 years in Europe, and therefore decrease it by around 20% arriving at 1.9 kg CO_2eq/kg. In the case of recycling, production of secondary plastics, recent studies arrive at figures between 0.5 and 1 kg CO_2eq/kg[44,49]. In this work, we used the figure of 0.65 kg CO_2eq/kg, which accounts for the relative share of plastic types recycled in 2018. This figure does not account for the treatment of recycling residues.

(2) The direct emission intensity determined for primary plastics amounted to 1.4 kg CO_2eq/kg, and for recycled plastics to 0.3 kg CO_2eq/kg. The final emission multiplier for recycling was just under 1 kg CO_2eq/kg, as this accounts for treatment of recycling residues, and also all upstream impacts such as production of sorted plastics, which are the main input to recycling.

2.3 Base model

The disaggregation procedure yields a set of SUTs in mixed units. All physical entries can be converted to monetary values by virtue of the defined unit prices (or waste fees). The total size of the economy is the same as in the original Eurostat tables.

The basic Leontief demand-pull model, $\mathbf{x} = (\mathbf{I} - \mathbf{A_{su}})^{-1}\,\mathbf{y} = \mathbf{L_{su}}\,\mathbf{y}$ (where **y** is an arbitrary unit of final demand, **I** is an identity matrix of size $\mathbf{A_{su}}$, and $\mathbf{L_{su}}$ is the Leontief inverse) can be constructed both with tables in mixed and in monetary units. To maintain visible all waste flows and waste industries, we employed the Supply-Use formulation whereby square coefficient matrices can be determined with the industry technology assumption, as described by Lenzen and Rueda-Cantuche[50].

The SUT formulation satisfies the accounting balance as shown in the equation:

$$\begin{bmatrix} 0 & \mathbf{U} \\ \mathbf{V} & 0 \end{bmatrix} \begin{bmatrix} \mathbf{i}_p \\ \mathbf{i}_i \end{bmatrix} + \begin{bmatrix} \mathbf{y} \\ 0 \end{bmatrix} = \begin{bmatrix} \mathbf{q} \\ \mathbf{g} \end{bmatrix} \quad \text{(S6)}$$

where **i** are column summation vectors corresponding to products ^{p} and industries ^{i}.

The Leontief demand-pull model is given by the equation:

$$\begin{bmatrix} \mathbf{q} \\ \mathbf{g} \end{bmatrix} = \left\{ \mathbf{I} - \begin{bmatrix} 0 & \mathbf{B} \\ \mathbf{D} & 0 \end{bmatrix} \right\}^{-1} \begin{bmatrix} \mathbf{y} \\ 0 \end{bmatrix} \quad \text{(S7)}$$

Where **I** is an identity matrix, **B**=**U** diag(**g**)$^{-1}$ is the use coefficients matrix and **D**=**V** diag(**q**)$^{-1}$ is the market share matrix.

Before the model can be run in mixed units, monetary outputs which occur in the Supply matrix within the foreground sectors, have to be transferred to the Use matrix as negative entries. Without this operation it is not possible to calculate the industry output **g**. For example, the incineration sector has outputs/sales of energy (monetary) and waste treatment service (physical) which cannot be summed.

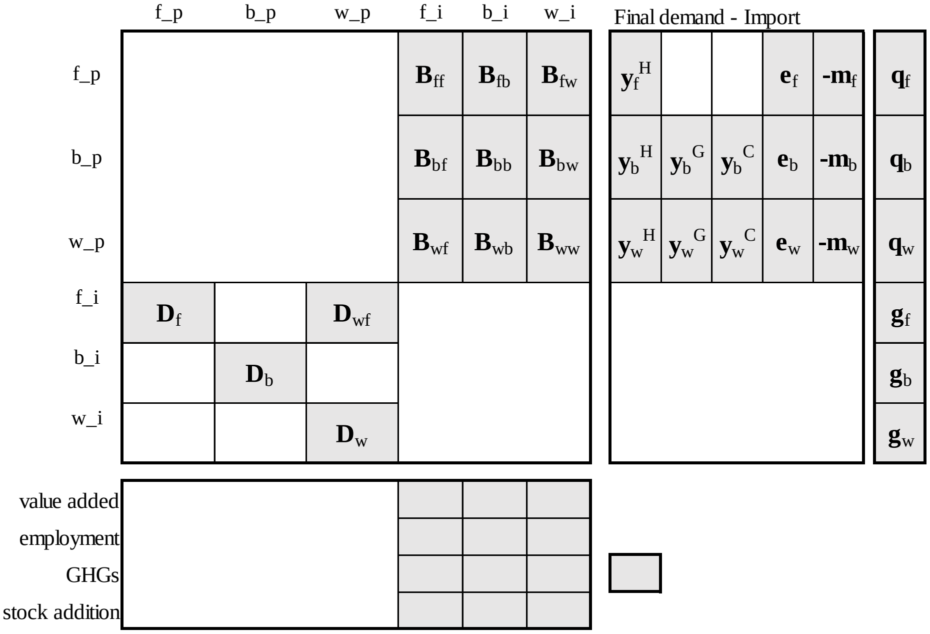

Figure S8: Graphical representation of the compound square coefficient matrix (A_{su}), final demand (FD) and extensions matrices (R). Superscripts in the FD denote H – households + non-profit organizations, G – government expenditure, and C - gross fixed capital formation.

In the application of the model, the Use of domestic and imported products is summed, thus we use a regional model with *indirect allocation of imports*. In the indirect allocation of imports, balance is maintained between domestic supply and use by netting total imports from total exports, such that final demand contains all final uses of domestic and imported products, minus the total of all imported products.

3 Projecting the model to 2030 and scenarios implementation

The objectives of the scenario analysis in this work are to: (1) estimate plastic packaging flows in EU to 2030, (2) map how flows could change with the implementation of CE interventions, including current policy targets, and (3) assess the potential environmental and socio-economic benefits and drawbacks from implementing CE interventions. Thus, we implement the analysis using a temporal dimension, with the objective to capture the effects of (short-term) economic development, as well as changing “background” conditions, i.e., decarbonization of the economy. We assess the impacts of these scenarios on three metrics, GHG emissions, employment and value added.

The temporal projection of IO tables can be accomplished by implementation of exogenous changes in: (1) the structure and size of Final demand components, (2) changes in the matrix of technical coefficients (**A**), including the input structure of sectors to reflect efficiency changes over time, or substitution between products, and (3) changes to environmental and social extensions.

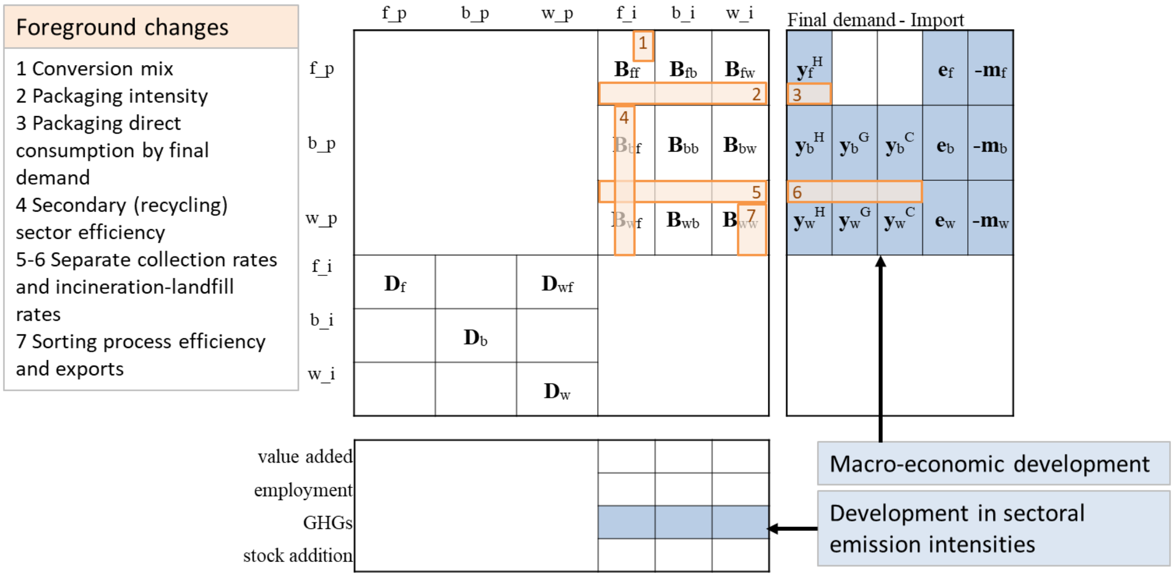

Figure S9: Indicative location of changes in the SUT system for implementation of scenarios. Blue colored locations denote background changes, vs. orange colored location which denote foreground changes.

In the description below we distinguish between the overall scenario describing the evolution of the EU economy, as the “background” scenario, and different “foreground” scenarios which consist of the actual circularity interventions within and related to plastic packaging. The background scenario encompasses the economic frame conditions that are common to all modelled scenarios.

3.1 Projection of the background frame

The projection of the system to reflect changes in the background EU economy to 2030, was done by implementation of exogenous changes to Final demand components and sectoral GHGs emission intensities. Considering the relatively short temporal scope, we refrained from making changes to technical coefficients in background sectors. Moreover,

as background sectors are more aggregated compared to for example the Exiobase classification, it was not possible to implement technology substitution directly, such as changing the electricity production mix over time[51].

The background scenario is driven by macroeconomic data projections, including the latest statistical data regarding GDP and demographics. This included population projections for EU-27 in Eurostat [table proj_19np] combined with UK population projections from the UN World Population Prospects 2019[c]. EU long-term projections of population and GDP were recently added with the 2021 Ageing Report[d]. For EU-28 GDP development between 2019 and 2023, we used the 2021 short-term economic forecasts by the European Commission, which includes the effects of the Covid-19 pandemic[52,53]. World GDP projection up to 2025 are available from the IMF World Economic Outlook Database[e]. The overall development in the background scenario up to 2030, following the shock of the Covid-19 pandemic, is still largely in line SSP2 - middle of the road[54].

The European Commission set out a vision for a climate-neutral EU in 2018. The vision was then endorsed by The European Parliament in its resolution on climate change in March 2019 and the resolution on the European Green Deal in January 2020[f]. As part of the European Green Deal, the Commission proposed on 4 March 2020 the first European Climate Law[g] to enshrine the 2050 climate-neutrality target into law. The evolution of the EU energy system, transport system and GHG emissions, has been comprehensively assessed with a suite of computational models for energy and GHG system analysis to formulate the EU Reference Scenario, a first version of which was published in 2016 followed by a 2020 update. The Reference Scenario presents a projection built on EU and Member States policies.

In the present work, we aimed to reflect EU decarbonization efforts, and this was captured in a simple manner by reducing GHG emission intensities in economic sectors. Specifically, for most sectors we extended the downward trend observed for the period 2008-2018, while for the Electricity production sector, we implemented a steeper intensity decline, consistent with intensity levels indicated by the European Environment Agency[h], that would allow the EU to achieve a net 55% reduction in greenhouse gases by 2030, compared with 1990. With this approach, the model showed a total for GHGs pertaining to economic activity (without direct emissions from households) above the most recent EU Reference Scenario 2020, but roughly in line with EU Reference Scenario 2016[55]. The resulting evolution of total industry emissions in EU-28, with and without the implementation of sectoral intensity reduction, is presented in Figure S10.

---

[c] https://population.un.org/wpp/Download/Standard/Population/

[d] https://ec.europa.eu/info/publications/2021-ageing-report-economic-and-budgetary-projections-eu-member-states-2019-2070_en

[e] https://www.imf.org/en/Publications/WEO/weo-database/2020/October/select-aggr-data

[f] https://www.europarl.europa.eu/doceo/document/TA-9-2020-0005_EN.html

[g] https://ec.europa.eu/clima/policies/eu-climate-action/law_en

[h] https://www.eea.europa.eu/data-and-maps/daviz/co2-emission-intensity-8#tab-googlechartid_googlechartid_googlechartid_googlechartid_chart_11111

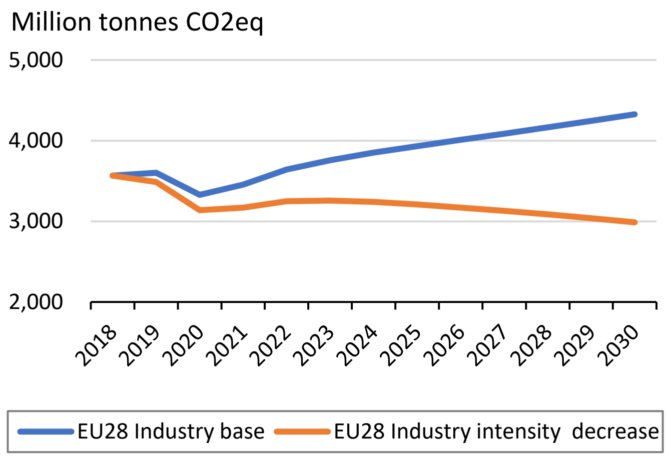

Figure S10: Development of total industry emissions with baseline and decreased intensities

EU-28 Economy projection

For the period 2019-2023, the different components of final demand were defined directly with data from the 2021 short-term economic forecasts by the European Commission which includes the effects of the Covid-19 pandemic. For the subsequent period up to 2030, we used the approach of Scott et al.[56], i.e., we applied econometric trends in the projection of the different components of Final Demand.

Trend equations were determined by linear regression, taking time series between 2005 and 2019. Time series for macroeconomic components were extracted from Eurostat in current year values as well as previous year values, which were used to deflate the series to the reference year 2018 prices.

Table S12: Predictors used for the different Final Demand components.

Final Demand component	Predictor
Household and NPISH final consumption expenditure	GDP per capita (EU GDP series normalized to the population series)
Final consumption expenditure of general government	EU-28 population development
Gross capital formation	Linear trend for the time series
Extra-EU exports	World GDP development

As can be observed in Figure S11 below, the chosen predictors explain relatively well the evolution of Final demand components. The figure also shows that the EU plastic converter demand for production of packaging is strongly linked to GDP development.

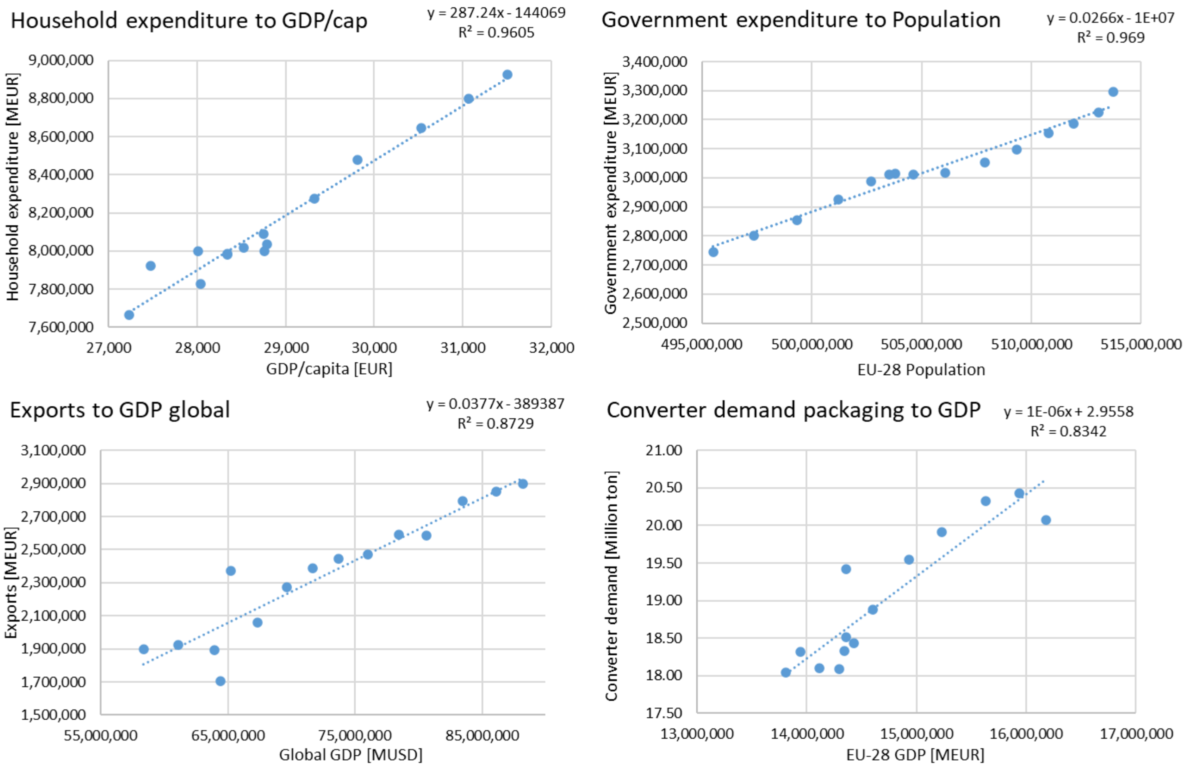

Figure S11: Regression results

The series for extra-EU Imports was calculated as the difference between total aggregated Final Demand and GDP in a year. This is based on the expenditure approach to calculating GDP, whereby GDP is the sum of consumer expenditure, government expenditure, investments, and net trade (value of exports minus imports). The projected time series for the Final Demand components were then converted to series of change factors. These represent the percentage change (increase/decrease) in a given year from the reference year 2018. The change factors are multiplied to the reference Final Demand components in the process of running the scenarios. Figure S12 displays the historical development and the projected time series. The time series are also given in the SI B excel files.

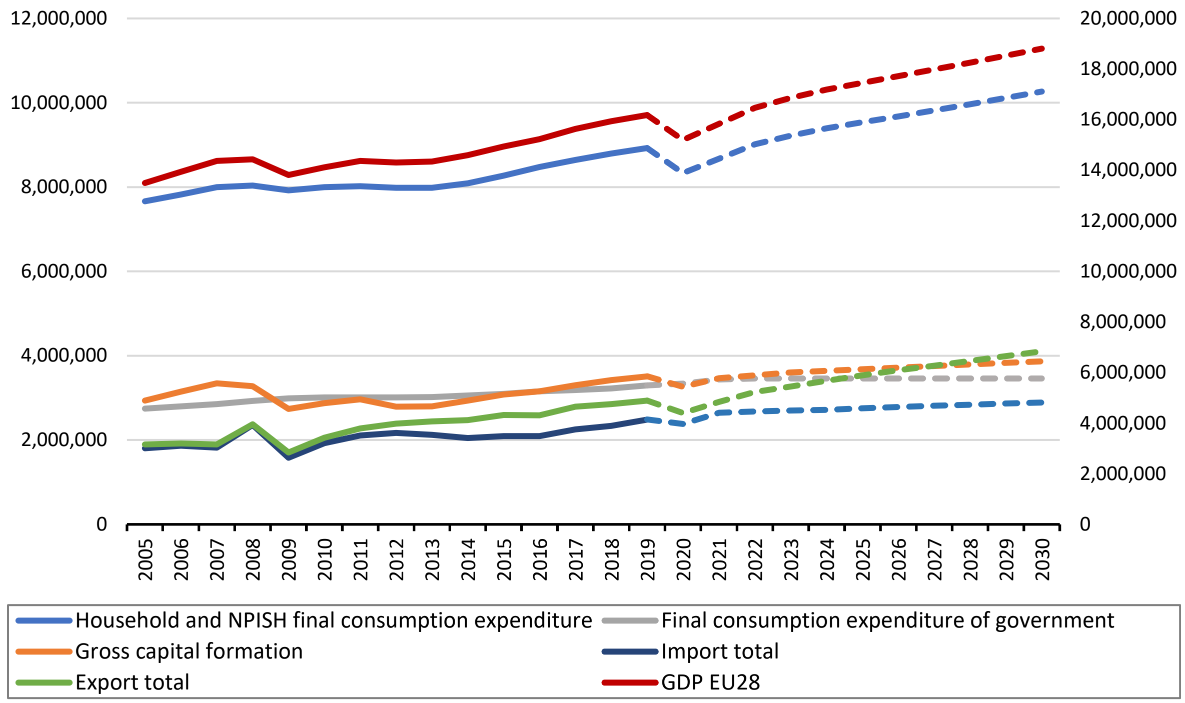

Figure S12: Time series for Final demand components, imports, and GDP (right axis), values in MEUR2018, historic series based on Eurostat table [naio_10_cp]

Sectoral emission intensities

The evolution of sectoral GHGs emission intensities was assumed to follow historical development. To calculate series of change factors that were applied in the model, emission intensities were first projected to 2030 with the trend given by linear ordinary least-squares regression[57].

The historical time series for sectoral intensities between 2008 and 2018, was calculated by normalizing total sectoral emissions (Eurostat table Air emissions accounts by industry and households) by sectoral output in a year (Eurostat table Supply, use and input-output tables - ESA 2010 - current prices). Both series are in the NACE Rev. 2 classification. The sectoral output series had to be first deflated, however a series in constant prices or in previous years prices was not available. Therefore, deflators were calculated using sectoral value added (as proxy) for which complete series were available in Eurostat.

The result of this first exercise is illustrated in Figure S13. The intensities in a few sectors appeared to increase, e.g., water and air transport, and were corrected in the projection to achieve a 20% decrease over the period 2019-2030. This is to reflect efficiency gains as projected in the EU Reference Scenario[58]. In addition, the intensities of a number of sectors, mainly service sectors with low emission intensity, were maintained constant. Lastly, the intensity decrease for the electricity production sector was accelerated to reflect a 60% decrease over the period, or a 2030 emission intensity around 100 g CO2eq./kWh.

Emission intensities, and change factors applied in the model are given in the related SI B excel files. The change factors applied to emission intensities in disaggregated foreground sectors followed the change in the original NACE Rev. 2 sectors.

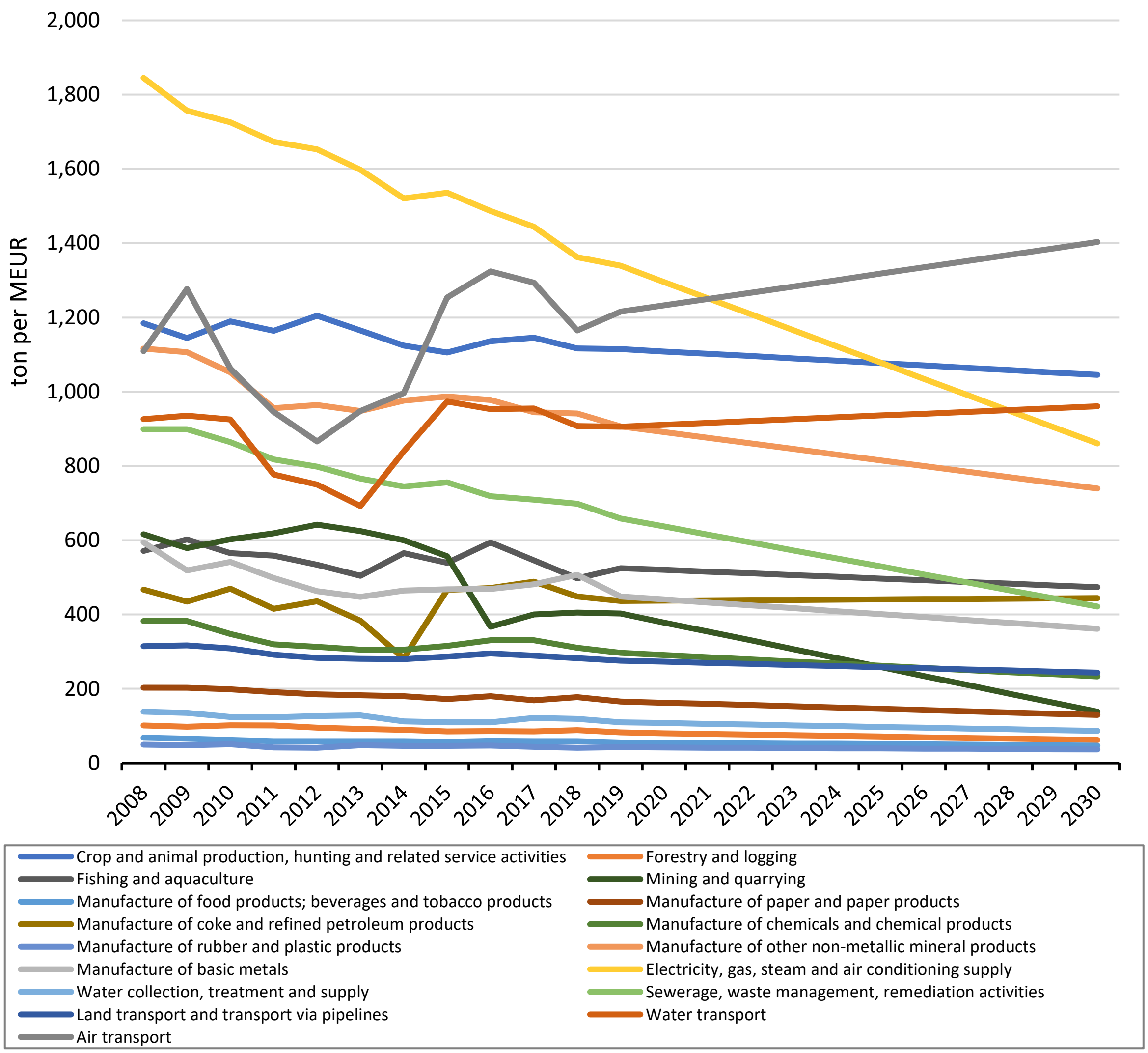

Figure S13: Historical (up to 2018) and projected emission intensities, for prominent sectors

3.2 Foreground scenario narratives and circularity interventions

European policy on Plastics

Plastics feature prominently in recent European policy, including the first and second Circular Economy Action Plans. Concrete targets and actions are put into legislation or committed as industry pledges. The main quantitative targets that were used to define foreground scenarios in this work are listed in Table S13.

Table S13: Main quantitative targets placed on plastics in EU legislation

Quantitative targets	2019	2020	2025	2029	2030	Source
All plastic waste recycled [%]					**50**	Strategy for Plastics 1/2018
Plastic packaging recycled [%]			**50**		**55**	Packaging and Packaging Waste Directive (EU) 2018/852
Beverage bottles separate collection [%]			**77**	**90**		Single-Use Plastics Directive (EU) 2019/904
Beverage bottles (PET) recycled content [%]			**25**		**30**	Single-Use Plastics Directive (EU) 2019/904
Consumption of lightweight plastic carrier bags reduced [units/person]	**90**		**40**			Directive regarding regulation and consumption of lightweight plastic carrier bags (EU) 2015/720 under 94/62/EC
Use of recycled plastic in new products, voluntary pledges [million tons]			**10**			Strategy for Plastics 1/2018
Recycled content in plastic packaging, voluntary pledge [%]					**30**	European plastics producers 2021

Overview of scenario narratives and circularity interventions

We modelled two Reference or business as usual (BAU) scenarios and defined three scenario narratives for the development of packaging use and packaging waste management up to 2030.

The two reference scenarios differ in one crucial aspect, that is product specific packaging intensity. In a first Reference, packaging intensity is maintained constant over the 12-year period modeled. In the second reference, packaging intensity was decreased by 1% every year. This second Reference is mean to reflect both dematerialization in the sector, as well as to partly reflect a move away from plastics for certain packaging, due to societal pressure. In the second reference scenario, sectoral monetary saving due to use of less packaging are internalized as consumption of research and innovation services. Economic sectors also experience monetary savings with waste management fees due to decreasing packaging waste generation, which has in turn been added to their value added.

The three scenario narratives combine four main interventions in the lifecycle of packaging plastics:

- Increase in recycling (as a product of collection*sorting*reprocessing efficiencies)
- Reduction of SUP consumption (replace by paper products)
- Reduction of exports of recovered plastics
- Increased closed-loop recycling
- Reduction of landfilling and increase in energy recovery from mixed waste

Increasing recycling constitutes the core of the three scenario narratives, while the remaining interventions were applied as sub-scenarios in all three narratives. The four interventions are implemented in the model through parameters, which are used as change factors to adjust either Final demand or elements of the technical coefficient matrix.

The recycling rate (*RR*) in a year, expresses the percentage of plastic waste supplied to the domestic market as secondary plastics, thus after reprocessing or secondary plastic production. Plastic waste here accounts for pre- and post-consumer packaging waste, but not production waste. I the absence of exports of sorted plastics, the recycling rate is also the product of three parameters, namely separate collection efficiency, sorting process efficiency, and reprocessing efficiency.

Main overall scenarios towards 2030 are:

(0) Reference or business as usual (BAU) – single scenario implementing the background economic projection while the foreground is maintained unchanged.

(1) Baseline development – this scenario narrative projects recycling over the period based on historical evolution of collection efficiency, and small improvements in sorting and reprocessing efficiencies. The time series for the packaging waste recycling rates from Eurostat was used as indicator for collection efficiency change. Consumption reduction measures are in line with adopted legislation on SUP and plastic bags. Exports follows a 10% reduction per year, in line with developments between 2018 and 2020. The ratio of secondary plastics returned to the packaging sector remains the same. The landfilling ratio of plastic waste not recovered for recycling decreases from 40% to 20%, in line with historical development (Eurostat packaging waste management [env_waspac]).

(2) EU targets – this scenario narrative implements the current targets for 50% recycling in 2025 and 55% in 2030. To achieve the targets, suitable collection, sorting and reprocessing efficiencies are taken. Consumption reduction measures are in line with adopted legislation. Exports follows a 10% reduction per year, in line with developments between 2018 and 2020. The ratio of secondary plastics returned to the packaging sector increases in order to satisfy the mandated recycled content rate of 30%. The landfilling ratio of plastic waste not recovered for recycling decreases from 40% to 10%.

(3) Max potential – this scenario narrative implements a recycling rate of 70% in 2030, deemed the maximum possible. To achieve the recycling rate, all three parameters were set at their maximum technical potential. Consumption reduction measures are in line with adopted legislation. Exports follows a linear reduction per year to achieve complete elimination in 2030. The ratio of secondary plastics returned to the packaging sector increases to 90%. The landfilling ratio of plastic waste not recovered for recycling decreases from 40% to 10%.

Individual interventions were added sequentially in the scenarios, in order to distinguish their effects on plastics flows and the selected scenario metrics. As such, scenarios (1) to (3) are implemented as groups of 5 sub scenarios, as below:

a) The first sub scenario includes only changes in recycling,
b) The third includes changes in recycling and SUP consumption reduction,
c) The fourth includes changes in recycling and reduction of exports,
d) The fifth includes changes in recycling and the increase in closed-loop recycling,
e) The last combines all interventions a) to d).

The evolution of recycling and disposal

To establish the evolution of the overall recycling rate in the Baseline development scenario narrative, we extrapolated the rate in the Reference 2018 system based on the past trend for recycling rates reported to Eurostat [env_waspac]. Following this, the rate was broken down to its components, knowing that overall recycling rate is the product of collection*sorting*reprocessing efficiencies. I this scenario narrative we allowed only small efficiency increases for sorting and reprocessing and changed primarily collection efficiency.

The packaging waste statistics in Eurostat, and here we refer to past reported recycling rates, reflect different calculation approaches in the member states. The rates were estimated to fall somewhere between a rate that would represent collection for recycling and a rate representing output after sorting processes[15]. European Commission has been clarifying and tightening reporting rules, the latest taking effect in 2020[59]. However, the effect of this will only be seen in the future.

The overall recycling rate, understood in this work as the percentage of plastic waste supplied to the market as secondary plastics (the market includes domestic and exports, the latter being calculated with the same reprocessing efficiency as in EU), was determined in this work for 2018 to stand at 24%. With the approach above it was projected to reach 40% in 2030. The value of components of the overall rate are given in Table S14.

In the Targets scenario narrative, the overall recycling rate is normative, given in the Packaging and Packaging Waste Directive (EU) 2018/852, respectively 50% in 2025 and 55% in 2030. The sorting and reprocessing rates were again marginally increased while the collection efficiency was raised close to maximum rates[60,61].

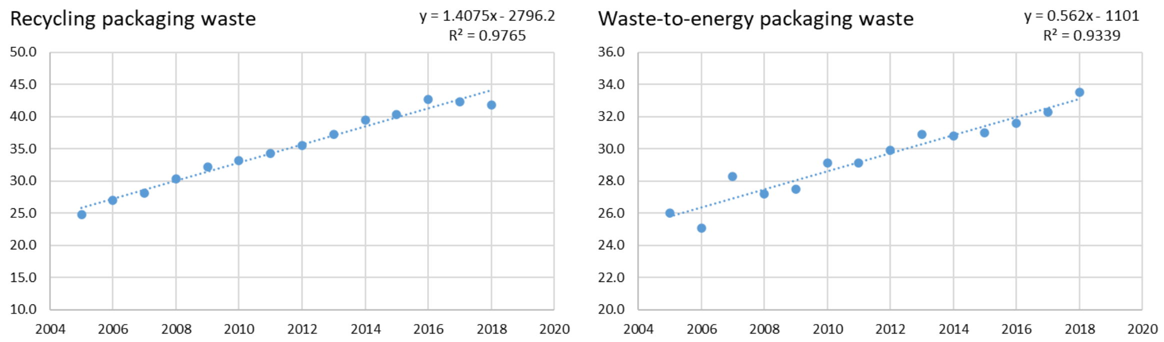

Figure S14: Linear trend for the development of recycling and WtE based on Eurostat data

In the Max potential scenario narrative, the recycling rate is finally raised to 70%. The components were based on a number of recent studies which collected data on a large number of separate collection systems[60], data reflecting sorting and reprocessing efficiencies in plants around Europe[62], and a study that explored in detail the conditions necessary for an outcome of maximum recycling for plastic packaging waste[61]. These studies show that the best performing municipal separate collection systems achieve 70% capture rates, while deposit-return systems can achieve capture rates of over 90%. Data collected from a number of sorting and mechanical recycling facilities in Europe, shows that in both cases efficiencies around 90% are close to the technical limit today[62], i.e., the rate or recovered materials. Brouwer et al. estimate that an overall recycling rate of 70% (taking household and industrial packaging waste) could be achieved, implying collection at 80%, and sorting and reprocessing efficiencies around 90%. However, this would imply the

implementation of packaging design changes, such as limiting types of polymers to the three most common (PE, PP, and PET), changing all black packaging to NIR recognizable colours, and reduction of laminated flexibles.

To project the evolution of landfilling, and by extension incineration, of remaining plastic packaging waste, which is collected mixed with other waste streams, we used (1) the linear trend revealed by the 2005-2018 statistics on disposal in the Baseline narrative, and (2) the normative target of 10% landfill by 2035, in the Targets and the Maximum potential narratives. EC Directive (EU) 2018/850 on the Landfill of Waste imposes an EU limit to landfill a maximum of 10% in 2035. Thus, the normative target for 2030 landfilling of plastic packaging waste would be 18-19% (decrease from 40% in 2018). However, the linear projection in the Baseline narrative reached 20%, and therefore we extended the target for 2030 at 10%.

Table S14: Evolution of separate collection, sorting and reprocessing efficiencies in the three scenario narratives, as well as the rate of disposal to WtE. Values as fraction denoting percentage.

Parameter	Scenarios	2018	2019	2020	2021	2022	2023	2024	2025	2026	2027	2028	2029	2030
**Separate collection efficiency**	Baseline	0.38	0.40	0.42	0.44	0.46	0.47	0.49	0.51	0.53	0.55	0.56	0.58	0.60
	Targets	0.38	0.43	0.46	0.51	0.57	0.63	0.68	0.71	0.73	0.74	0.74	0.75	0.75
	Max potential	0.38	0.43	0.47	0.51	0.56	0.62	0.67	0.71	0.74	0.76	0.78	0.79	0.80
**Sorting efficiency**	Baseline	0.78	0.78	0.78	0.79	0.79	0.79	0.79	0.79	0.79	0.80	0.80	0.80	0.80
	Targets	0.78	0.79	0.79	0.80	0.81	0.82	0.82	0.83	0.83	0.84	0.84	0.85	0.85
	Max potential	0.78	0.79	0.80	0.82	0.83	0.84	0.85	0.86	0.87	0.89	0.90	0.91	0.92
**Mechanical recycling efficiency**	Baseline	0.81	0.81	0.82	0.82	0.82	0.83	0.83	0.83	0.84	0.84	0.84	0.85	0.85
	Targets	0.81	0.81	0.82	0.83	0.83	0.84	0.84	0.85	0.85	0.85	0.86	0.86	0.86
	Max potential	0.81	0.82	0.83	0.84	0.86	0.87	0.88	0.89	0.90	0.91	0.93	0.94	0.95
**Waste-to-energy rate (share of disposal)**	Baseline	0.60	0.62	0.63	0.65	0.67	0.68	0.70	0.72	0.73	0.75	0.77	0.78	0.80
	Targets	0.60	0.63	0.65	0.68	0.70	0.73	0.75	0.78	0.80	0.83	0.85	0.88	0.90
	Max potential	0.60	0.63	0.65	0.68	0.70	0.73	0.75	0.78	0.80	0.83	0.85	0.88	0.90

Effects of consumption reduction measures

According to estimations by ICF and Eunomia (2018), packaging which falls under the Single Use Plastics (SUP) Directive amount to around 1,100 kt, of which the largest two categories, i.e. food containers and drink cups/lids, amount to 850 kt. These two categories are also subject to consumption reduction measures and in some cases, upcoming sales restrictions, e.g., articles of EPS. The SUP Directive implementation is expected to result in a 30% reduction in consumption by 2025 and of 50% by 2030, for these two categories[64].

Studies prepared for the impact assessment of the Carrier bag directive (Directive (EU) 2015/720), estimated total bag consumption between 1,100 to 1,600 kt in 2010 [65,66]. Of this, 750 kt was estimated as single use. There is no estimate for 2018. The directive adopted in 2015, mandated a reduction of consumption for bags between 15 and 50 microns, to 90 items/person by 2019 and 40 items/person by 2025. These reduction figures could translate roughly into 50% and 75% reduction. However, actual reduction in plastics amounts used to produce bags is likely to by lower, as consumption will move to multiple-use alternatives, as well as other materials such as paper.

Taking the above legislation outcomes, it is reasonable to assume a maximum reduction potential of around 1,000 kt, which is equivalent to around 5% of the packaging POM in 2018. The implementation of consumption reduction in the model framework occurs by changing the direct consumption of packaging by households in the Final Demand. To maintain total household consumption equal, the reduction in packaging is replaced with equal value products of the paper sector. This is a reasonable assumption, considering that a large share of SUP plastics will be substituted with other materials, paper being a prominent option.

Reduction of exports

In 2018, around 25% of recovered plastics were still exported for further treatment or recycling outside the EU. In late 2020, the EU adopted new tighter rules regarding export and import of plastic waste, amending the EU's Waste Shipment Regulation (Regulation (EC) No 1013/2006), and which go beyond implementing the decision taken by 187 countries in May 2019 at the 14th Conference of the Parties of the Basel Convention. There are yet voices calling for a full ban on plastic exports. Developing domestic infrastructure and markets for secondary plastics, are key commitments of both the European Green Deal and the new Circular Economy action plan.

EU-28 exports of plastics have decreased substantially between 2016 and 2020, primarily driven by the import ban announced in 2017 by China, which took effect in the beginning of 2018. As such European exports decreased with a yearly rate of around 20% in 2017 and 2018, which then decreased to around 10% in 2019 and 2020. This reflects that the market has adapted, with exports being rerouted to other countries.

We consider that exports will continue to decrease, mainly due to pressure from EU policy. As such we assumed a baseline development which maintains a yearly decrease rate of 10% up to 2030, while in the Max scenario, we implemented a gradual elimination of exports altogether by 2030.

Increased closed-loop recycling (within the packaging sector)

Closed-loop recycling can generally be defined as plastics flows of a specific product group in a specific sector being recycled into the same product group and sector. In the present work, we use closed-loop recycling more broadly, encompassing the use of secondary plastics into production of new packaging vs. their use in all other sectors (open-loop).

The present model in mixed units, incorporates material substitution both in a closed-loop and open-loop. Furthermore, the principle of value-corrected substitution as a means to account for different plastic quality levels, is directly implemented in the matrix of technical coefficients. Specifically, in the foreground production of packaging a physical unit of secondary plastics replaces an equal amount of primary plastic (reflecting closed-loop and high quality), while the amount of secondary plastics used in other sectors substitutes virgin plastics based on its unit monetary value, which is approx. 70% of the value of primary plastics (reflecting open-loop and lower quality).

In 2018, approximately 30% of secondary plastics were returned to packaging production. In the Baseline development scenario, we did not change this rate. In the Targets scenario, the rate is determined with the precondition that the recycled content mandated by the plastics industry, i.e., 30%, will be achieved in 2030. The rate of returned to packaging is calculated considering that the 2030 recycling rate is 55% and accounting for the difference between packaging use and waste generation (stock and trade). Lastly, in the Max potential

scenario, we implemented a gradual reduction in open-loop and increase in closed-loop utilization, up to a maximum of 90% of total quantity of secondary plastics. This raised the recycled content rate in plastic packaging production to 50-55%. Recent studies such as Andreasi Bassi et al.[67] and Eriksen et al.[68] summed literature and industry data, indicating that 50% to 90% recycled content is possible for individual plastic packaging applications.

Table S15: Evolution of export rates for sorted plastics and the rate of closed-loop use of recyclate in the three scenario narratives. Values as fraction denoting percentage.

Parameter	Scenarios	2018	2019	2020	2021	2022	2023	2024	2025	2026	2027	2028	2029	2030
Export rate	Baseline	0.26	0.24	0.21	0.19	0.17	0.15	0.14	0.13	0.11	0.10	0.09	0.08	0.07
	Targets	0.26	0.24	0.21	0.19	0.17	0.15	0.14	0.13	0.11	0.10	0.09	0.08	0.07
	Max potential	0.26	0.24	0.22	0.20	0.17	0.15	0.13	0.11	0.09	0.07	0.04	0.02	0.00
Closed loop recycling rate	Baseline	0.31	0.31	0.31	0.31	0.31	0.31	0.31	0.31	0.31	0.31	0.31	0.31	0.31
	Targets	0.31	0.34	0.37	0.40	0.43	0.45	0.48	0.51	0.54	0.57	0.59	0.62	0.65
	Max potential	0.31	0.36	0.41	0.46	0.51	0.56	0.61	0.66	0.70	0.75	0.80	0.85	0.90

3.3. Scenario model runs

The effect of the interventions implemented in the foreground scenarios, are measured by comparison of outcomes in the intervention scenarios with those occurring in a Reference scenario. The Reference scenario consists of the above-described projection of background conditions, with the foreground system continuing business as usual. To calculate the outcomes of the reference and alternative scenarios, we apply standard input-output analysis using the exogenously determined changes in final demand and the multiplier matrix based on the Leontief demand model. As such, we aim to capture direct and indirect effects, and, similarly to previous studies, do not model induced effects in the economy[69].

$$\mathbf{c}^* = \mathrm{diag}(\mathbf{b}^*)(\mathbf{I} - \mathbf{Asu}^*)^{-1}\mathbf{y}^* \tag{S8}$$

$$\Delta\mathbf{c} = \mathbf{c}^* - \mathbf{c} \tag{S9}$$

Equation S8 and S9 show how the differences between any alternative scenario (*) and the reference scenario, in a year, are calculated. **c** stands for the vector of absolute values of the three metrics used (GHGs, employment and value added) occurring in each sector of the economy, as an outcome of potential changes in sector intensities (**b**), the matrix of technical coefficients ($\mathbf{A}_{\mathbf{su}}$), and the demand vector (**y)**, which is the sum of **FD**+(-**m**). Thus, we account for effects/impacts in the domestic economy and do not include the effects of produced imports. Imports are maintained fixed in a specific year over all scenarios, and therefore do not contribute to net changes. In this way we capture the full effect of interventions, without making specific assumptions on the conditions in countries supplying these imports.

The system is constructed and projected in constant prices. The system is projected every year until 2030, and the adoption of the different interventions is implemented largely in a linear manner, with target values achieved in the final year.

When implementing exogenous changes in the **A** and **y**, the economy monetary representation becomes unbalanced at the sectoral level (i.e., input of sectors $\neq$ output of sectors). For example, a main imbalance is created when changing the share of waste management options over time in scenarios, as they carry different costs. Therefore, the model rebalances both at sector level and final demand, the former by adjustments in value added or rescaling of sector inputs as column entries (Scott et al., 2019; Wiebe et al., 2019).

The implementation of foreground scenarios implies quite many changes in the matrix of technical coefficients and the final demand. Changes in separate collection efficiencies imply use coefficient changes in all sectors and for all waste management services, as well as in the final demand. Furthermore, increasing efficiencies in sorting and reprocessing sectors, results in larger coefficients for useful outputs and a decrease in outputs that require treatment (residuals). All these changes create imbalances in sectors, which can be seen when converting all use coefficient to the same reference unit (monetary) as the sectoral sum of use coefficients and value added does not equal to 1.

Thus, the system is rebalanced at sectoral and final demand levels in each modelled year [56,69]. Specifically, the costs differences related to waste management incurred by all sectors of the economy is reallocated to value added. This assumes that an increase in waste management costs will affect sectoral operating surplus. The effect of efficiency changes in the sorting and reprocessing sectors is a decrease in expenditure per unit produced. This effect could be similarly reallocated to value added, however, it is more realistic that modelled increases in efficiency come at the cost of increased inputs, investment in infrastructure and research/innovation. As we do not know where this expenditure occurs, it was reallocated over all inputs to these two sectors.

The increasing share of secondary plastics in packaging conversion leads to a decrease in feedstock costs in this sector. The difference in the case of the conversion sector was added to value added. Lastly, in the case of final demand categories (except exports), increasing costs with waste management were assumed to affect consumption of other background products. This demand decrease was applied homogeneously to all products. The changes in consumption of background products results in a further decrease in associated plastic packaging waste. This additional effect is accounted by correcting waste amounts.

4 Additional result figures

Figure S15 illustrates the contribution made by the secondary sector in the base system in 2018. The net values represent the change in the system when secondary operations are removed from the system (essentially their coefficients are turned to 0 values). As seen, this results in an increase of GHG emissions and loss in employment.

The contribution of plastics recovery and recycling in the reference 2018 system was estimated by calculating impacts after removing separate collection in the model and adjusting for the associated reduction in waste management costs. Thus, an alternative reference without recycling, with treatment based only on WtE and landfill, incurred an additional 5 Mt CO2-eq., a decrease of around 35,000 employed persons, and a small addition to value added of 85 MEUR.

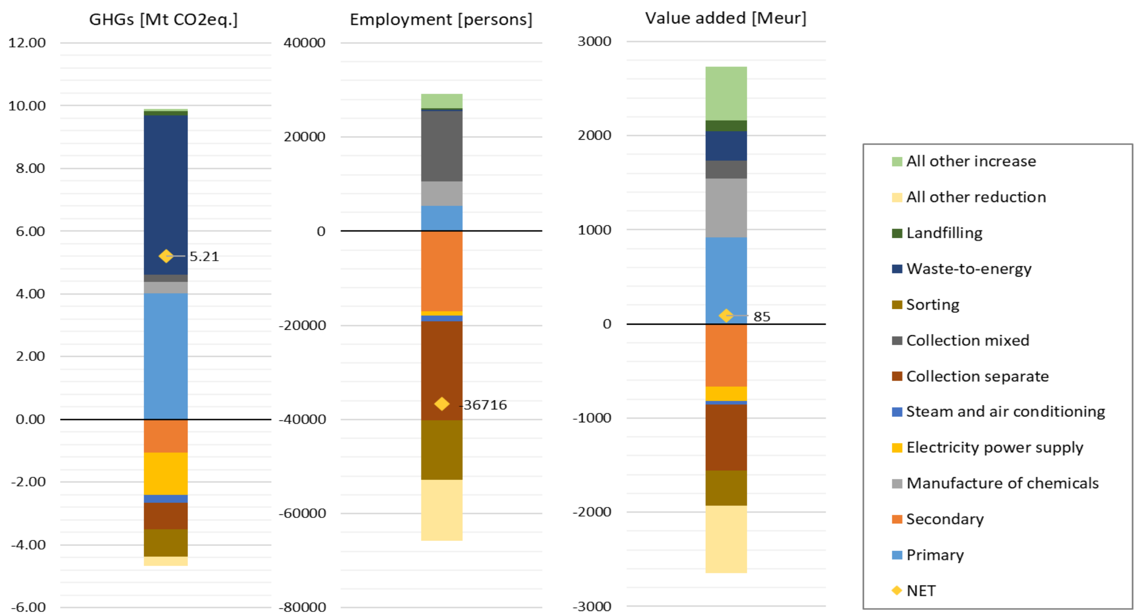

Figure S15: Sector contribution to GHGs, employment and value added changes to a reference for 2018 without separate collection, sorting and recycling.

Figure S16 illustrates total (direct and indirect) value added of the plastic packaging system, and main activities contribution. The unit is MEUR.

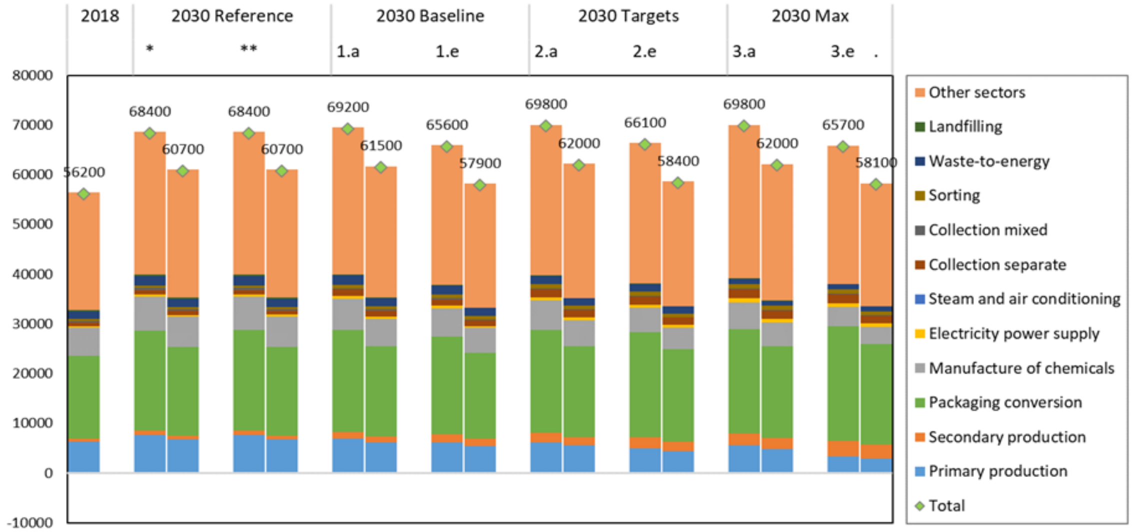

Figure S16: Total (direct and indirect) value added of the plastic packaging system, and main activities contribution. Each scenario is illustrated as two stacked bars, the right and left bars represent results for the system with and respectively without packaging intensity decrease.

The following four Sankey diagrams illustrate plastic packaging flows through the EU-28 system for the year 2030, for the model variation which included a decrease of product packaging intensity over time.

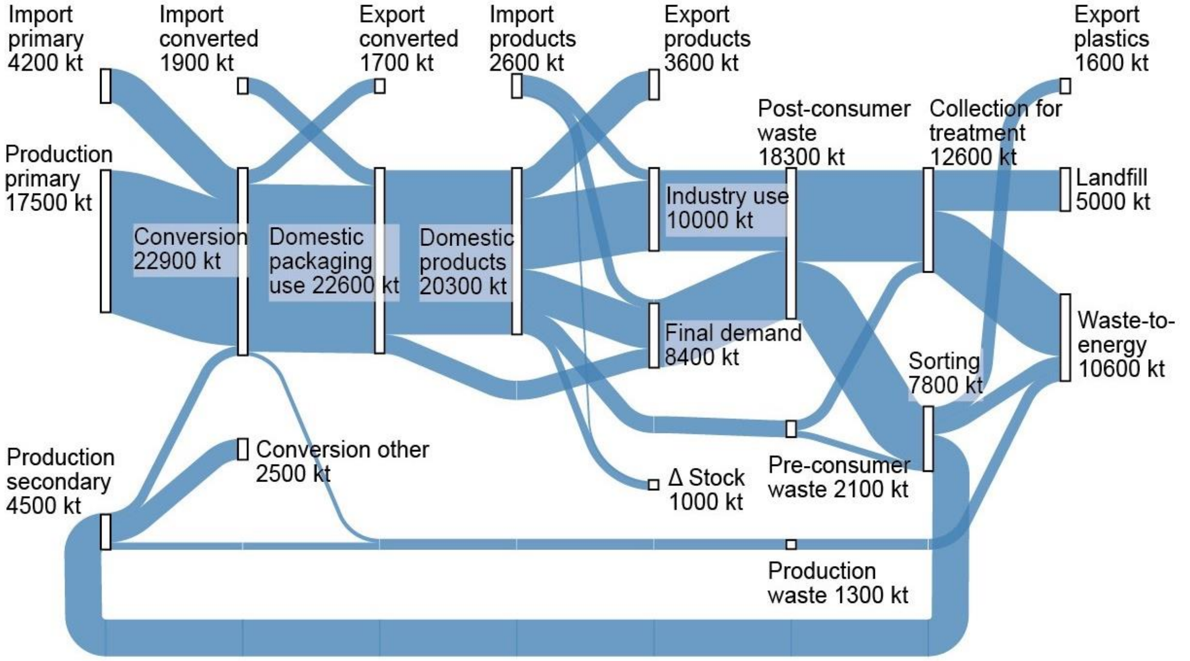

Figure S17: 2030 Refence (BAU scenario) EU-28 flows for the plastic packaging system [kt y^{-1}], values denote process totals and are rounded to two/three significant digits. Scenario variation which included a decrease of product packaging intensity over time.

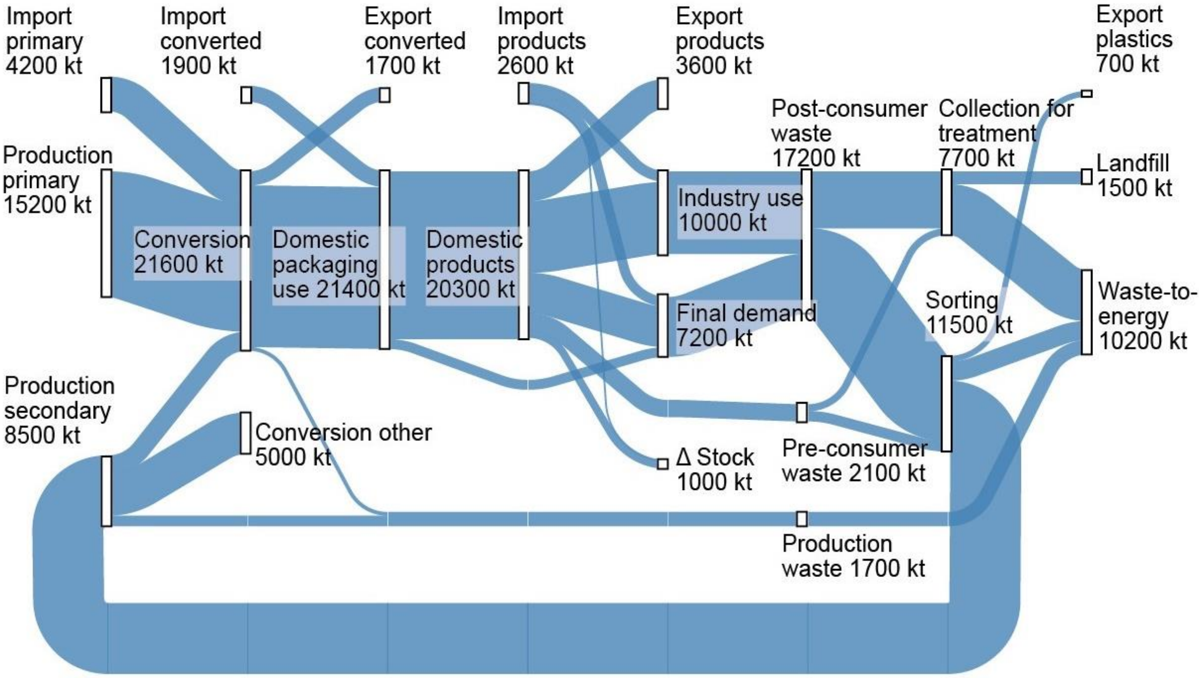

Figure S18: 2030 Baseline development scenario EU-28 flows for the plastic packaging system [kt y-1], values denote process totals and are rounded to two/three significant digits. Scenario variation which included a decrease of product packaging intensity over time.

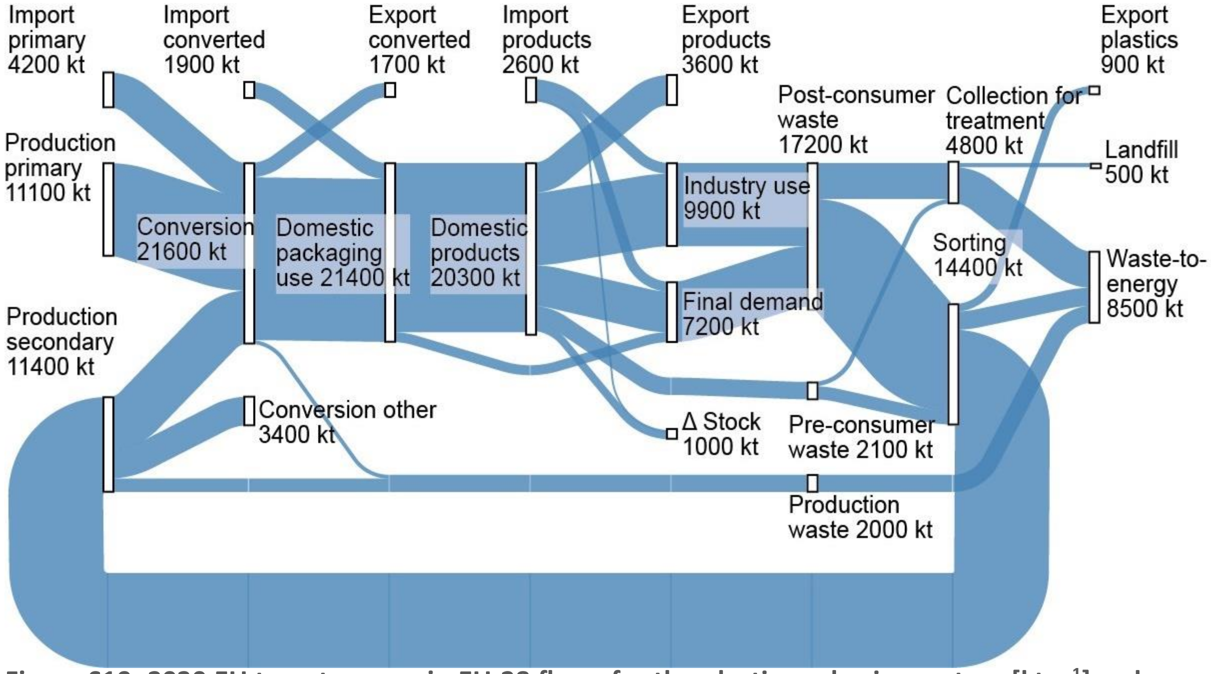

Figure S19: 2030 EU targets scenario EU-28 flows for the plastic packaging system [kt y^{-1}], values denote process totals and are rounded to two/three significant digits. Scenario variation which included a decrease of product packaging intensity over time.

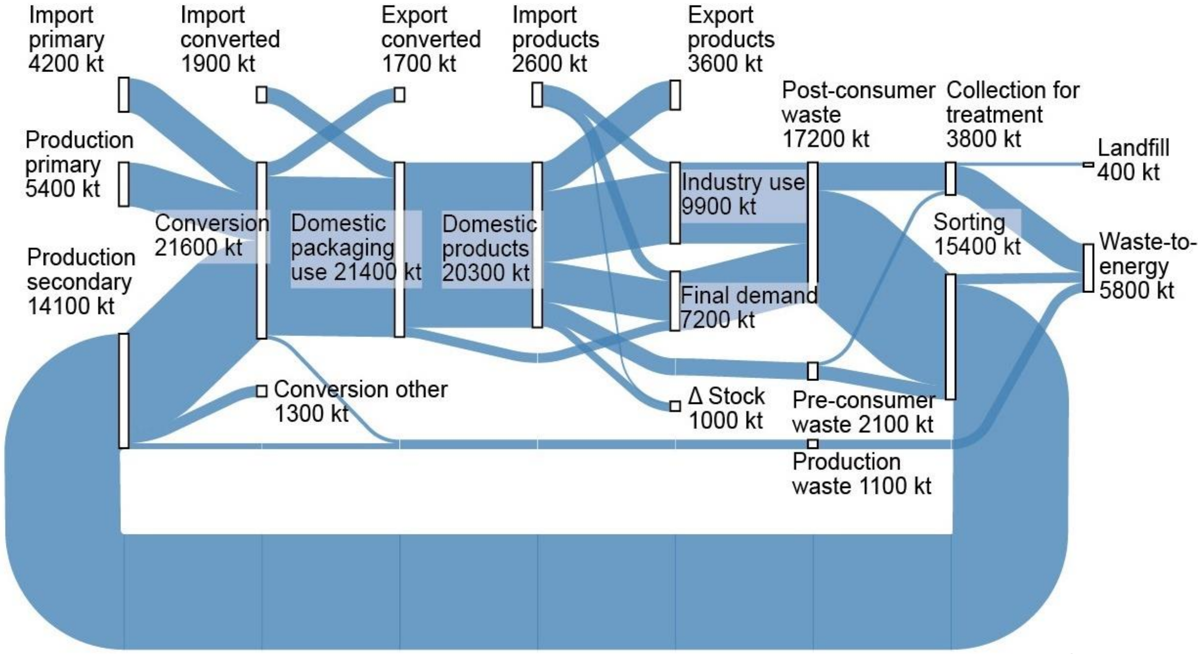

Figure 20: 2030 Max potential scenario EU-28 flows for the plastic packaging system [kt y^{-1}], values denote process totals and are rounded to two/three significant digits. Scenario variation which included a decrease of product packaging intensity over time.

The following figures present the results for sector contributions to changes from the Reference (BAU) scenario, for all sub-scenario variants. The values behind the figures are available in table format in the SI B file.

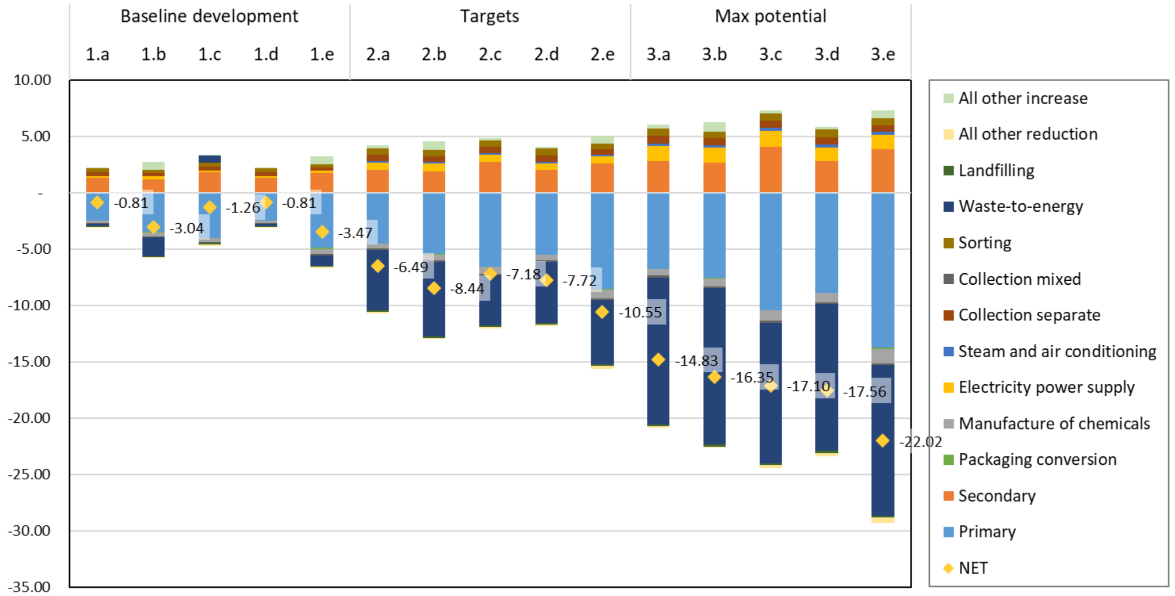

Figure S21: Sectoral contribution to GHG emissions in 2030 [Mt CO2eq.], scenarios without packaging intensity decrease. The results are calculated as difference in 2030 from the Refence (BAU) scenario (represented by the zero line).

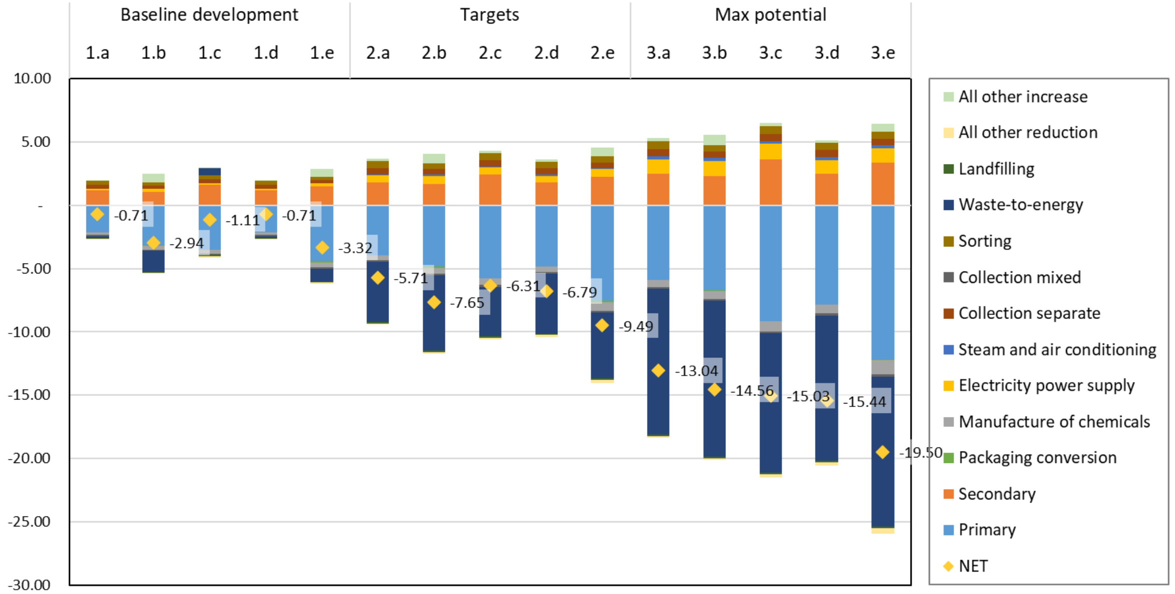

Figure S22: Sectoral contribution to GHG emissions in 2030 [Mt CO2eq.], scenarios with packaging intensity decrease. The results are calculated as difference in 2030 from the Refence (BAU) scenario (represented by the zero line).

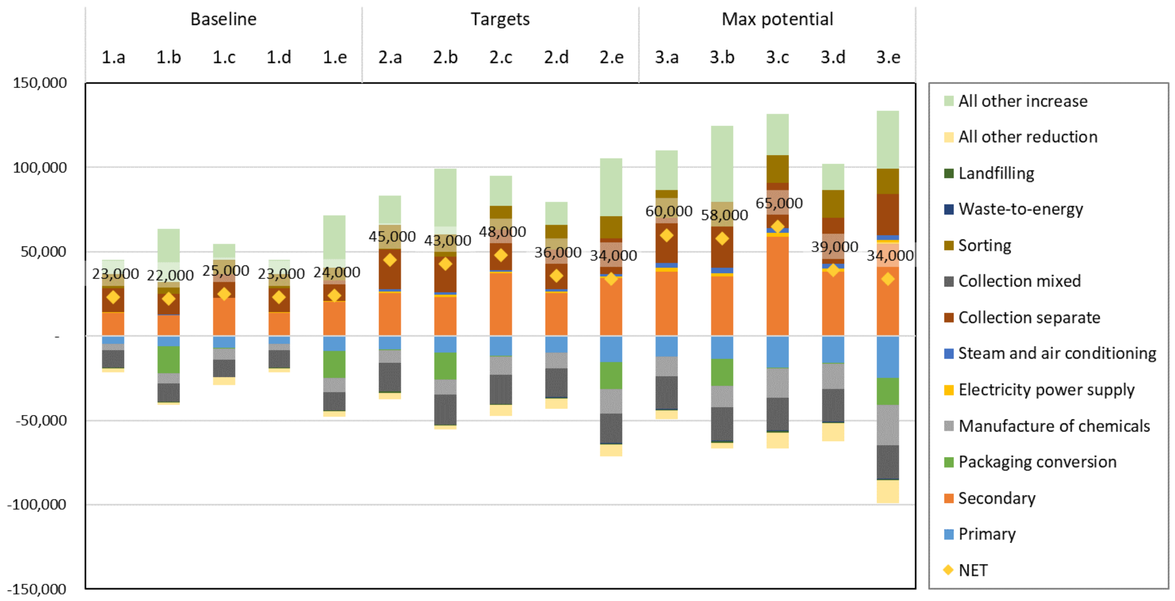

Figure S23: Sectoral contribution to employment in 2030 [person positions], scenarios without packaging intensity decrease. The results are calculated as difference in 2030 from the Refence (BAU) scenario (represented by the zero line).

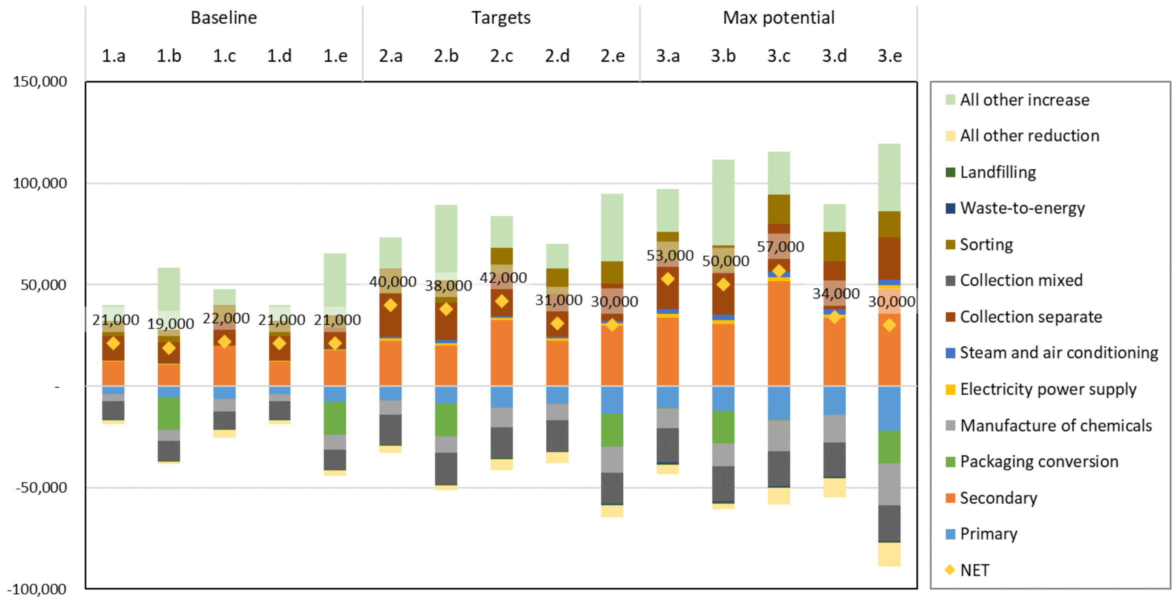

Figure S24: Sectoral contribution to employment in 2030 [person positions], scenarios with packaging intensity decrease. The results are calculated as difference in 2030 from the Refence (BAU) scenario (represented by the zero line).

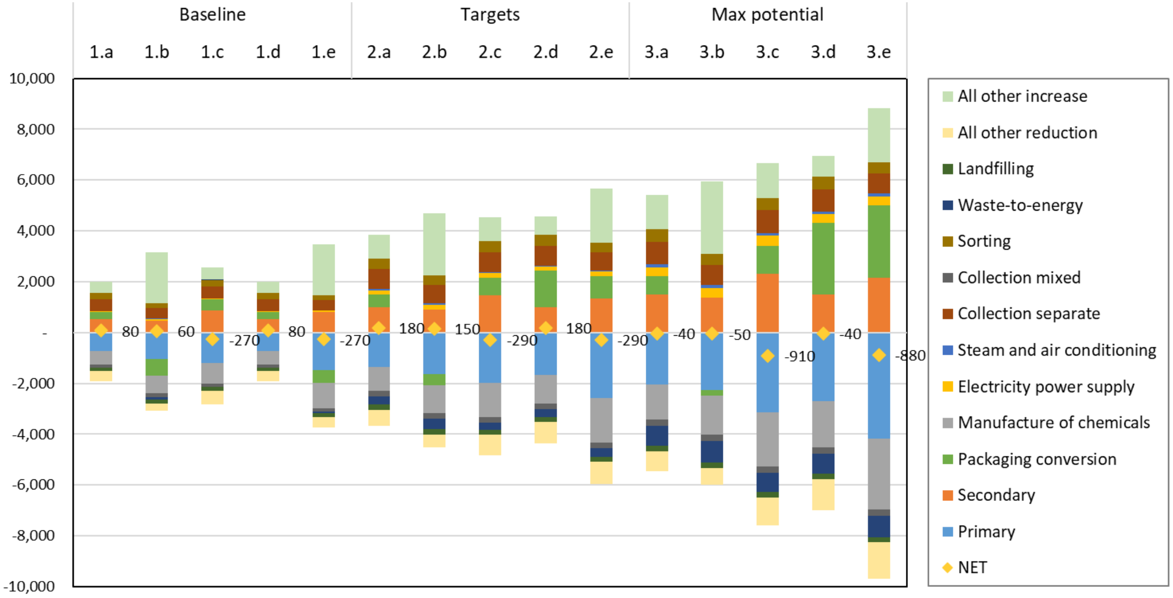

Figure S25: Sectoral contribution to value added in 2030 [MEUR], scenarios without packaging intensity decrease. The results are calculated as difference in 2030 from the Refence (BAU) scenario (represented by the zero line).

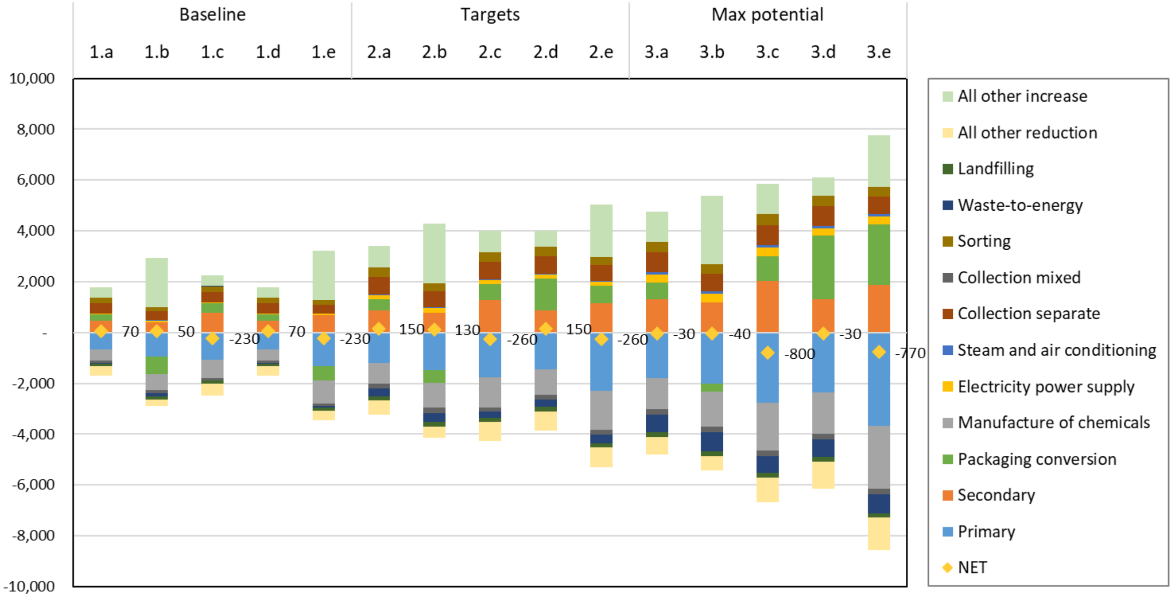

Figure S26: Sectoral contribution to value added in 2030 [MEUR], scenarios with packaging intensity decrease. The results are calculated as difference in 2030 from the Refence (BAU) scenario (represented by the zero line).

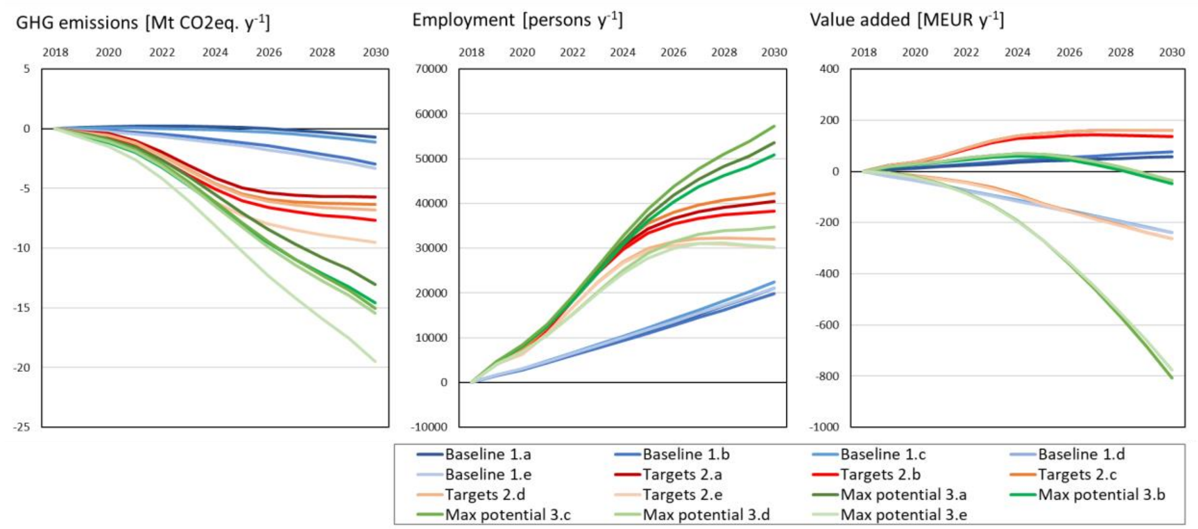

Figure S27: Net difference between intervention scenarios and the reference (BAU) scenario (represented by the zero line) over the 12 years period: GHG emissions, employment and value added. Scenario variants are noted a-e. Results are for scenarios with decreasing packaging intensity.

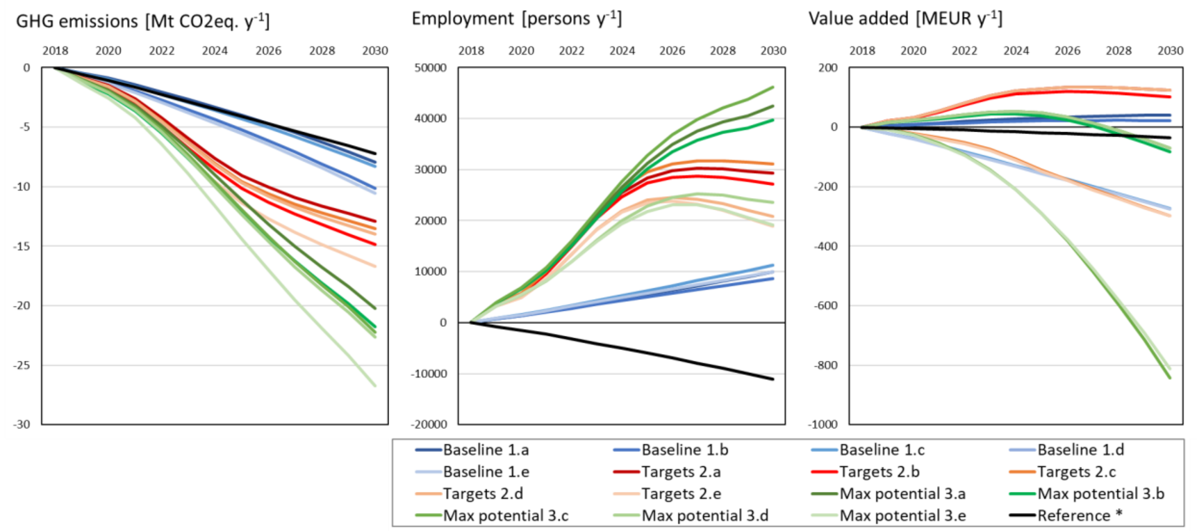

Figure S28: Net difference between intervention scenarios with decreasing product packaging intensity and the reference (BAU) scenario with constant intensities (represented by the zero line) over the 12 years period: GHG emissions, employment and value added. Scenario variants are noted a-e. The reference with intensity decrease is shown with a black line.

The last figure below, displays results of sensitivity model runs, testing the assumption of fixed average prices for secondary plastics over the assessment period. An increase in price would result in higher costs for the conversion sector, that could be accounted as diminishing profits. In terms of modelling, a change in price would mean also changing the reprocessing (secondary production) sector by increasing inputs and its own value added. Thus, diminishing value added in the conversion sector may partly or fully be compensated by economic activity in other sectors.

We checked effects of this as a sensitivity run. Specifically, the price of secondary plastics, that are recycled in closed loop, was raised each year to reach par with primary plastics in 2030. The structure the secondary sector was also modified to account to the increase in price, with an increase of all intermediate inputs and value added. The results are presented in Figure S29. As expected, losses in value added in the conversion sector were compensated by the increase in inputs in the secondary sector, and there were also associated small gains in employment as well as small increases in GHG emissions. Overall there was a significant level of sensitivity to secondary plastic prices.

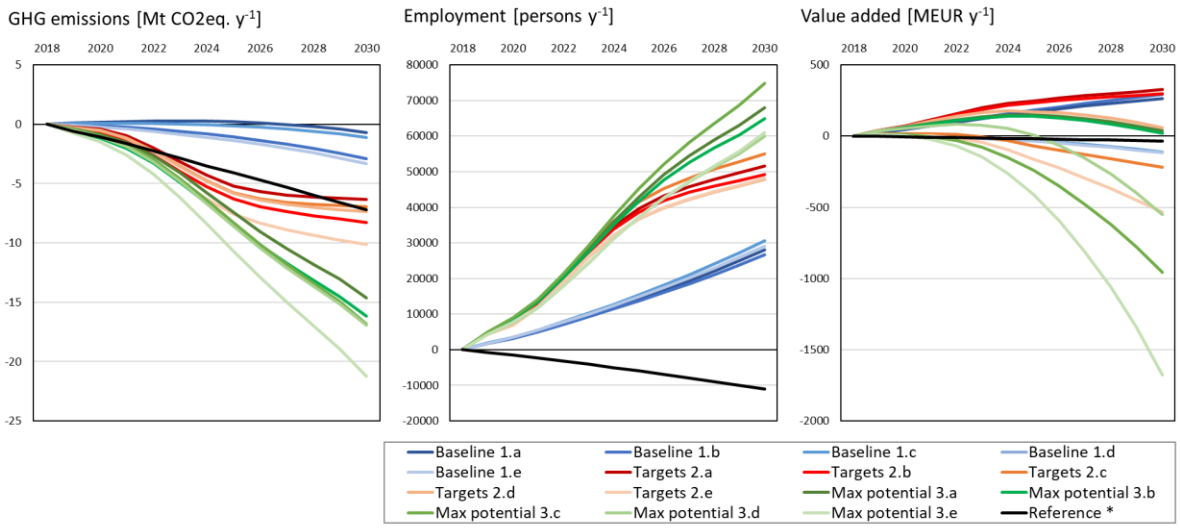

Figure S29: Sensitivity results – increasing price of recycled secondary plastics. Net difference between intervention scenarios and the reference (BAU) scenario (represented by the zero line) over the 12 years period: GHG emissions, employment and value added. Scenario variants are noted a-e. Results are for scenarios with constant packaging intensity.

References

(1) Nakamura, S.; Kondo, Y. Input-Output Analysis of Waste Management. *J. Ind. Ecol.* **2002**, *6* (1), 39–63. https://doi.org/10.1162/108819802320971632.

(2) Lenzen, M.; Reynolds, C. J. A Supply-Use Approach to Waste Input-Output Analysis. *J. Ind. Ecol.* **2014**, *18* (2), 212–226. https://doi.org/10.1111/jiec.12105.

(3) PlasticsEurope. *Plastics - the Facts 2019. An Analysis of European Plastics Production, Demand and Waste Data*; Brussels, 2019.

(4) Emballasjeforeningen. *Veikart for Sirkulær Plastemballasje i Norge [Roadmap for Circular Plastic Packaging in Norway]*; Oslo, 2019.

(5) EUROMAP. *Plastics Resin Production and Consumption in 63 Countries Worldwide 2009-2020*; Frankfurt am Main, 2016.

(6) UNSD. *United Nations Commodity Trade Statistics Database (UN Comtrade)*. United Nations Statistical Division. https://comtrade.un.org/ (accessed 2019-11-15).

(7) BIO Intelligence Service. *Plastic Waste in the Environment - Final Report under Framework Contract ENV.G.4/FRA/2008/0112*; Brussels, 2011.

(8) Conversio. *Final Report - Circular Economy of Plastics 2018 EU28 + 2*; Brussels, Belgium, 2019.

(9) Kawecki, D.; Scheeder, P. R. W.; Nowack, B. Probabilistic Material Flow Analysis of Seven Commodity Plastics in Europe. *Environ. Sci. Technol.* **2018**, *52* (17), 9874–9888. https://doi.org/10.1021/acs.est.8b01513.

(10) Nakamura, S.; Nakajima, K.; Kondo, Y.; Nagasaka, T. The Waste Input-Output Approach to Materials Flow Analysis: Concepts and Application to Base Metals. *J. Ind. Ecol.* **2007**, *11* (4), 50–63. https://doi.org/10.1162/jiec.2007.1290.

(11) Nakatani, J.; Maruyama, T.; Moriguchi, Y. Revealing the Intersectoral Material Flow of Plastic Containers and Packaging in Japan. *Proc. Natl. Acad. Sci.* **2020**, *117* (33), 19844–19853. https://doi.org/10.1073/pnas.2001379117.

(12) Hsu, W.-T.; Domenech, T.; McDowall, W. How Circular Are Plastics in the EU?: MFA of Plastics in the EU and Pathways to Circularity. *Clean. Environ. Syst.* **2021**, *2*, 100004. https://doi.org/10.1016/j.cesys.2020.100004.

(13) Conversio. *Kurzfassung Stoffstrombild Kunststoffe in Deutschland 2017 [Abstract Material Flows for Plastics in Germany 2017]*; Mainaschaff, 2018.

(14) Van Eygen, E.; Feketitsch, J.; Laner, D.; Rechberger, H.; Fellner, J. Comprehensive Analysis and Quantification of National Plastic Flows: The Case of Austria. *Resour. Conserv. Recycl.* **2017**, *117*, 183–194. https://doi.org/10.1016/j.resconrec.2016.10.017.

(15) Hogg, D.; Elliott, T.; Corbin, M.; Hilton, M.; Tsiarta, C.; Hudson, J.; Vives, R.; Sastre, S.; Campos, L.; Puig, I.; Šleinotaitė-Budrienė, L.; Lippa, M.; Kazlauskaitė, L. *Study on Waste Statistics - A Comprehensive Review of Gaps and Weaknesses and Key Priority Areas for Improvement in the EU Waste Statistics*; Brussels, 2017.

(16) ICF; Eunomia. *Plastics - Reuse, Recycling and Marine Litter : Final Report*; Brussels, 2018. https://doi.org/10.2779/724160.

(17) Eunomia. *Plastic Packaging: Shedding Light on the UK Data*; Bristol, 2018.

(18) Lopez-Aguilar, J. F.; Sevigné-Itoiz, E.; Maspoch, M. L.; Peña, J. A Realistic Material Flow Analysis for End-of-Life Plastic Packaging Management in Spain: Data Gaps and Suggestions for Improvements towards Effective Recyclability. *Sustain. Prod. Consum.* **2022**, *31*, 209–219. https://doi.org/10.1016/J.SPC.2022.02.011.

(19) Thomassen, G.; Van Passel, S.; Alaerts, L.; Dewulf, J. Retrospective and Prospective Material Flow Analysis of the Post-Consumer Plastic Packaging Waste Management System in Flanders. *Waste Manag.* **2022**, *147*, 10–21. https://doi.org/10.1016/J.WASMAN.2022.05.004.

(20) PlasticsEurope. *Plastics – the Facts 2015. An Analysis of European Plastics Production, Demand and Waste Data*; Brussels, 2015.
(21) Hestin, M.; Faninger, T.; Milios, L. *Increased EU Plastics Recycling Targets: Environmental, Economic and Social Impact Assessment - Final Report*; Brussels, 2015.
(22) Cimpan, C.; Bjelle, E. L.; Strømman, A. H. Plastic Packaging Flows in Europe: A Hybrid Input-output Approach. *J. Ind. Ecol.* **2021**, *25* (6), 1572–1587. https://doi.org/10.1111/jiec.13175.
(23) Tsarouhas, P. H. Evaluation of Overall Equipment Effectiveness in the Beverage Industry: A Case Study. *Int. J. Prod. Res.* **2013**, *51* (2), 515–523. https://doi.org/10.1080/00207543.2011.653014.
(24) Dora, M.; Wesana, J.; Gellynck, X.; Seth, N.; Dey, B.; De Steur, H. Importance of Sustainable Operations in Food Loss: Evidence from the Belgian Food Processing Industry. *Ann. Oper. Res.* **2019**. https://doi.org/10.1007/s10479-019-03134-0.
(25) Nessi, S.; Bulgheroni, C.; Konti, A.; Sinkko, T.; Tonini, D.; Pant, R. *Comparative LCA of Alternative Feedstock for Plastic Production - Draft Report for Stakeholder Consultation (Part 1)*; Brussels, Belgium, 2018.
(26) Eurostat. Packaging Waste by Waste Operations and Waste Flow [Env_waspac]. Statistical Office of the European Union: Luxembourg.
(27) Eunomia. *PET Market in Europe - State of Play: Production, Collection and Recycling Data*; Brussels, 2020.
(28) Eunomia. *Flexible Films Market in Europe - State of Play: Production, Collection and Recycling Data*; Brussels, 2020.
(29) Eunomia. *HDPE & PP Market in Europe - State of Play: Production, Collection and Recycling Data*; Brussels, 2020.
(30) Deloitte. *Blueprint for Plastics Packaging Waste: Quality Sorting & Recycling - Final Report*; 2017.
(31) EUMEPS. EPS Recycling in Europe - an Inventory. European Manufacturers of Expanded Polystyrene: Maaseik 2018.
(32) Cimpan, C.; Maul, A.; Jansen, M.; Pretz, T.; Wenzel, H. Central Sorting and Recovery of MSW Recyclable Materials: A Review of Technological State-of-the-Art, Cases, Practice and Implications for Materials Recycling. *J. Environ. Manage.* **2015**, *156*, 181–199. https://doi.org/10.1016/j.jenvman.2015.03.025.
(33) Cimpan, C.; Maul, A.; Wenzel, H.; Pretz, T. Techno-Economic Assessment of Central Sorting at Material Recovery Facilities – the Case of Lightweight Packaging Waste. *J. Clean. Prod.* **2016**, *112*, 4387–4397. https://doi.org/10.1016/j.jclepro.2015.09.011.
(34) European Court of Auditors. *EU Action to Tackle the Issue of Plastic Waste 2020*; Luxembourg, 2020.
(35) Vendries Algarin, J.; Hawkins, T. R.; Marriott, J.; Scott Matthews, H.; Khanna, V. Disaggregating the Power Generation Sector for Input-Output Life Cycle Assessment. *J. Ind. Ecol.* **2015**, *19* (4), 666–675. https://doi.org/10.1111/jiec.12207.
(36) Hawkins, T.; Hendrickson, C.; Higgins, C.; Matthews, H. S.; Suh, S. A Mixed-Unit Input-Output Model for Environmental Life-Cycle Assessment and Material Flow Analysis. *Environ. Sci. Technol.* **2007**, *41* (3), 1024–1031. https://doi.org/10.1021/es060871u.
(37) Stadler, K.; Wood, R.; Bulavskaya, T.; Södersten, C. J.; Simas, M.; Schmidt, S.; Usubiaga, A.; Acosta-Fernández, J.; Kuenen, J.; Bruckner, M.; Giljum, S.; Lutter, S.; Merciai, S.; Schmidt, J. H.; Theurl, M. C.; Plutzar, C.; Kastner, T.; Eisenmenger, N.; Erb, K. H.; de Koning, A.; Tukker, A. EXIOBASE 3: Developing a Time Series of Detailed Environmentally Extended Multi-Regional Input-Output Tables. *J. Ind. Ecol.*

2018, *22* (3), 502–515. https://doi.org/10.1111/jiec.12715.
(38) Plastics Recyclers Europe. *Report on Plastics Recycling Statistics*; Brussels, 2020.
(39) Marques, R. C.; da Cruz, N. F.; Simoes, P.; Ferreira, S.; Pereira, M. C.; de Jaeger, S.; Rigamonti, L.; Grosso, M.; Ongongo, F.; Williams, I. *Final Report: Cost and Benefits of Packaging Waste Recycling*; European Investment Bank, 2014.
(40) da Cruz, N. F.; Ferreira, S.; Cabral, M.; Simoes, P.; Marques, R. C. Packaging Waste Recycling in Europe: Is the Industry Paying for It? *Waste Manag.* **2014**, *34* (2), 298–308. https://doi.org/10.1016/j.wasman.2013.10.035.
(41) Andreasi Bassi, S.; Boldrin, A.; Faraca, G.; Astrup, T. F. Extended Producer Responsibility: How to Unlock the Environmental and Economic Potential of Plastic Packaging Waste? *Resour. Conserv. Recycl.* **2020**, *162*, 105030. https://doi.org/10.1016/j.resconrec.2020.105030.
(42) Rodrigues, J. F. D.; Lorena, A.; Costa, I.; Ribeiro, P.; Ferrão, P. An Input-Output Model of Extended Producer Responsibility. *J. Ind. Ecol.* **2016**, *20* (6), 1273–1283. https://doi.org/10.1111/jiec.12401.
(43) Gradus, R. H. J. M.; Nillesen, P. H. L.; Dijkgraaf, E.; van Koppen, R. J. A Cost-Effectiveness Analysis for Incineration or Recycling of Dutch Household Plastic Waste. *Ecol. Econ.* **2017**, *135*, 22–28. https://doi.org/10.1016/j.ecolecon.2016.12.021.
(44) Tonini, D.; Garcia-gutierrez, P.; Nessi, S. *Environmental Effects of Plastic Waste Recycling*; Luxembourg, 2021. https://doi.org/10.2760/955772.
(45) Nessi, S.; Bulgheroni, C.; Garbarino, E.; Garcia-Gutierrez, P.; Orveillon, G.; Sinkko, T.; Tonini, D.; Pant, R. *Comparative LCA of Alternative Feedstock for Plastic Production - Draft Report for Stakeholder Consultation (Part 2)*; Brussels, Belgium, 2018.
(46) IEA. *The Future of Petrochemicals - Towards More Sustainable Plastics and Fertilisers*; OECD, 2018. https://doi.org/10.1787/9789264307414-en.
(47) Franklin Associates. *Cradle-to-Resin Life Cycle Analysis of Polyethylene Terephthalate Resin- Final Revised Report*; 2020.
(48) Franklin Associates. *Cradle-To-Gate Life Cycle Inventory of Nine Plastic Resins*; 2011.
(49) Franklin Associates. *Life Cycle Impacts for Postconsumer Recycled Resins: PET, HDPE, and PP*; 2018.
(50) Lenzen, M.; Rueda-Cantuche, J. M. A Note on the Use of Supply-Use Tables in Impact Analyses. *Sort* **2012**, *36* (2), 139–152.
(51) Wiebe, K. S.; Bjelle, E. L.; Többen, J.; Wood, R. Implementing Exogenous Scenarios in a Global MRIO Model for the Estimation of Future Environmental Footprints. *J. Econ. Struct.* **2018**, *7* (1), 20. https://doi.org/10.1186/s40008-018-0118-y.
(52) European Commission. *European Economic Forecast: Spring 2021*; 2021. https://doi.org/10.2765/66679.
(53) European Commission. *European Economic Forecast Autumn 2021*; 2021. https://doi.org/10.2765/199989.
(54) O’Neill, B. C.; Kriegler, E.; Ebi, K. L.; Kemp-Benedict, E.; Riahi, K.; Rothman, D. S.; van Ruijven, B. J.; van Vuuren, D. P.; Birkmann, J.; Kok, K.; Levy, M.; Solecki, W. The Roads Ahead: Narratives for Shared Socioeconomic Pathways Describing World Futures in the 21st Century. *Glob. Environ. Chang.* **2017**, *42*, 169–180. https://doi.org/10.1016/j.gloenvcha.2015.01.004.
(55) Vrontisi, Z.; Fragkiadakis, K.; Kannavou, M.; Capros, P. Energy System Transition and Macroeconomic Impacts of a European Decarbonization Action towards a below 2 °C Climate Stabilization. *Clim. Change* **2020**, *162* (4), 1857–1875.

https://doi.org/10.1007/s10584-019-02440-7.
(56) Scott, K.; Giesekam, J.; Barrett, J.; Owen, A. Bridging the Climate Mitigation Gap with Economy-Wide Material Productivity. *J. Ind. Ecol.* **2019**, *23* (4), 918–931. https://doi.org/10.1111/jiec.12831.
(57) Gibon, T.; Wood, R.; Arvesen, A.; Bergesen, J. D.; Suh, S.; Hertwich, E. G. A Methodology for Integrated, Multiregional Life Cycle Assessment Scenarios under Large-Scale Technological Change. *Environ. Sci. Technol.* **2015**, *49* (18), 11218–11226. https://doi.org/10.1021/acs.est.5b01558.
(58) European Commission. *EU Reference Scenario 2020 - Energy, Transport and GHG Emissions Trends to 2050*; Brussels, 2021.
(59) European Commission. *Guidance for the Compilation and Reporting of Data on Packaging and Packaging Waste According to Decision 2005/270/EC*; Brussels, Belgium, 2020.
(60) Tallentire, C. W.; Steubing, B. The Environmental Benefits of Improving Packaging Waste Collection in Europe. *Waste Manag.* **2020**, *103*, 426–436. https://doi.org/10.1016/j.wasman.2019.12.045.
(61) Brouwer, M. T.; Thoden van Velzen, E. U.; Ragaert, K.; ten Klooster, R. Technical Limits in Circularity for Plastic Packages. *Sustainability* **2020**, *12* (23), 10021. https://doi.org/10.3390/su122310021.
(62) Antonopoulos, I.; Faraca, G.; Tonini, D. Recycling of Post-Consumer Plastic Packaging Waste in EU: Process Efficiencies, Material Flows, and Barriers. *Waste Manag.* **2021**, *126*, 694–705. https://doi.org/10.1016/j.wasman.2021.04.002.
(63) ICF; Eunomia. *Assessment of Measures to Reduce Marine Litter from Single Use Plastics*; Brussels, 2018.
(64) European Commission. *Impact Assessment: Reducing Marine Litter: Action on Single Use Plastics and Fishing Gear. Proposal for a Directive of the European Parliament and of the Council on the Reduction of the Impact of Certain Plastic Products on the Environment*; Brussels, 2018; Vol. SWD(2018).
(65) BIO Intelligence Service. *Assessment of Impacts of Options to Reduce the Use of Single-Use Plastic Carrier Bags*; Paris, 2011.
(66) Sherrington, C.; Hogg, D.; Jones, P.; Doswell, B.; Cullen, C.; Cole, G. *Assistance to the Commission to Complement an Assessment of the Socio-Economic Costs and Benefits of Options to Reduce the Use of Single-Use Plastic Carrier Bags in the EU*; Bristol, 2012.
(67) Andreasi Bassi, S.; Tonini, D.; Saveyn, H.; Astrup, T. F. Environmental and Socioeconomic Impacts of Poly(Ethylene Terephthalate) (PET) Packaging Management Strategies in the EU. *Environ. Sci. Technol.* **2022**, *56* (1), 501–511. https://doi.org/10.1021/ACS.EST.1C00761/SUPPL_FILE/ES1C00761_SI_002.XLSX.
(68) Eriksen, M. K.; Christiansen, J. D.; Daugaard, A. E.; Astrup, T. F. Closing the Loop for PET, PE and PP Waste from Households: Influence of Material Properties and Product Design for Plastic Recycling. *Waste Manag.* **2019**, *96*, 75–85. https://doi.org/10.1016/j.wasman.2019.07.005.
(69) Wiebe, K. S.; Harsdorff, M.; Montt, G.; Simas, M. S.; Wood, R. Global Circular Economy Scenario in a Multiregional Input–Output Framework. *Environ. Sci. Technol.* **2019**, *53* (11), 6362–6373. https://doi.org/10.1021/acs.est.9b01208.
